# Devonian northward drift of the Qaidam–Kunlun continent constrains the early evolution of the Paleo-Tethys Ocean

**DOI:** 10.1093/nsr/nwag131

**Published:** 2026-03-03

**Authors:** Peiping Song, Lin Ding, Chen Wu, Andrew V Zuza, Liyun Zhang, Yahui Yue, Jing Xie

**Affiliations:** State Key Laboratory of Tibetan Plateau Earth System, Environment and Resources (TPESER), Institute of Tibetan Plateau Research, Chinese Academy of Sciences, Beijing 100101, China; State Key Laboratory of Tibetan Plateau Earth System, Environment and Resources (TPESER), Institute of Tibetan Plateau Research, Chinese Academy of Sciences, Beijing 100101, China; State Key Laboratory of Tibetan Plateau Earth System, Environment and Resources (TPESER), Institute of Tibetan Plateau Research, Chinese Academy of Sciences, Beijing 100101, China; Nevada Bureau of Mines and Geology, Nevada Geosciences, University of Nevada, Reno, NV 89557, USA; State Key Laboratory of Tibetan Plateau Earth System, Environment and Resources (TPESER), Institute of Tibetan Plateau Research, Chinese Academy of Sciences, Beijing 100101, China; State Key Laboratory of Tibetan Plateau Earth System, Environment and Resources (TPESER), Institute of Tibetan Plateau Research, Chinese Academy of Sciences, Beijing 100101, China; State Key Laboratory of Tibetan Plateau Earth System, Environment and Resources (TPESER), Institute of Tibetan Plateau Research, Chinese Academy of Sciences, Beijing 100101, China

**Keywords:** paleomagnetism, geochemistry, Devonian bimodal volcanics, Northern Tibet, Paleo-Tethys

## Abstract

Rifting and drifting of the Qaidam–Kunlun continent in northern Tibet triggered the opening of the Paleo-Tethys Ocean, but its paleogeography remains debated, particularly the timing of ocean opening, which ranges from pre-Silurian to Devonian. This debate largely reflects the lack of reliable Devonian paleolatitude constraints. Here, we present new paleomagnetic, geochronological, and geochemical data from Devonian bimodal volcanics of the Qaidam–Kunlun. Primary remanent magnetization directions, supported by positive fold, reversal, and conglomerate tests, yield a paleolatitude of ∼25.6°S at ∼411 Ma. These results place Qaidam–Kunlun along the northern margin of Gondwana and demonstrate significant latitudinal separation from the contemporaneously northern North China during the Early Devonian. Geochemical characteristics of the volcanic indicate formation in an intraplate extensional regime. Integration with available Paleozoic paleomagnetic data suggests that Qaidam–Kunlun rifted from Indian Gondwana in the Early Devonian, and subsequently drifted northward during the Devonian, facilitating the opening of Paleo-Tethys.

## Teaser

This study constrains the paleolatitude and tectonic setting of the Lower Devonian bimodal volcanics from Qaidam–Kunlun, demonstrating that it rifted from Gondwana in the Early Devonian and subsequently drifted northward, facilitating the opening of the Paleo-Tethys Ocean.

## INTRODUCTION

Asia’s present-day tectonic architecture reflects the long-term dispersal of multiple continental terranes from the Southern Hemisphere and their northward migration into the Northern Hemisphere during the Phanerozoic. These northward accretions were principally driven by the successive Wilson-cycle evolutions of the Proto-, Paleo-, Meso-, and Neo-Tethys oceanic systems [1,2]. The modern Tibetan Plateau, composed of multiple Gondwanan-affinity terranes, preserves a rich record of these geological evolutions in major sutures such as the Indus–Yarlung, Bangong–Nujiang, Jinsha, and Qilian recording successive Tethyan closures [3,4]. Reconstructing the paleopositions and drift histories of these terranes is fundamental to understanding the tectonic growth of Asia, and the paleogeographic context of major biological and climatic events [5,6].

**Figure 1.**
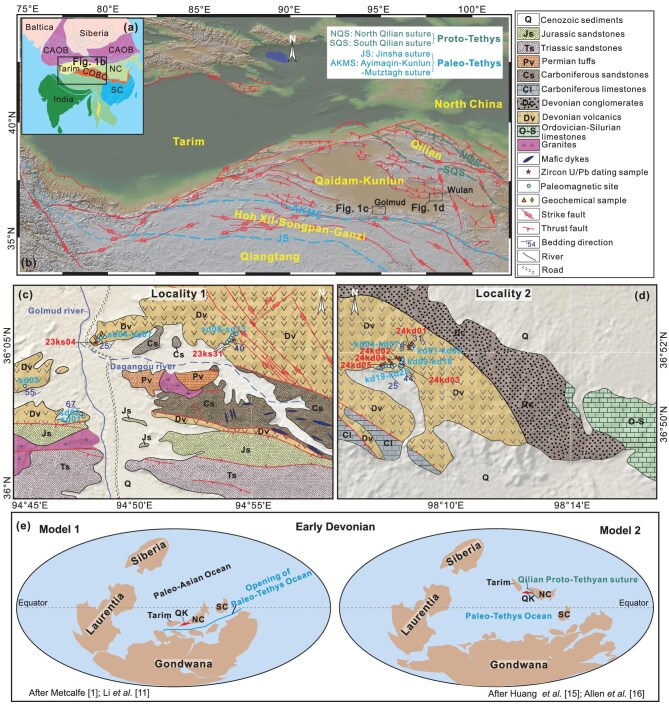
Tectonic and geological setting of the study area. (a) Tectonic framework of Eurasia showing major terranes and plates, modified after Huang *et al.* [15]. (b) Simplified tectonic map of the study region. (c) Geological map in Locality 1 showing sampling locations for paleomagnetic, geochronological, and geochemical analyses. (d) Geological map in Locality 2 with sample distribution. (e) Two prevailing models of paleogeographic reconstruction during the Early Devonian. COB, Central Orogenic Belt; CAOB, Central Asian Orogenic Belt; QK, Qaidam–Kunlun; NC, North China; SC, South China. Blue filled circles denote the locations of paleomagnetic sampling sites. Orange filled triangles and diamonds indicate the locations of geochemical basalt and dacite samples, respectively. Red filled five-pointed stars mark the locations of U-Pb dating samples.

The Paleozoic rifting and northward drift of the Qaidam–Kunlun continent (QK) in northern Tibet are closely linked to the opening of the Paleo-Tethys Ocean, with remnants preserved along the Jinsha and Ayimaqin–Kunlun–Mutztagh sutures (Fig. 1) [7–9]. However, the Silurian–Devonian paleogeographic position of the QK relative to Indian Gondwana and the North China craton remains debated [10], limiting our understanding of the timing and configuration of early Paleo-Tethys Ocean evolution. One model proposes that the QK and North China collided with southern Gondwana during the Silurian, closing the Proto-Tethys along the South and North Qilian sutures (Fig. 1e) [1,11]. The QK subsequently rifted away from Gondwana during the Early Devonian as a result of back-arc extension, initiating the opening of the Paleo-Tethys Ocean [7]. This interpretation is supported by Ordovician–Silurian ultrahigh-pressure (UHP) metamorphism in northern Tibet [12], a Silurian shift from subduction-related magmatism to A-type plutonism [10,13], and Early Devonian intraplate extensional magmatism [14]. However, the inferred Devonian northern paleolatitudes of North China, far removed from Gondwana, are difficult to reconcile with this model [15]. In contrast, an alternative model interprets these geological features as recording a Silurian collision between the QK and North China, positioned far north of Gondwana (Fig. 1e) [15,16]. This scenario requires that the Paleo-Tethys Ocean had already opened prior to the Silurian. These competing models therefore predict fundamentally different paleolatitudes for the QK during the Silurian–Devonian, either at southern latitudes along the northern margin of Indian Gondwana or at northern latitudes adjacent to North China.

Paleomagnetism provides a powerful tool for constraining paleolatitudes and testing these competing models [15,17]. However, reliable Devonian data from the QK remain scarce, as existing Paleozoic paleomagnetic data are largely restricted to the Carboniferous–Permian [9,18–20]. These data indicate predominantly northern paleolatitudes but offer limited insights on the timing and mechanism of the Paleo-Tethys opening. To address this gap, we present new zircon U-Pb geochronological, paleomagnetic, and geochemical data from Devonian volcanic rocks of the QK. Integrated with existing Paleozoic paleomagnetic constraints and coeval apparent polar wander paths (APWPs) from Indian Gondwana and North China, our results refine first-order Paleozoic kinematic evolution and clarify its role in the opening of the Paleo-Tethys Ocean.

## GEOLOGICAL SETTING AND SAMPLING

The QK is bounded to the south by Qiangtang and Hoh Xil–Songpan–Ganzi continents, separated by the Ayimaqin–Kunlun–Mutztagh and Jinsha ophiolite mélanges, which represent branches of Paleo-Tethys sutures (Fig. 1a). To the north, the QK is bounded by the North China and Qilian continents across the South and North Qilian ophiolite mélanges (Fig. 1a), interpreted as one of the remnants of the Proto-Tethys sutures [1,10]. The QK comprises the present-day Eastern Kunlun Range and Qaidam Basin, and consists of a Paleoproterozoic basement overlain by a Phanerozoic sedimentary cover. Regional geology features ∼460–420 Ma eclogite- and granulite-facies UHP metamorphic rocks that are exposed in the northern Qaidam Basin and Eastern Kunlun Range [11,21,22]. The continent also records two distinct phases of Phanerozoic magmatism: (1) ∼500–360 Ma granitoids, mafic dykes, and volcanic rocks [13]; and (2) ∼260–190 Ma arc-related magmatism [10].

**Figure 2.**
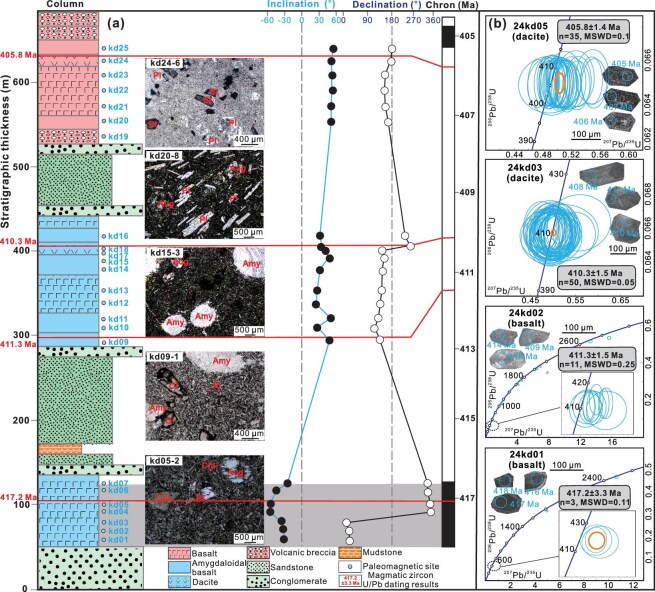
Stratigraphic column and analytical results in Locality 2. (a) Lithological description, sample distribution, petrographic observations, and magnetostratigraphic results. Our magnetostratigraphic results are compared with the global polarity [23]. Pl, plagioclase; Bt, biotite; Amy, amygdala; Ol, olive; Aug, pyroxene; Chl, chlorite. (b) Zircon U-Pb geochronological data. Black and white filled circles represent the inclinations and declinations, respectively.

We sampled the Devonian Maoniushan Formation at two localities: Locality 1 in the Eastern Kunlun Range (36.1°N, 94.9°E) and Locality 2 in the Qaidam Basin (36.9°N, 98.2°E). At Locality 1, 13 paleomagnetic sites (sd01–sd13) and two zircon U-Pb samples (23ks04 and 23ks31) were collected from tuffs, basalts, and dacites. At Locality 2, a ∼600-m-thick volcanic-sedimentary sequence yielded 25 paleomagnetic sites (kd01–kd25) and five zircon U-Pb samples (24kd01–24kd05). In total, 36 volcanic sites were collected for paleolatitude analysis, including three tuff sites (sd01–03), 11 dacite sites (sd04–sd07, sd09–sd12, kd17–kd18, and kd24), and 22 basalt sites. In addition, one volcanic breccia site (kd19) was sampled for a conglomerate test. At Locality 2, the succession comprises two sedimentary units (conglomerate, sandstone, and mudstone) and three volcanic units (amygdaloidal basalt, dacite, and volcanic breccia), each comprising multiple layers (Fig. 2a and Fig. S3). Eleven representative volcanic samples across distinct layers were analyzed for whole-rock geochemistry, including two dacites from Locality 1 and nine basalts from Locality 2 (Fig. 1).

## RESULTS

### Zircon U-Pb dating

Cathodoluminescence images and Th/U ratios reveal a magmatic origin for measured zircons (see Fig. 2b and Table S1). In Locality 1, dacite samples 23ks04 and 23ks31 yield ²⁰⁶Pb/²³⁸U weighted mean ages of 411 ± 0.6 Ma (*n* = 51, MSWD = 0.06) and 412 ± 0.6 Ma (*n* = 44, MSWD = 0.5), respectively (Fig. S2), indicating an Early Devonian eruption. In Locality 2, samples 24kd01 through 24kd05 were collected in stratigraphic order from base to top. Zircons from basaltic samples 24kd01 and 24kd02 display dispersed age distributions, comprising seven and 38 analyses, respectively. The youngest populations yield ²⁰⁶Pb/²³⁸U weighted mean ages of 417 ± 3.3 Ma (*n* = 3, MSWD = 0.11) for 24kd01 and 411 ± 1.5 Ma (*n* = 11, MSWD = 0.25) for 24kd02. Older inherited zircons in both samples span a similar range from 440 Ma to 2900 Ma. In contrast, dacite samples 24kd03 and 24kd05 each display a single dominant zircon population, with ²⁰⁶Pb/²³⁸U weighted mean ages of 410 ± 1.5 Ma (*n* = 50, MSWD = 0.05) and 406 ± 1.4 Ma (*n* = 35, MSWD = 0.1), respectively. Basalt sample 24kd04, however, yielded only three largely sparse zircon results with ²⁰⁶Pb/²³⁸U ages of 402 Ma, 471 Ma, and 2518 Ma. Collectively, these results indicate that the sampled volcanic rocks erupted during the Early Devonian (∼417–406 Ma).

### Paleomagnetism

**Figure 3.**
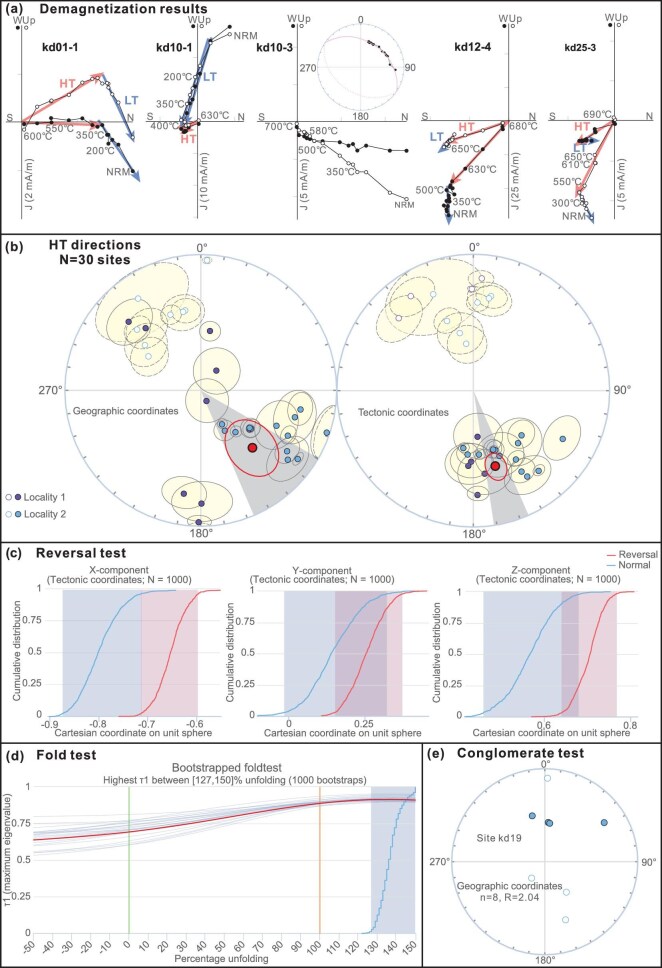
Paleomagnetic results from the Lower Devonian volcanic rocks. (a) Orthogonal vector diagrams of thermal demagnetization for representative specimens in geographic coordinates. Solid and open symbols represent projections on horizontal and vertical planes, respectively. (b) Equal-area projection of high-temperature component directions at the site level. (c) Bootstrap reversal test results. (d) Bootstrapped fold test results. (e) Conglomerate test results.

Rock magnetism shows that pseudo-single-domain (PSD) to single-domain hematites and PSD magnetites are primary remanence carriers (see Supplementary Materials). Petrographic observations confirm that these Fe-Ti oxides are primary magmatic phases, characterized by small grain sizes (<10 µm), low in Ti content (Table S2), and cubic or strip-like morphologies, which validates their reliability for paleomagnetic study (see Supplementary Materials). Natural remanent magnetization intensities range from 0.1 to 20 mA/m. Thermal demagnetization isolated two distinct magnetic components in ∼70% of specimens: a low-temperature (LT) component below 350–400°C and a high-temperature (HT) component between ∼400°C and 600–710°C (Fig. 3a). Only 20 specimens from sites kd09–kd11 and sd01–sd03 (∼12%) exhibit high temperature demagnetization trajectories that closely follow great circles (Fig. 3a and Fig. S9; Table S3).

The sample-mean LT component (D = 356.6°, I = 56.6°, α₉₅ = 3.6°, *n* = 114, *in situ* coordinates) aligns closely with the time-averaged recent field direction in the region (D = 0°, I = 55.6°), failing the fold test (Fig. S10). The HT component, decaying linearly toward the origin, is regarded as the characteristic remanent magnetization (ChRM). Of 36 volcanic sites, two (sd10–sd11) yielded unstable remanent directions, and four (kd17, kd18, sd08, and sd09) failed the 45° cutoff quality criteria control (Table 1). Among the remaining 30 sites, including 27 lava and 3 tuff sites, two polarities were identified: 19 sites with southeast/downward ChRM directions and 11 with northwest/upward directions. We combined these 30 sites to calculate overall locality-mean direction (Mean-1), following methods in the Materials and Methods. The overall locality-mean direction (geographic coordinates) is calculated as D = 138.0° ± 19.8°, I = 47.2° ± 19.8°, corresponding to a virtual geomagnetic pole (VGP) at 14.5°N, 312.0°E (A₉₅ = 16.6°, K = 3.5). After tilt correction, the Fisher mean direction is D = 163.2° ± 7.4°, I = 41.7° ± 9.1°, with the associated VGP located at 27.9°N, 292.1°E (A₉₅ = 6.8°, K = 16.0).

**Table 1.** Site-mean ChRM directions for the Devonian volcanic rocks of the Qaidam-Kunlun continent.

					Direction						Pole position				Expected direction at 36.1°N, 94.9°E			
Site	Rock	*n*/*n*_o_	Strike (°)	Dip (°)	D_g_ (°)	I_g_ (°)	D_s_ (°)	I_s_ (°)	*κ*	α_95_ (°)	φp_g_ (°E)	λp_g_ (°N)	φp_s_ (°E)	λp_s_ (°N)	D_g_ (°)	I_g_ (°)	D_s_ (°)	I_s_ (°)
**Locality 1 (36.1°N, 94.9°E)**																		
sd01	tuff	4/8	267	67	318.8	39.0	326.6	−18.4	105.6	12.2	354.9	51.7	316.2	34.7	318.8	39.0	326.6	−18.4
sd02	tuff	7/8	267	73	8.9	53.6	4.5	−18.7	114.5	6.1	197.3	82.5	268.7	44.1	8.9	53.6	4.5	−18.7
sd03	tuff	6/8	242	55	313.8	27.6	314.2	−25.1	25.8	13.7	349.7	43.6	325.0	24.5	313.8	27.6	314.2	−25.1
sd04	dacite	4/6	300	25	2.9	−4.7	359.6	−26.7	1742.8	2.2	270.2	51.5	275.4	39.8	2.9	−4.7	359.6	−26.7
sd05	dacite	5/6	300	25	191.0	20.3	185.1	43.8	46.8	11.3	333.7	50.0	269.7	28.1	191.0	20.3	185.1	43.8
sd06	dacite	5/6	300	25	179.5	17.8	171.9	38.5	27.7	14.8	275.6	44.8	283.8	31.7	179.5	17.8	171.9	38.5
sd07	dacite	4/6	300	25	180.7	3.0	177.5	24.6	318.5	5.2	273.8	52.4	278.1	40.9	180.7	3.0	177.5	24.6
sd08^†^	basalt	4/8	100	40	352.1	34.1	323.6	69.4	10.7	29.4	298.8	71.3	50.2	59.5	352.1	34.1	323.6	69.4
sd09^†^	dacite	8/9	100	40	18.3	48.7	89.7	84.4	21.9	12.1	202.5	73.4	108.6	35.4	18.3	48.7	89.7	84.4
sd12	dacite	6/8	100	40	39.4	74.9	174.0	62.3	23.7	14.0	126.2	54.6	279.3	10.1	39.4	74.9	174.0	62.3
sd13	basalt	5/8	100	40	150.4	82.5	183.4	44.0	35.3	13.1	102.8	23.0	271.4	28.0	150.4	82.5	183.4	44.0
**Locality 2 (36.9°N, 98.2°E)**																		
kd01	basalt	4/7	163	31	349.4	−39.8	10.9	−30.3	132.9	8.0	286.2	30.4	261.7	36.6	349.5	−39.9	11.0	−30.3
kd02	basalt	4/7	163	31	347.3	−40.7	10.0	−32.0	61.8	11.8	288.2	29.4	263.0	35.7	347.4	−40.8	10.1	−32.0
kd03	basalt	6/7	163	31	338.6	−39.8	3.0	−35.4	129.9	5.9	297.2	27.9	271.4	34.3	338.7	−39.8	3.1	−35.3
kd04	basalt	4/7	163	31	303.1	−52.4	351.9	−61.8	63.5	11.6	319.5	2.8	280.8	10.6	303.2	−52.5	352.1	−61.8
kd05	basalt	4/6	163	31	309.4	−46.1	347.2	−54.6	50.4	13.1	319.0	10.6	285.8	17.8	309.5	−46.2	347.3	−54.6
kd06	basalt	4/7	163	31	313.8	−35.4	339.6	−44.5	131.3	8.0	320.9	19.2	295.0	24.8	313.8	−35.5	339.7	−44.6
kd07	basalt	3/7	163	31	325.6	−19.2	338.4	−25.3	24.4	25.5	316.8	33.8	301.3	36.6	325.7	−19.3	338.5	−25.3
kd09	basalt	6/8	147	44	106.0	28.3	139.6	47.9	24.7	14.3	343.2	3.5	310.7	14.7	106.1	28.5	139.7	47.9
kd10	basalt	6/7	147	44	108.7	1.2	118.2	26.4	36.4	12.1	353.0	14.6	336.3	13.2	108.7	1.46	118.2	26.5
kd11	basalt	8/8	147	44	100.7	26.2	132.5	50.2	56.4	8.3	347.4	0.3	314.7	9.5	100.8	26.3	132.6	50.2
kd12	basalt	4/6	147	44	125.1	13.4	140.7	24.8	885.8	3.1	336.8	23.1	319.4	28.5	125.2	13.5	140.8	24.8
kd13	basalt	4/5	147	44	128.3	19.0	147.5	26.4	53.9	12.6	332.1	23.2	312.4	31.3	128.4	19.1	147.6	26.5
kd14	basalt	4/6	147	44	125.2	23.3	149.0	31.4	151.8	7.5	332.6	19.3	309.2	29.4	125.3	23.5	149.1	31.4
kd15	basalt	5/8	147	44	115.0	38.1	157.9	47.2	32.1	13.7	332.8	5.9	295.8	22.3	115.1	38.2	158.0	47.2
kd16	basalt	7/10	147	44	117.3	28.2	148.1	40.1	152.0	4.9	336.1	11.9	307.0	23.8	117.4	28.2	148.2	40.1
kd17^†^	dacite	5/8	147	44	271.6	76.2	246.4	34.2	169.1	5.9	63.3	32.6	213.9	6.7	271.6	76.2	246.5	34.1
kd18^†^	dacite	6/8	147	44	266.2	73.6	246.3	31.3	296.0	3.9	59.5	28.7	212.6	8.0	266.3	73.5	246.4	31.2
kd20	basalt	6/8	144	25	139.9	57.0	174.4	50.9	393.1	3.4	305.8	7.5	279.9	22.1	140	57.0	174.6	50.9
kd21	basalt	7/7	144	25	128.2	52.1	161.2	51.8	614.6	2.4	316.5	5.9	291.5	19.2	128.3	52.1	161.3	51.9
kd22	basalt	7/7	144	25	127.1	51.3	159.4	51.6	130.4	5.3	317.7	5.8	293.1	19.0	127.1	51.4	159.6	51.6
kd23	basalt	7/7	144	25	127.5	52.3	160.8	52.2	110.4	5.8	316.8	5.3	291.8	13.8	127.6	52.4	159.1	57.1
kd24	dacite	6/9	144	25	149.2	61.7	185.1	51.3	116.7	6.2	297.0	6.2	270.1	21.8	149.3	61.7	185.3	51.3
kd25	basalt	7/8	144	25	137.0	64.5	182.0	57.2	109.2	5.8	126.3	1.76	276.6	15.3	132.5	63.5	178.0	58.0
Mean-1	30 basalt + dacite + tuff sites (Locality 1 + 2)										312.0	14.5	292.1	27.9	138.0	47.2	163.2	41.7
Mean-2	27 basalt + dacite sites (Locality 1 + 2)										317.2	15.8	290.7	26.5	134.6	42.3	164.4	43.8

Note: Strike/dip, right hand strike/dip of beds; *n*/*n*_o_, number of samples used to calculate the site mean/number of demagnetized samples/sites; D_g_, I_g_, D_s_ and I_s_, declination and inclination in geographic and stratigraphic coordinates, respectively; *κ*, the best estimate of the precision parameter; α_95_, the radius of the mean direction at the 95% confidence level; λp_g_, φp_g_, λp_s_ and φp_s_, latitude and longitude of the corresponding virtual geomagnetic pole (VGP) in geographic and stratigraphic coordinates, respectively. The expected direction was calculated at the reference point (36.1°N, 94.9°E).

† sites are discarded from the final average due to the 45° cutoff.

The ∼600 m volcanic-sedimentary sequence at Locality 2 allows magnetostratigraphic correlation with Gradstein *et al.* [23]. The lower volcanic sites (kd01–kd07) display negative inclinations and northward declinations, whereas the overlying sedimentary site kd08 records a polarity reversal to positive inclination and southward declination (Fig. 2a). Higher sites (kd09–kd25) maintain this consistent polarity. Zircon U-Pb ages (417–406 Ma) indicate emplacement during an interval of normal polarity below and reversed polarities above (Fig. 2). Therefore, the negative and positive inclinations represent normal and reversed polarities, respectively, implying eruption in the Southern Hemisphere. In addition, the locality-mean ChRM inclination of our Devonian volcanics (41.7° ± 9.1°) is similar to that reported from Upper Permian redbeds in the Qaidam Basin (47°) [19] and tuffs in the Eastern Kunlun Range (46°) [9], which yield robust paleolatitudes of ∼27.4–28.2°N. The corresponding declinations, however, differ by ∼163–186°. Assuming no large-scale vertical-axis rotation of the QK between the Devonian and Permian, this near-antipodal relationship strongly suggests that Devonian and Permian magnetizations recorded an opposite latitude. Given the established northern latitude of the Permian reference pole [9,19], our 30 site-mean ChRMs are most consistently interpreted as southerly latitude.

### Geochemistry

**Figure 4.**
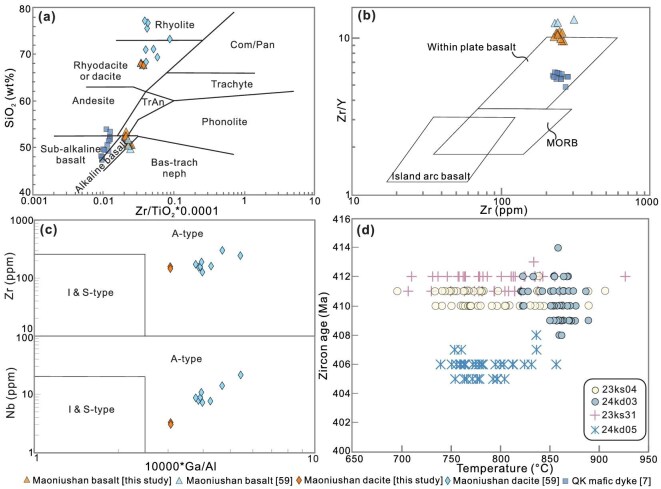
Whole-rock geochemistry of Lower Devonian volcanic rocks. (a) Zr/TiO_2_ vs SiO_2_ classification diagram. (b) Zr vs Zr/Y diagram distinguishing tectonic settings. (c) 10 000*Ga/Al vs Nb-Zr diagram distinguishing A- and I- & S-type patterns. (d) Ti-in-Zircon thermometer of our dacite samples. Data are from: Tables S1 and S4, Dong *et al.* [7], and Wang *et al.* [59].

All 11 samples analyzed exhibit low and consistent loss-on-ignition values (1.5–3.0 wt%), indicating overall limited post-eruptive alteration (Table S4). The Maoniushan basalts are characterized by low SiO_2_ (50–53 wt%) and moderate TiO_2_ (1.0–1.3 wt%), whereas the dacites have higher SiO_2_ (∼67 wt%) and low TiO_2_ (∼0.4 wt %). The basaltic samples display Mg# values of ∼56–71 and relatively high total alkalis (Na_2_O + K_2_O = 4.7%–5.8%). On the Zr/TiO_2_-SiO_2_ and Zr/Y-Zr discrimination diagrams, most basalts plot in the within-plate alkaline to subalkaline basalt fields (Fig. 4a and b), clearly distinguishing them from mid-ocean ridge basalts (MORBs) and island arc basalts (OIBs) and implying an intraplate affinity. Most of the dacites plot in the rhyodacite-dacite field and define a bimodal volcanic association within the basalts (Fig. 4a), a feature typical of continental extensional environments. The dacites dominantly plot in the A-type granite field on the Ga/Al-Zr and Ga/Al-Nb diagrams (Fig. 4c), implying high-temperature, dry magmatism associated with lithospheric extension. Consistent with this interpretation, Ti-in-zircon thermometry yields zircon crystallization temperatures of ∼750–900°C, with peaks around 800–880°C (Fig. 4d), indicating generation of hot, dry felsic magmas under elevated high geothermal gradients in the QK crust which are associated with lithospheric extension.

## DISCUSSION

### Early Devonian paleolatitude of the Qaidam–Kunlun continent

The mean ChRM directions from 30 volcanic sites (Mean-1) pass a fold test at a high confidence level (ξ_2_ = 11.4 before, and 8.4 after tilt correction; critical value ξ = 9 at the 99% confidence level) [24] and show statistically significant improvement in grouping after tilt correction (k_s_/k_g_ = 3.5; critical values: 1.86 at 99%, 1.55 at 95% confidence) [25]. We therefore interpret these directions as pre-folding in origin. This folding event is constrained as pre-Early Carboniferous by a regional angular unconformity, where Lower Carboniferous limestone overlies the folded Devonian Maoniushan Formation [26]. Furthermore, the Mean-1 ChRMs for normal polarity (D = 349.7°, I = –35.2°) and reversed polarity (D = 159.0°, I = 45.3°) sites pass three different reversal tests (Fig. 3d), including a class C test at the 95% confidence level (γ_caculated_ = 12.9 < γ_critical_ = 13.5) [27], and two bootstrap common mean direction tests (CMDT_caculated_ = 16.9 < CMDT_critical_ = 18.2 [28,29]). A conglomerate test on eight volcanic clasts from site kd19 is also positive at the 95% and 99% confidence levels (R_caculated_ = 2.04 < R_critical_ = 4.48 and 5.26, respectively) [30] (Fig. 3f). Collectively, these results support the interpretation of Mean-1 as a primary remanent magnetization.

However, a bootstrapped fold test of all 30 volcanic sites (Mean-1) indicates maximum directional clustering at 127%–150% unfolding [31] (Fig. 3e), and a progressive unfolding test of Enkin [32] is indeterminate (Fig. S12a). This pattern could result from primary dip or inclination shallowing of our three tuff sites. Our analysis indicates that the mean inclination of three tuff sites (I = 22.1) is ∼50% shallower than that of the 27 basalt and dacite sites (I = 43.6), likely reflecting post-depositional compaction of vast pyroclastic materials [33] (Fig. S3n–p). After applying a shallowing factor of ∼0.5 to the three tuff sites (sd01–sd03), two progressive unfolding tests of all 30 sites yield positive results with peak precision parameters at 110.3% ± 3.5% [34] and 110.4% ± 13.9% [32] (Fig. S12b). This suggests that the sampled volcanics have not experienced significant primary dip. Given the limited number of tuff sites (17 samples in 3 sites), an elongation/inclination (E/I) correction [35] could not be reliably applied. We therefore used only the 27 basalt and dacite sites to calculate the paleolatitude, excluding the three tuff sites. Their Fisher mean direction (Mean-2) in stratigraphic coordinates is D = 164.4° ± 7.6°, I = 43.8° ± 8.8°, corresponding to a VGP at 26.5°N, 290.7°E (A₉₅ = 6.9°, K = 17.5; Table 1). The A₉₅ value of 6.9° for the Mean-2 pole falls within the expected paleosecular variation (PSV) window (3.2–10.3°) for 27 poles [36]. This reasonable PSV, combined with the ∼11 Myr eruptive history capturing two polarities, indicates sufficient time averaging to support a reliable paleolatitude estimate. We therefore interpret the Mean-2 ChRMs as recording an Early Devonian geomagnetic signal, yielding a paleolatitude estimate of 25.6°S [95% confidence level: 19.3°S–33.2°S] for a reference point in the QK (36.1°N, 94.9°E).

### Implications for the evolution of the Paleo-Tethys Ocean

To refine paleogeographic constraints on the early evolution of the Paleo-Tethys Ocean, we compiled available Paleozoic paleomagnetic data from northern Tibet, including the QK and Qiangtang, and compared them with the APWPs of North China and Indian Gondwana (Table S5), calculating expected paleolatitudes at a reference point of 36.1°N, 94.9°E.

**Figure 5.**
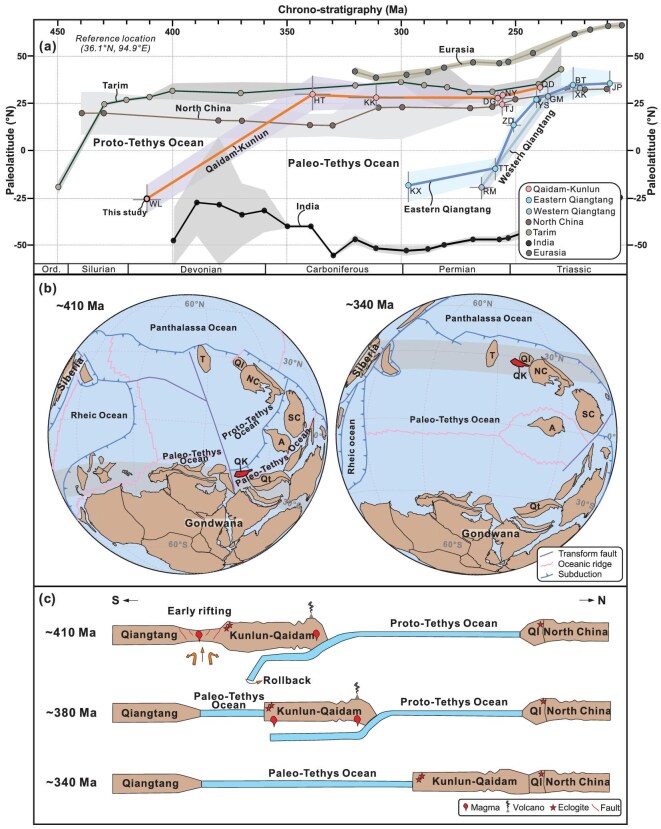
Synthesis of paleomagnetic data and paleogeographic reconstructions. (a) Paleolatitude evolution of northern Tibetan terranes based on compiled paleomagnetic data (Table S5). APWPs are from: Tarim and North China (after Huang *et al.* [15]), Eurasia and India (0–320 Ma after Vaes *et al.* [60] and 330–450 Ma after van Hinsbergen *et al.* [37]). (b) Paleogeographic reconstructions at ∼410 Ma and 340 Ma; 410 Ma using our new pole; 340 Ma using paleolatitude of the QK provided by Cao *et al.* [18]. (c) Paleozoic tectonic evolution model for northern Tibet. T, Tarim; Ql, Qilian; Qt, Qiangtang; A, Indochina.

Comparison of our new paleolatitude estimate for the QK (∼25.6°S at ∼410 Ma) with the coeval expected latitude of the Indian Gondwana (∼48.3 ± 18°S at 400 Ma [37]) suggests that the QK remained near northern Gondwana during the Early Devonian (Fig. 5a). In contrast, this latitude is far removed from the coeval positions of North China (∼15.7–19.6°N during 380–430 Ma [15]), effectively ruling out a Silurian collision between the QK and North China. The paleomagnetic results from the Lower Carboniferous limestones of the Huaitoutala Formation in the QK [18], which passed fold tests, yield negative inclinations and a mean declination of 298.6° in stratigraphic coordinates. Faunal evidence dates deposition to the Late Visean stage [38], a period dominated by reversed geomagnetic polarity [23], favoring a northern-hemisphere paleolatitude interpretation. E/I tests indicate no inclination shallowing [18], placing the QK at ∼29.4°N [21.8°N, 39.0°N] during the Early Carboniferous. Subsequent paleomagnetic results indicate that the QK occupied relatively stable paleolatitudes of ∼28–33°N from the Early Carboniferous through the Late Triassic [9,18–20,39] (Fig. 5a). Collectively, these data indicate that the QK occupied a Gondwana-adjacent position at ∼410 Ma and subsequently underwent rapid northward displacement of ∼6100 km between ∼410 and ∼340 Ma, corresponding to an average latitudinal drift rate of ∼8.7 cm/yr and comparable to fast Paleozoic plate motions [5,40] (Fig. 5a).

The geochemical characteristics of the Lower Devonian Maoniushan bimodal volcanic rocks indicate formation in a continental intraplate extensional regime at 410–390 Ma (Fig. 4). The widespread distribution of coeval A_2_-type granites and mafic dykes further supports regional lithospheric extension across the QK continent during the Early Devonian [7,14,41,42]. Within our paleomagnetic framework, this pervasive extensional magmatism is plausibly linked to the initial breakup of the QK from Indian Gondwana and the early opening of the Paleo-Tethys Ocean, rather than subduction-related arc magmatism or simple post-collisional collapse. This phase of lithospheric rifting may have been driven by back-arc extension related to ongoing subduction of the Qilian Proto-Tethys Ocean [7] and followed the Silurian collision between the QK and Gondwana [1,12]. Comparable switches from contractional to extensional tectonic regimes in ∼10–20 Myr are also documented in other orogenic systems, including in North China [43] and Norway [44]. Independent support for this interpretation is provided by mid-oceanic-type Jinsha–Ailaoshan ophiolites (∼383–341 Ma [45]) and contemporary pelagic rocks in the Changning–Menglian area [46]. Together, the paleomagnetic, petrographic, and geochemical evidence suggests that Early Devonian rifting of the QK from Indian Gondwana, followed by Middle–Late Devonian northward drifting toward North China, provide the kinematic framework for early Paleo-Tethys opening (Fig. 5). Progressive consumption of the Paleo-Tethys Ocean likely accompanied Late Paleozoic northward convergence of Qiangtang toward the QK (Fig. 5a) [47]. Arc-type magmatism in the QK indicates that northward subduction of the Paleo-Tethys Ocean beneath the QK was established by at least ∼270 Ma [10], with final closure occurring during the Late Triassic following collision between Qiangtang and the QK [9,47] (Fig. 5b).

This paleomagnetic framework also places several key Paleozoic geological events in northern Tibet into a refined spatial context. UHP metamorphism (∼460–420 Ma) and magmatism (∼460–400 Ma) in the Eastern Kunlun Range and Qaidam Basin occurred at low southern latitudes along the northern Gondwanan margin (Fig. 5c) and recorded a Silurian collision between the QK and Gondwana [10]. In contrast, contemporaneous UHP metamorphism (∼489–446 Ma [12,16]) and arc magmatism (∼502–415 Ma [10,13]) in the Qilian continent likely reflect Silurian collision between the Qilian and North China continents along the North Qilian suture at northern latitudes. The pronounced Early Devonian latitudinal separation between North China and the QK, combined with the cessation of arc activities across the QK and Qilian region during 360–270 Ma [48], and widespread Devonian/Carboniferous unconformities [10,49,50], suggests that the collision between the QK and North China occurred during the Middle–Late Devonian (Fig. 5c). This collision would have resulted in the closure of the Proto-Tethys Ocean along the South Qilian suture. Targeted paleomagnetic studies on Middle–Upper Devonian rocks from the Qaidam–Kunlun region are therefore critical for constraining its northward drift paths and refining the early evolution of the Paleo-Tethys Ocean.

## CONCLUSIONS

Northern Tibet preserves a critical archive of the Paleozoic evolution of the Tethys Ocean. However, the paleogeographic evolution remains debated due to the scarcity of paleomagnetic constraints. Our new findings can be summarized as follows: (1) Lower Devonian volcanic rocks from the Qaidam–Kunlun continent carry primary remanent magnetizations, as demonstrated by positive fold, reversal, and conglomerate tests; (2) paleomagnetic analyses indicate a southern-hemisphere latitude of 25.6°S [19.3°S–33.2°S] for Qaidam–Kunlun during ∼417–406 Ma, consistent with a Gondwana-adjacent configuration and incompatible with contemporaneous northern latitudes of the North China craton; (3) geochemical characteristics of the volcanic rocks indicate formation in a continental rift setting; and (4) available Paleozoic paleomagnetic data suggest Qaidam–Kunlun experienced a rapid northward displacement of ∼6100 km between ∼410 and ∼340 Ma; (5) taken together, these results suggest that Qaidam–Kunlun had separated from Gondwana by the Early Devonian and subsequently migrated northwards before the Early Carboniferous, a process closely associated with the early rifting of the Paleo-Tethys Ocean.

## MATERIALS AND METHODS

### Zircon U-Pb dating

Zircon crystals were separated from volcanic samples using standard crushing and heavy liquid techniques. We examined their internal structures with cathodoluminescence images and performed zircon dating by Laser-ablation-multicollector inductively coupled plasma mass spectrometry (LA-ICP-MS) at the State Key Laboratory of Tibetan Plateau Earth System, Environment and Resources (TPESER), Institute of Tibetan Plateau Research, Chinese Academy of Sciences. The crystallization age of each sample was inferred from the mean age of its youngest zircon group. All ages were calculated using Isoplot software [51] and the results are presented in Fig. S2 and Table S1.

### Paleomagnetism

All basalt, dacite, and tuff paleomagnetic sites were sampled over several meters of lateral outcrop and throughout the thickness of each distinctive flow or layer, using a portable gasoline-powered drill. Samples were oriented in the field using both sun and magnetic compasses. Each site consists of at least six core samples, and each core was cut into one or two standard specimens using a diamond saw. All specimens underwent thermal demagnetization, using an ASC TD-48 oven in TPESER. The treatment followed a stepwise protocol with temperature intervals of 30–50°C below 500°C and 10–20°C above 500°C. After each step, the remanent magnetization was measured with a 2G-755R superconducting rock magnetometer in a magnetically shielded room at TPESER. LT and HT directions were determined via principal component analysis (PCA) or a combination method of great circle analysis and directional observations using the PMGSC 4.2 software. We filtered the characteristic magnetizations and site mean directions passing the 45°-cutoff. Given that the two sampling localities are ∼320 km apart, we transferred the site-mean directions from Locality 2 to the corresponding expected directions at Locality 1. This was done by first calculating the virtual geomagnetic poles (VGPs) from the site-mean directions and then converting them to the expected directions at the reference locality. Inclination-upwards directions were inverted to downwards inclinations for consistency, while their declinations were transferred by ±180°. The locality-mean directions and VGPs with their uncertainties were calculated using the online paleomagnetic analysis platform Paleomagnetism.org [52]. The sample-level paleomagnetic directions are listed in Table S3.

### Geochemistry

Major element analyses were conducted using an X-ray fluorescence spectrometer (XRF-2400) with an Axios Max X Minerals spectrometer, with 0.1%–1% analytical uncertainties. Trace element abundances were determined by a Thermo-Scientific iCAP Qc inductively coupled plasma-mass spectrometer, with better than 5% analytical uncertainties for most elements. All geochemistry tests were carried out at Beijing GeoAnalysis Co. Ltd., Beijing, China. The Zr/TiO₂ vs SiO_2_ classification diagram is based on Winchester and Floyd [53]. The Zr vs Zr/Y diagram distinguishing tectonic settings is based on Pearce and Norry [54]. Ga/Al vs Nb-Zr diagrams distinguishing A-type and I & S-type are based on Whalen *et al*. [55]. The Ti-in-Zircon thermometer is based on Ferry and Watson [56]. Normalization values for chondrite-normalized REE patterns and primitive mantle-normalized multi-element plots are from Sun and McDonough [57]. Modern MORB/OIB fields are based on Zindler and Hart [58]. The geochemical data are provided in Table S4.

## Supplementary Material

nwag131_Supplemental_File
