# Supplementary material for: Devonian northward drift of the Qaidam–Kunlun continent constrains the early evolution of the Paleo-Tethys Ocean
Source: Natl Sci Rev. 2026 Mar 3;13(7):nwag131. doi: 10.1093/nsr/nwag131 (PMC13114873; doi:10.1093/nsr/nwag131)
Supplement: nwag131_Supplemental_File [file nwag131_supplemental_file.docx]

Supplementary Materials for

**Devonian northward drift of the Qaidam–Kunlun continent constrains the early evolution of the Paleo-Tethys Ocean**

Peiping Song^1*^, Lin Ding^1^, Chen Wu^1^, Andrew V. Zuza^2^, Liyun Zhang^1^, Yahui Yue^1^ and Jing Xie ^1^

^1^State Key Laboratory of Tibetan Plateau Earth System, Environment and Resources (TPESER), Institute of Tibetan Plateau Research, Chinese Academy of Sciences, Beijing 100101, China.

^2^Nevada Bureau of Mines and Geology, Nevada Geosciences, University of Nevada, Reno, Nevada 89557, USA.

*To whom correspondence should be addressed. E-mail: songpp@itpcas.ac.cn

**Contents of this file**

Text S1–S2

Figures S1–S12

Tables S1–S6

References

**Supplementary TEXT**

**Petrographic Analysis and Results**

Photomicrographs of representative samples (*e.g.*, kd05-2, kd09-1, kd10-3) were obtained using transmitted polarizing light to examine mineral textures. Subsequent analysis of Fe-bearing minerals was conducted with a JEOL JXA‐8230 electron microprobe analyzer in back‐scattering electron imaging mode (BSE) at the State Key Laboratory of Tibetan Plateau Earth System, Environment and Resources (TPESER), Institute of Tibetan Plateau Research, Chinese Academy of Sciences.

Phenocrysts are primarily pyroxene (~10%), with plagioclase observed in only one sample (kd20-8). These are randomly distributed in a fine-grained groundmass of microcrystalline plagioclase (50–60%), basaltic glass (25–35%), and Fe–Ti oxides (10–15%). A few basalts show minor chloritization of pyroxene phenocrysts (Fig. S3). The dacites also display porphyritic textures, with phenocrysts of plagioclase (10–20%) and biotite (10–15%) in a volcanic glass matrix. Overall, the rocks are relatively fresh, showing only minor alteration. Overall, the rocks are relatively fresh. BSE images reveal anhedral to subeuhedral magmatic minerals, including magnetite (typically cubic) and hematite (typically strip-shaped), dispersed between plagioclase laths (Fig. S4). These oxides are predominantly less than 10 μm in size and exhibit low Ti:Fe ratios (<0.1, Table S2). These mineralogical and geochemical characteristics indicate a primary magmatic origin for the Fe-Ti oxides, confirming the reliability of these samples for paleomagnetic study.

**Rock Magnetic** **Treatment and Results**

Magnetic susceptibility versus temperature curves (χ–T) were measured using an MFK1-FA Kappabridge coupled with a CS-4 high-temperature furnace at TPESRE. Hysteresis parameters, isothermal remanent magnetization (IRM), and back‐field demagnetization of saturation IRM data, and first-order reversal curve (FORC) data were acquired using a Lakeshore 8600 Vibrating Sample Magnetometer (VSM) at TPESER. IRM component analyses are based on Maxbauer *et al*. [1] using Max UnMix (https://shinyapps.carleton.edu/max-unmix). FORC data were processed using FORCinel 3.08 [2] and the VARIFORC algorithm [3], with signal-to-noise confidence limits following the method of Heslop and Roberts [4]. The FORC measurement parameters and FORC diagram smoothing parameters are provided in Table S6.

Rock magnetic analyses of six representative samples from the Devonian volcanic rocks indicate that their χ–T curves typically exhibit a peak around 500–550 °C, followed by a sharp drop between 580 and 600 °C and a gradual decay from 620 to 700 °C (Fig. S5), suggesting the presence of both magnetite and hematite. For most specimens, the cooling curves show a significant increase in susceptibility, likely due to thermal alteration of Fe-bearing phases, such as clay dehydration or exsolution of Fe-Ti-rich minerals from original iron oxides during heating. Isothermal remanent magnetization (IRM) acquisition curves do not saturate fully even at 1 T and generally show high coercivity of remanence (Bcr) values ranging from 138 to 617 mT, indicating a predominance of high-coercivity magnetic carriers. A subset of samples exhibits a rapid increase in remanence, reaching over 80% saturation above 300 mT (Figs. S6a–b), suggesting a relatively higher proportion of low-coercivity grains. In contrast, most samples display a gradual increase in remanence, attaining less than 30% saturation below 100 mT (Figs. S6c–f), which reflects a limited contribution from low-coercivity minerals. IRM component analysis reveals two dominant coercivity fractions centered approximately at 210–290 mT and 330–480 mT (Figs. S6g-l), interpreted as corresponding to hematite populations with different grain sizes or domain states. Hysteresis loops are predominantly “pot-bellied” or “wasp-waisted” in shape, with high coercivity (Bc) values generally exceeding 100 mT (Fig. S7), consistent with a magnetic mineral assemblage dominated by hematite and subordinate magnetite. The FORC diagrams show a weakly interacting, mixed-domain state and mineralogy. The FORC distribution is interpreted as a mixture of predominantly pseudo-single-domain (PSD) and minor multi-domain (MD) magnetites, combined with PSD to single-domain (SD) hematite [5] (Fig. S8). Together, these results indicate that the remanent magnetization in these volcanic rocks is mainly carried by PSD to SD hematite and PSD magnetite grains.

**FIGURE**

**
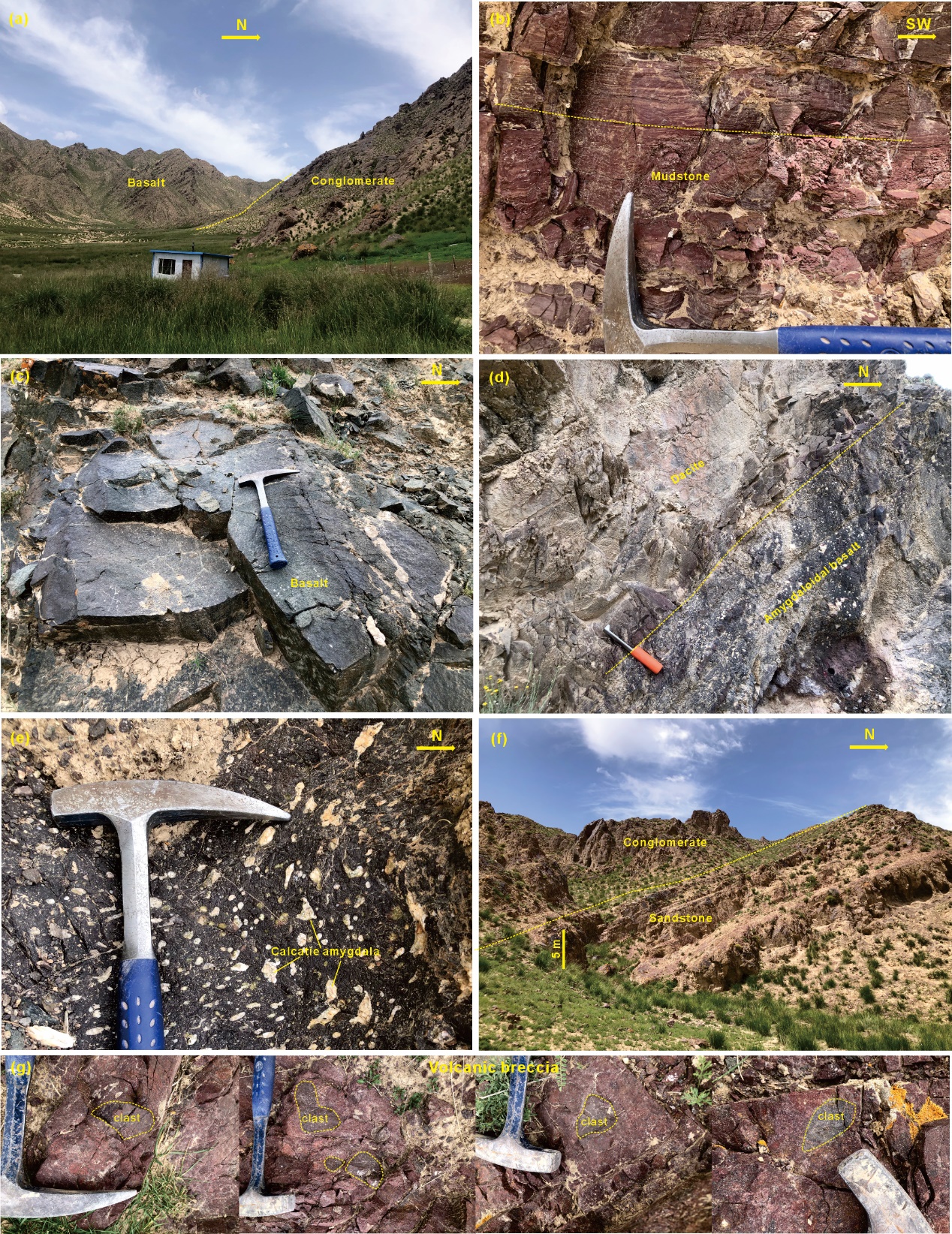
**

**Figure S1.** Field observations of Devonian volcanic strata near the Wulan area.

**
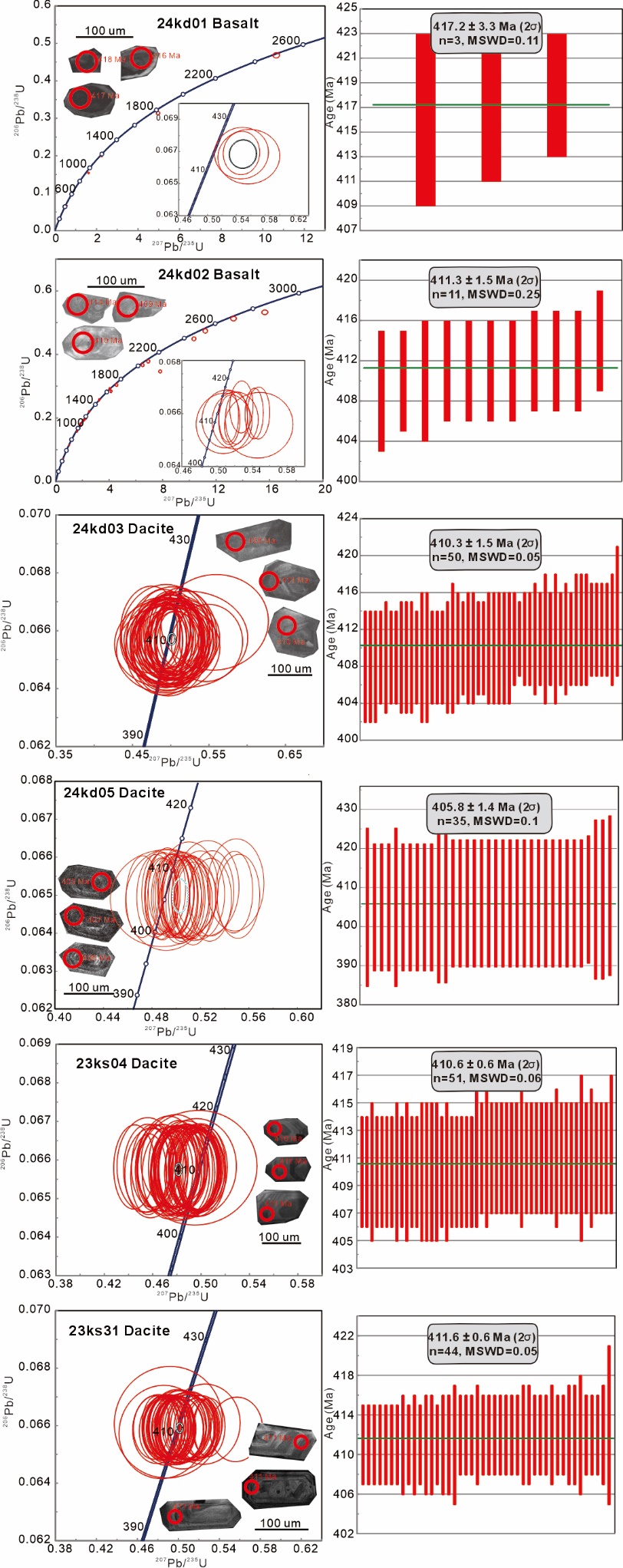
**

**Figure S2.** Zircon U-Pb data with representative CL images.

**
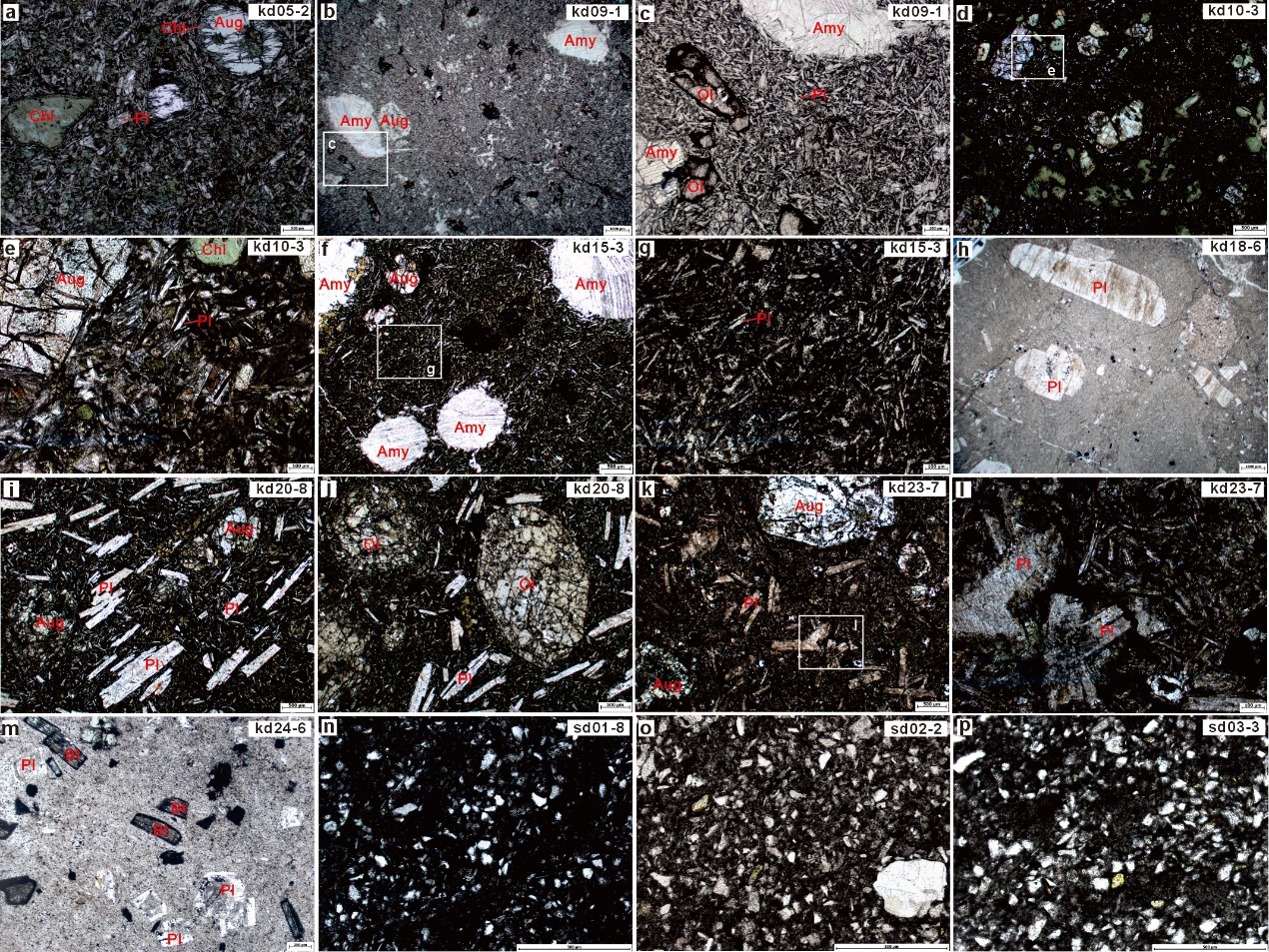
**

**Figure S3.** Photomicrographs of some representative samples from Devonian volcanic rocks under the plane-polarized light. Pl-plagioclase; Bt-biotite; Amy-amygdala; Ol-olive; Aug-pyroxene; Chl-chlorite.

**
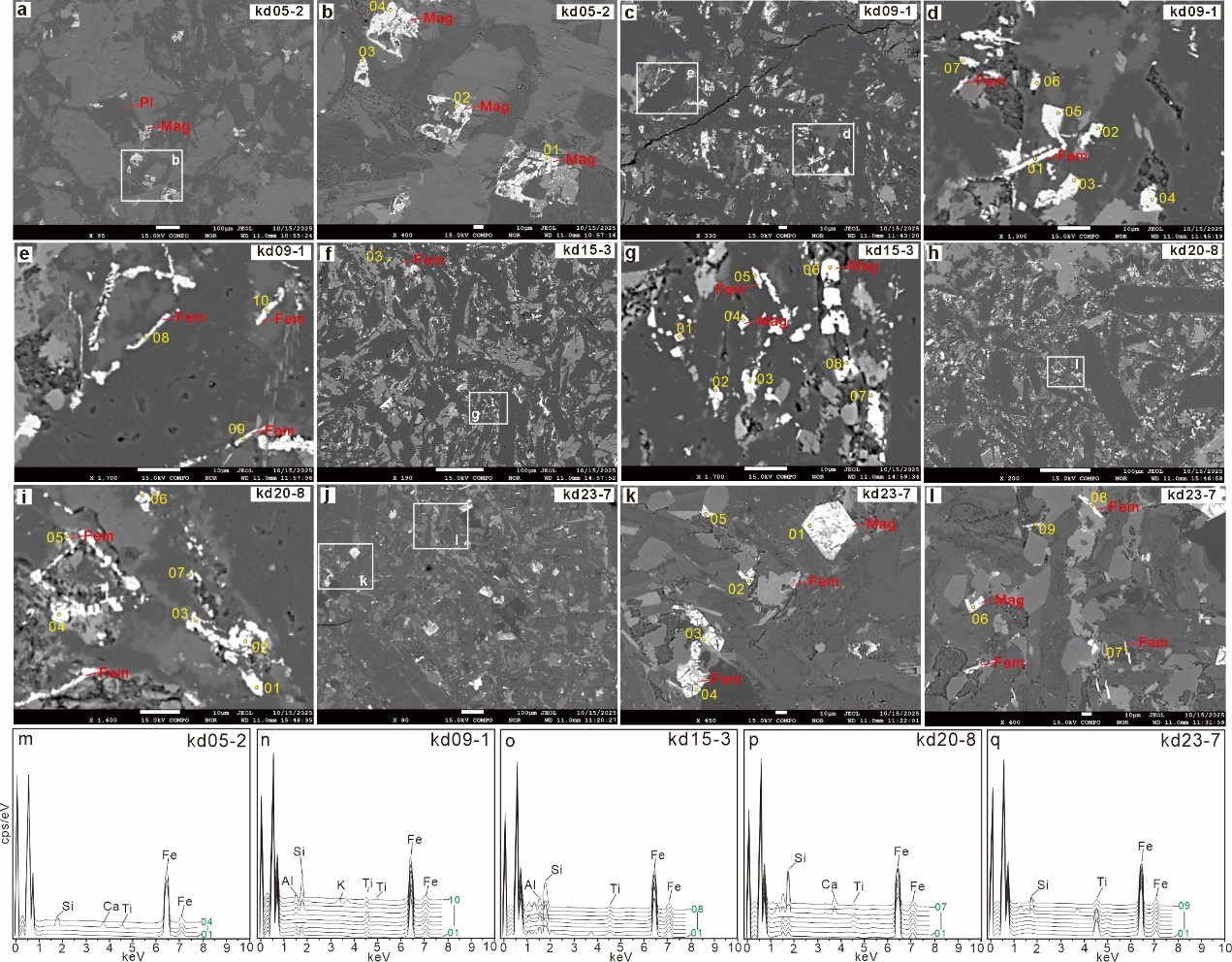
Figure S4.** Backscatter electron images and energy dispersive spectroscopy (EDS) analyses of representative specimens from the Devonian lavas. The yellow solid circles in panels (a–l) indicate spots analyzed using EDS analyzer (m–q). Abbreviations: Mag-magnetite, Hem-hematite, Pl-plagioclase.

**
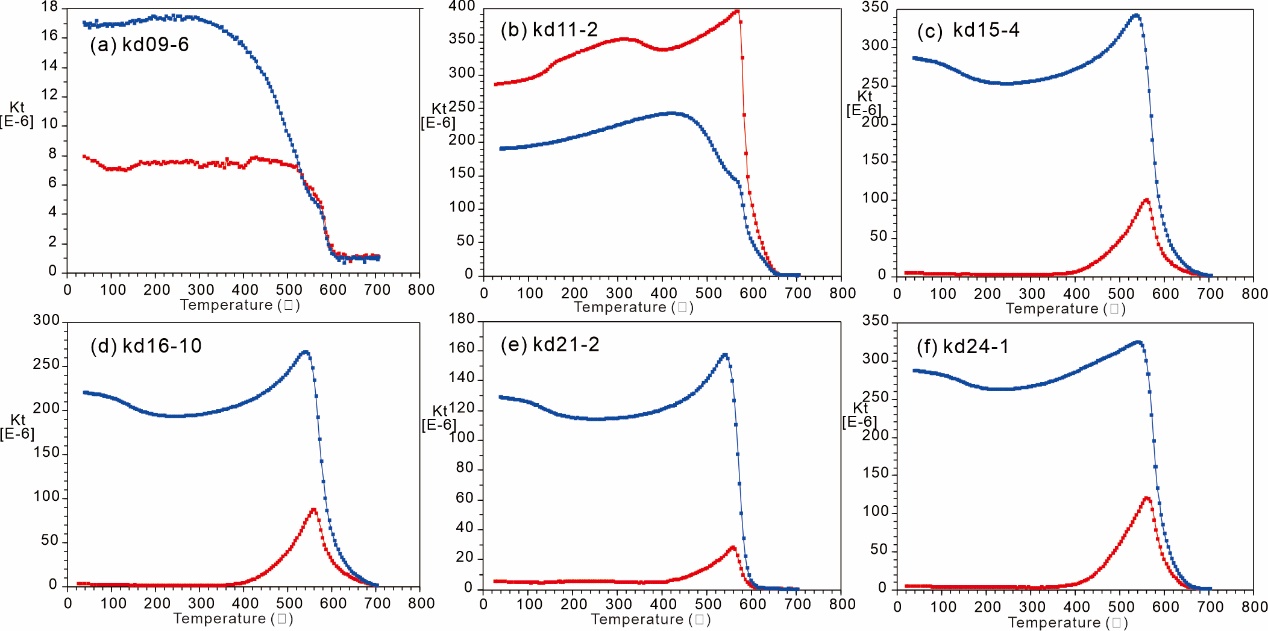
**

**Figure S5.** Low‐field magnetic susceptibility as a function of temperature for some representative specimens from the Devonian volcanics in argon environment (a–f): Red line is the heating curve; blue line is the cooling curve.

**
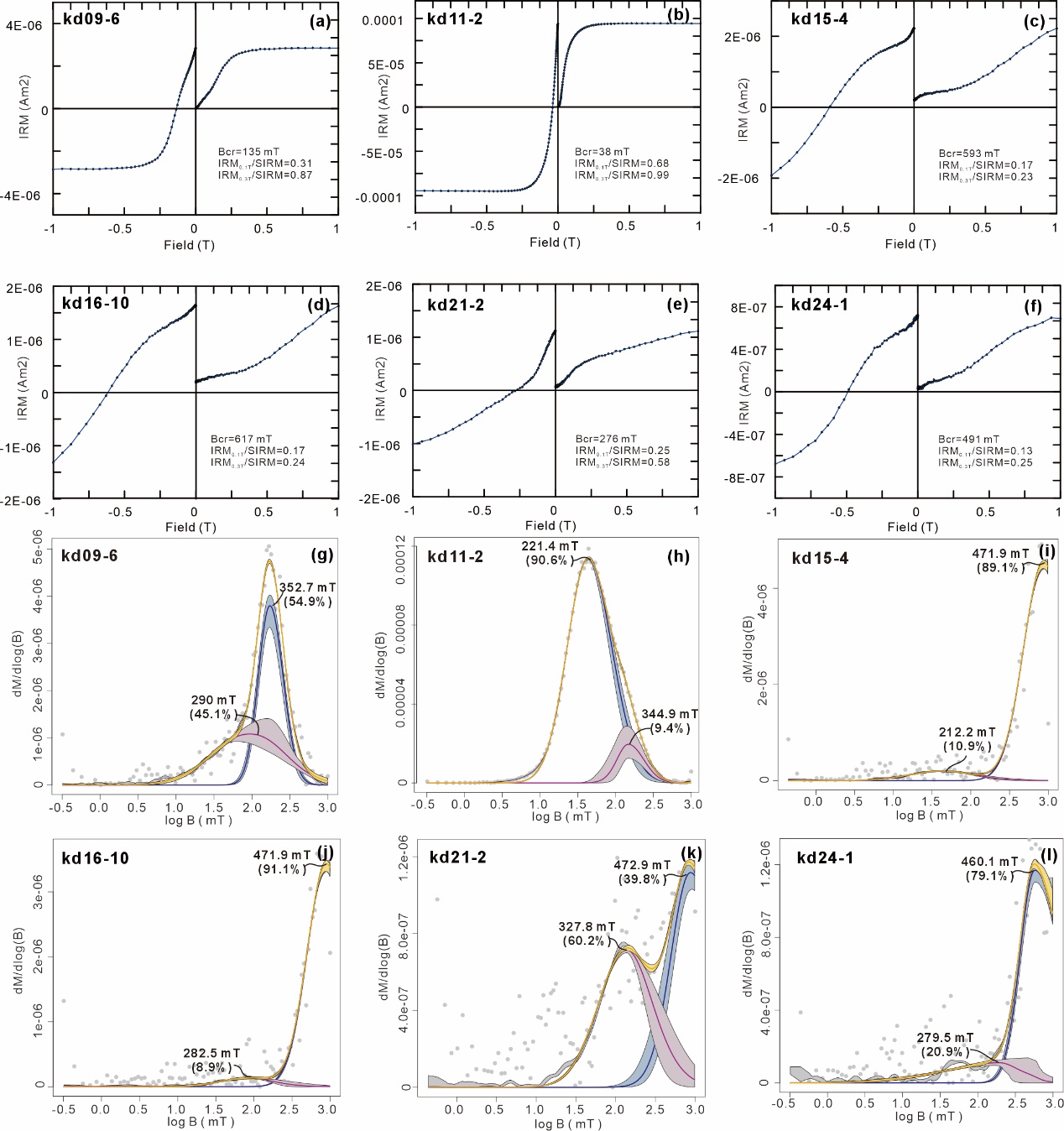
**

**Figure S6.** Acquisition of isothermal remanent magnetization (IRM), back‐field demagnetization of saturation IRM (a–f), and IRM component analysis (g–l) for some representative specimens from the Devonian volcanics.

**
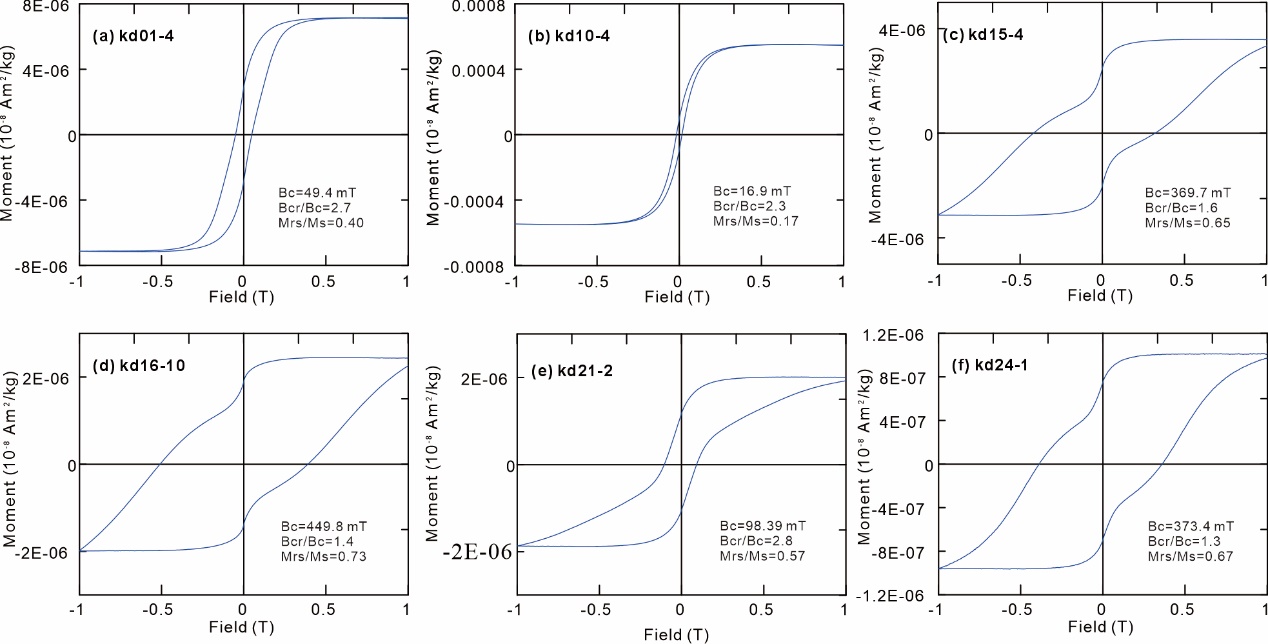
**

**Figure S7.** Hysteresis loops for some representative specimens from the Devonian volcanics.

**
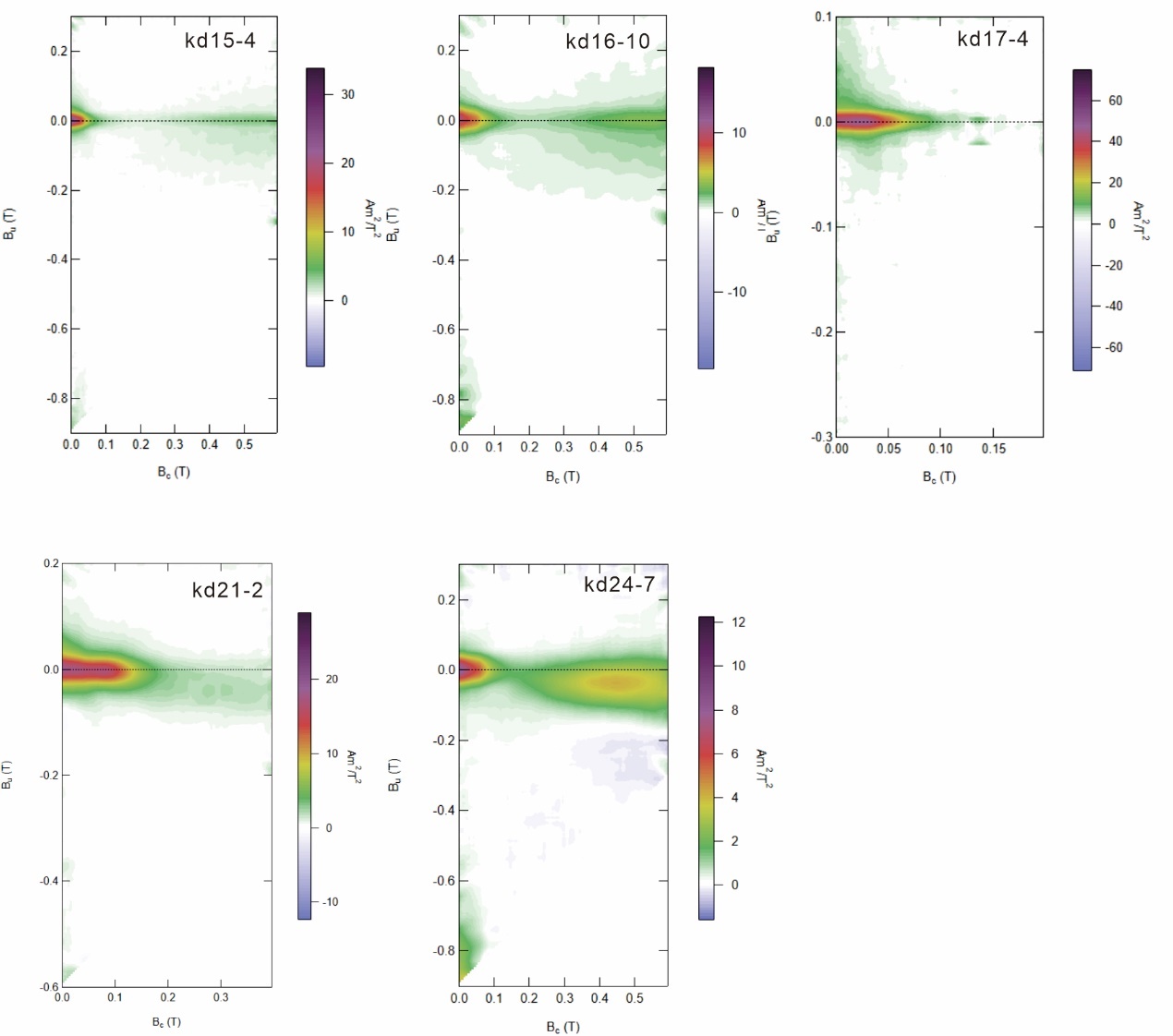
**

**Figure S8.** First-order reversal curve (FORC) distributions.

**
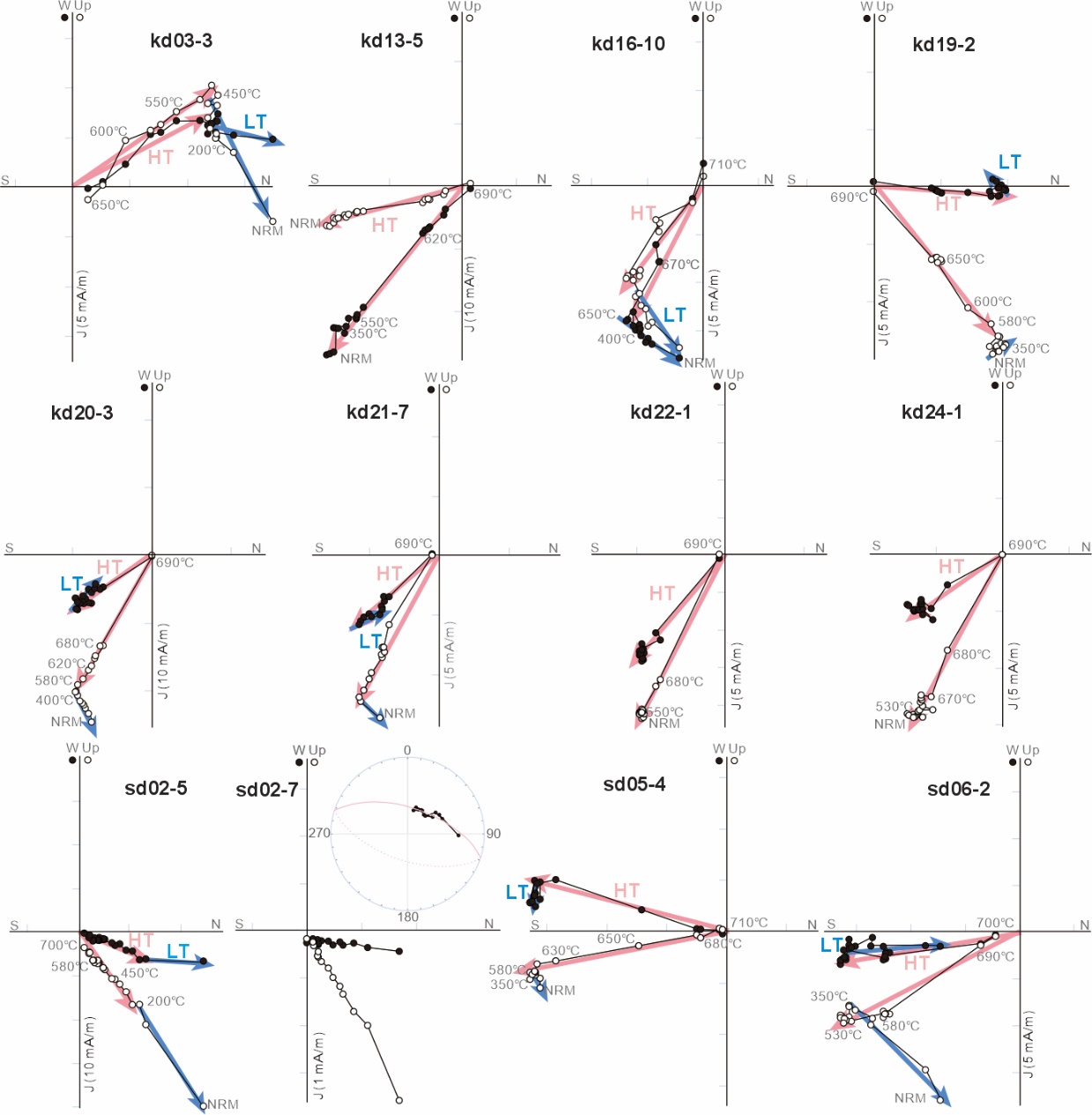
**

**Figure S9.** Orthogonal vector diagrams of thermal demagnetization behaviors of typical volcanic specimens in *in situ* coordinates, showing low-temperature (LT) and high-temperature (HT) components.

**
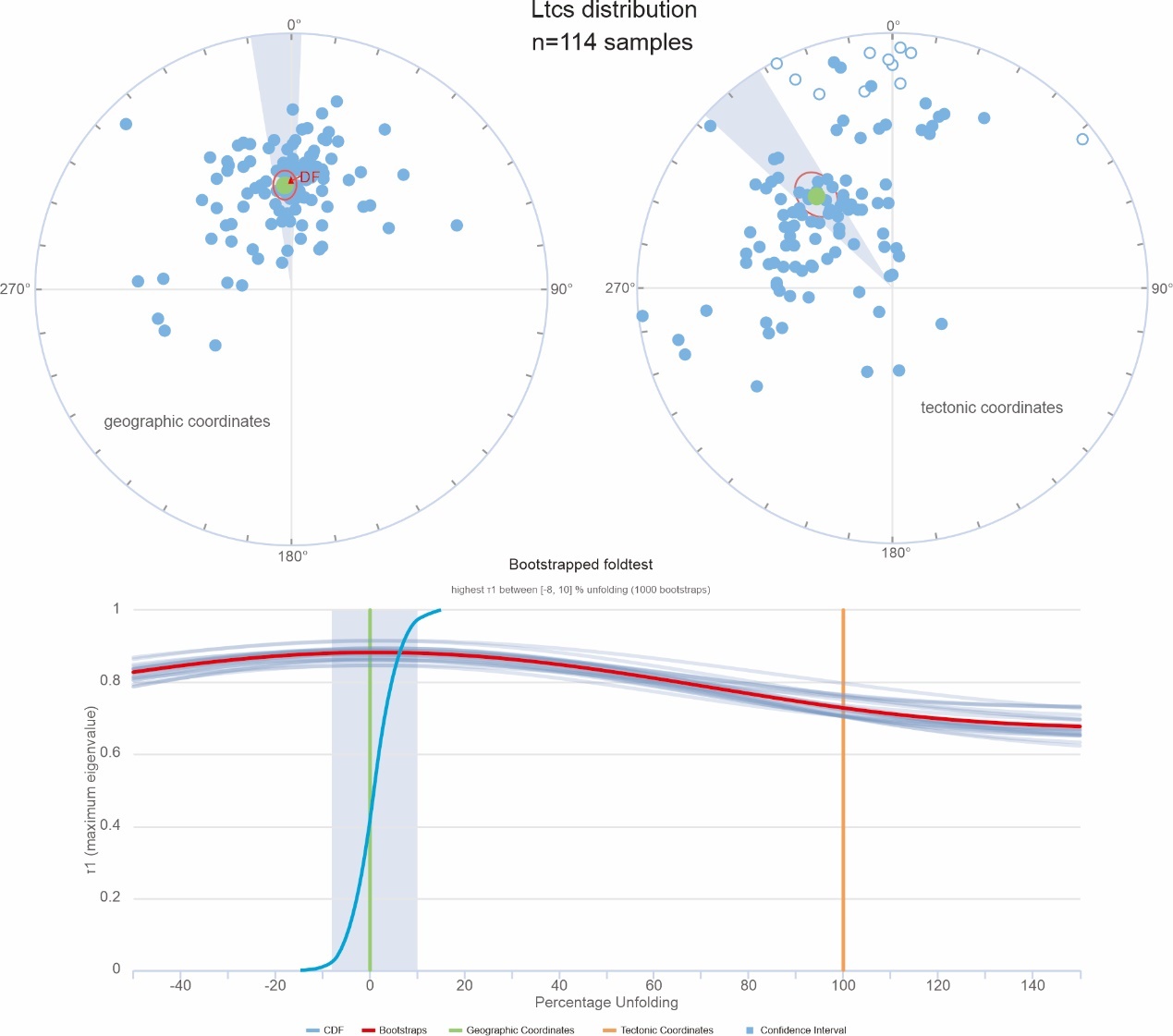
**

**Figure S10.** Equal-area projections of the paleomagnetic sample-mean LT direction and results of bootstrapped fold test [6].


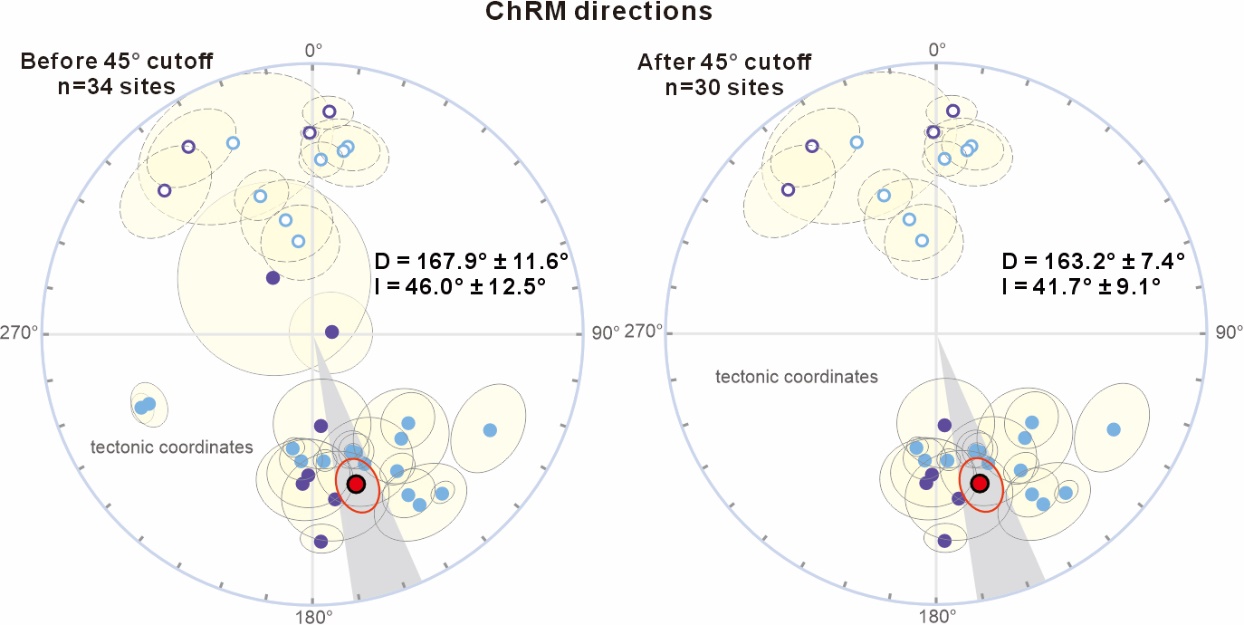


**Figure S11.** Equal-area projections of the paleomagnetic site-mean HT directions before and after 45° cutoff in the tectonic coordinates.


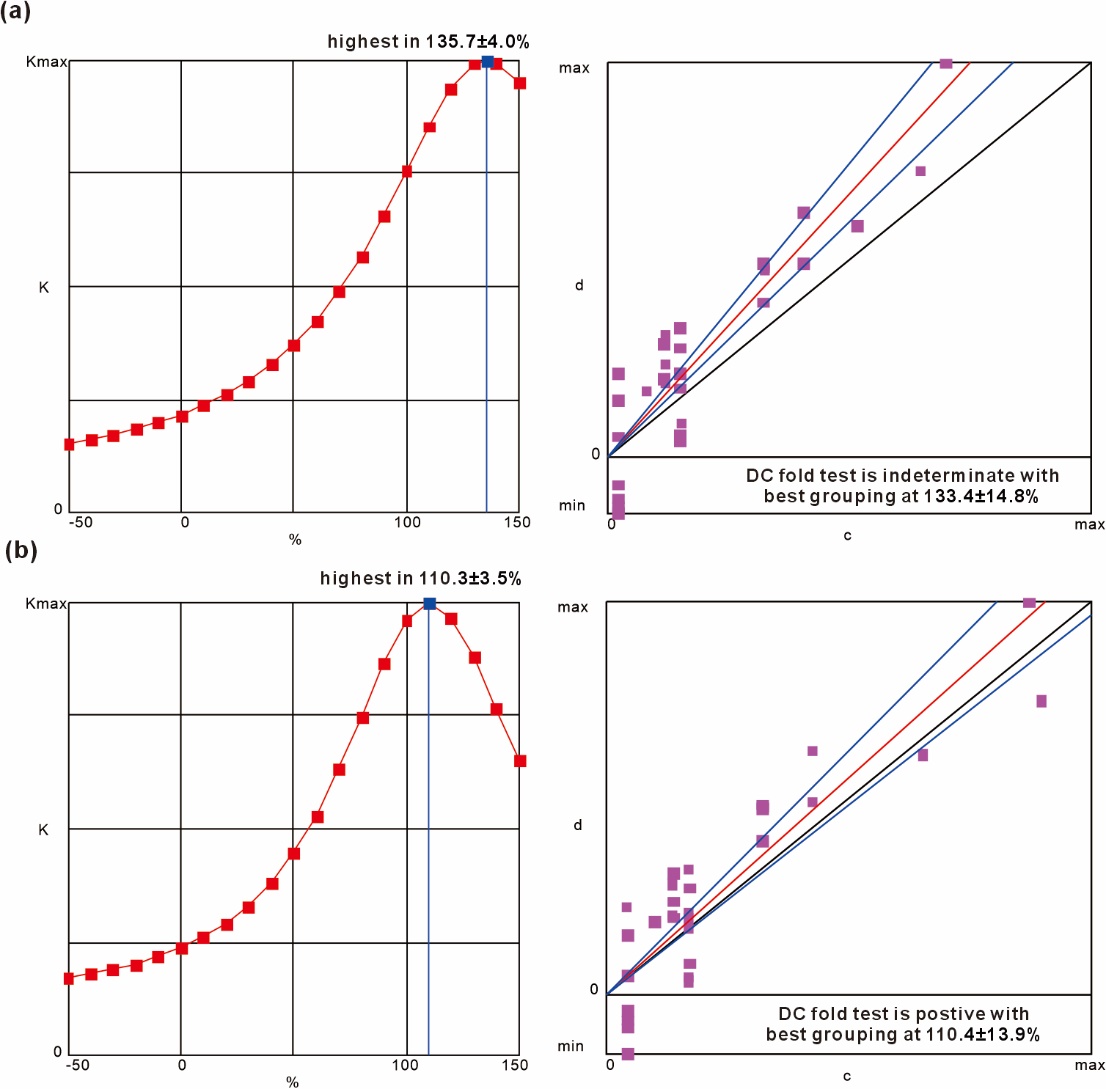


**Figure S12.** Progressive unfolding tests: left-Watson and Enkin [6]; right-Enkin [7]. (a) All 30 sites (basalt, dacite, and tuff), without shallowing correction for tuff sites. (b) All 30 basalt, dacite, and tuff sites, after applying an inclination shallowing factor of ~0.5 to three tuff sites.

**Table S1 Zircon U-Pb isotopic data and Ti-thermometer for Devonian lavas from the Qadaim-Kunlun continent. *Radiogenic Pb, corrected for common Pb.**

| Spot ID | Th/U | Isotopic ratio | | | | | | Apparent ages±1σ (Ma) | | | | | | Ti-in-zircon  thermometer | |
| --- | --- | --- | --- | --- | --- | --- | --- | --- | --- | --- | --- | --- | --- | --- | --- |
|  |  | ^207^Pb*/  ^235^U | ±1σ | ^206^Pb*/  ^238^U | ±1σ | ^208^Pb*/  ^232^Th | ±1σ | ^207^Pb*/  ^235^U | ±1σ | ^206^Pb*/  ^238^U | ±1σ | ^208^Pb*/  ^232^Th | ±1σ | Ti  (ppm) | T (°C) |
| **24kd01 (GPS: 36°51'56.47"N, 98°8'48.97"E)** | | | | | | | | | | | | | | | |
| 24kd01-10 | 0.65 | 4.9634 | 0.0663 | 0.3138 | 0.0039 | 0.1106 | 0.0010 | 1813 | 11 | 1760 | 19 | 2120 | 18 | / | / |
| 24kd01-11 | 1.24 | 0.5571 | 0.0283 | 0.0667 | 0.0011 | 0.0220 | 0.0004 | 450 | 18 | 416 | 7 | 439 | 9 | / | / |
| 24kd01-13 | 0.78 | 0.5471 | 0.0246 | 0.0669 | 0.0011 | 0.0262 | 0.0005 | 443 | 16 | 417 | 6 | 523 | 10 | / | / |
| 24kd01-2 | 0.13 | 1.6155 | 0.0308 | 0.1544 | 0.0019 | 0.0464 | 0.0007 | 976 | 12 | 926 | 11 | 917 | 13 | / | / |
| 24kd01-5 | 0.03 | 2.2475 | 0.0298 | 0.2001 | 0.0024 | 0.0597 | 0.0010 | 1196 | 9 | 1176 | 13 | 1172 | 19 | / | / |
| 24kd01-8 | 0.67 | 10.6508 | 0.1316 | 0.4683 | 0.0057 | 0.1247 | 0.0011 | 2493 | 11 | 2476 | 25 | 2375 | 19 | / | / |
| 24kd01-9 | 0.71 | 0.5470 | 0.0167 | 0.0669 | 0.0009 | 0.0285 | 0.0004 | 443 | 11 | 418 | 5 | 568 | 7 | / | / |
| **24kd02 (GPS: 36°51'39.51"N, 98°8'41.82"E)** | | | | | | | | | | | | | | | |
| 24kd02-03 | 0.91 | 0.4994 | 0.0111 | 0.0660 | 0.0008 | 0.0208 | 0.0002 | 411 | 8 | 412 | 5 | 416 | 4 | / | / |
| 24kd02-04 | 0.29 | 11.1984 | 0.1361 | 0.4738 | 0.0055 | 0.1299 | 0.0013 | 2540 | 11 | 2500 | 24 | 2469 | 23 | / | / |
| 24kd02-06 | 1.14 | 4.5600 | 0.0785 | 0.3040 | 0.0038 | 0.0867 | 0.0009 | 1742 | 14 | 1711 | 19 | 1681 | 17 | / | / |
| 24kd02-07 | 0.29 | 13.3107 | 0.1710 | 0.5128 | 0.0061 | 0.1374 | 0.0017 | 2702 | 12 | 2669 | 26 | 2603 | 30 | / | / |
| 24kd02-08 | 0.21 | 2.1031 | 0.0378 | 0.1925 | 0.0023 | 0.0576 | 0.0007 | 1150 | 12 | 1135 | 12 | 1132 | 13 | / | / |
| 24kd02-10 | 0.55 | 0.5103 | 0.0224 | 0.0658 | 0.0009 | 0.0205 | 0.0002 | 419 | 15 | 411 | 5 | 410 | 4 | / | / |
| 24kd02-12 | 0.25 | 0.5140 | 0.0098 | 0.0658 | 0.0008 | 0.0204 | 0.0002 | 421 | 7 | 411 | 5 | 409 | 4 | / | / |
| 24kd02-13 | 1.41 | 6.9345 | 0.0869 | 0.3781 | 0.0044 | 0.1041 | 0.0008 | 2103 | 11 | 2067 | 21 | 2001 | 14 | / | / |
| 24kd02-14 | 0.67 | 0.5207 | 0.0215 | 0.0657 | 0.0009 | 0.0204 | 0.0002 | 426 | 14 | 410 | 5 | 408 | 4 | / | / |
| 24kd02-15 | 0.30 | 1.6466 | 0.0209 | 0.1668 | 0.0019 | 0.0475 | 0.0004 | 988 | 8 | 994 | 11 | 938 | 8 | / | / |
| 24kd02-17 | 0.56 | 1.7783 | 0.0424 | 0.1703 | 0.0021 | 0.0512 | 0.0006 | 1038 | 15 | 1014 | 12 | 1010 | 11 | / | / |
| 24kd02-18 | 0.62 | 0.5220 | 0.0189 | 0.0622 | 0.0008 | 0.0192 | 0.0002 | 426 | 13 | 389 | 5 | 384 | 4 | / | / |
| 24kd02-19 | 0.78 | 0.5474 | 0.0104 | 0.0660 | 0.0008 | 0.0207 | 0.0002 | 443 | 7 | 412 | 5 | 414 | 4 | / | / |
| 24kd02-20 | 0.94 | 0.5147 | 0.0233 | 0.0641 | 0.0009 | 0.0199 | 0.0002 | 422 | 16 | 400 | 5 | 397 | 4 | / | / |
| 24kd02-21 | 0.22 | 1.1515 | 0.0159 | 0.1272 | 0.0015 | 0.0404 | 0.0004 | 778 | 7 | 772 | 8 | 801 | 8 | / | / |
| 24kd02-22 | 0.45 | 1.8516 | 0.0251 | 0.1820 | 0.0021 | 0.0458 | 0.0004 | 1064 | 9 | 1078 | 12 | 905 | 8 | / | / |
| 24kd02-25 | 2.20 | 0.8363 | 0.0134 | 0.0952 | 0.0011 | 0.0279 | 0.0002 | 617 | 7 | 586 | 7 | 557 | 4 | / | / |
| 24kd02-26 | 0.63 | 2.0255 | 0.0321 | 0.1912 | 0.0023 | 0.0563 | 0.0006 | 1124 | 11 | 1128 | 12 | 1107 | 11 | / | / |
| 24kd02-27 | 0.58 | 1.6098 | 0.0332 | 0.1579 | 0.0020 | 0.0438 | 0.0006 | 974 | 13 | 945 | 11 | 866 | 11 | / | / |
| 24kd02-28 | 0.05 | 10.3477 | 0.1192 | 0.4490 | 0.0051 | 0.1246 | 0.0015 | 2466 | 11 | 2391 | 23 | 2373 | 26 | / | / |
| 24kd02-29 | 0.25 | 0.5492 | 0.0205 | 0.0712 | 0.0010 | 0.0232 | 0.0007 | 444 | 13 | 443 | 6 | 464 | 13 | / | / |
| 24kd02-30 | 0.79 | 0.5424 | 0.0085 | 0.0660 | 0.0008 | 0.0220 | 0.0002 | 440 | 6 | 412 | 5 | 440 | 4 | / | / |
| 24kd02-34 | 0.41 | 15.6504 | 0.1831 | 0.5326 | 0.0062 | 0.1392 | 0.0011 | 2856 | 11 | 2753 | 26 | 2635 | 19 | / | / |
| 24kd02-35 | 0.61 | 2.1829 | 0.0282 | 0.1956 | 0.0023 | 0.0628 | 0.0005 | 1176 | 9 | 1152 | 12 | 1231 | 10 | / | / |
| 24kd02-36 | 1.08 | 4.1473 | 0.0572 | 0.2837 | 0.0034 | 0.0798 | 0.0007 | 1664 | 11 | 1610 | 17 | 1552 | 13 | / | / |
| 24kd02-38 | 0.38 | 0.5278 | 0.0109 | 0.0663 | 0.0008 | 0.0205 | 0.0003 | 430 | 7 | 414 | 5 | 410 | 5 | / | / |
| 24kd02-39 | 0.89 | 0.5133 | 0.0092 | 0.0659 | 0.0008 | 0.0197 | 0.0002 | 421 | 6 | 411 | 5 | 394 | 4 | / | / |
| 24kd02-40 | 0.55 | 2.2714 | 0.0286 | 0.1989 | 0.0023 | 0.0555 | 0.0004 | 1203 | 9 | 1169 | 12 | 1091 | 8 | / | / |
| 24kd02-44 | 0.44 | 0.5080 | 0.0259 | 0.0656 | 0.0009 | 0.0204 | 0.0002 | 417 | 17 | 410 | 6 | 409 | 4 | / | / |
| 24kd02-45 | 0.40 | 1.0160 | 0.0146 | 0.1137 | 0.0013 | 0.0337 | 0.0003 | 712 | 7 | 694 | 8 | 670 | 6 | / | / |
| 24kd02-46 | 0.23 | 1.8677 | 0.0261 | 0.1802 | 0.0021 | 0.0457 | 0.0005 | 1070 | 9 | 1068 | 12 | 903 | 10 | / | / |
| 24kd02-47 | 0.45 | 2.5055 | 0.0367 | 0.2203 | 0.0026 | 0.0580 | 0.0006 | 1274 | 11 | 1283 | 14 | 1139 | 11 | / | / |
| 24kd02-48 | 0.17 | 3.2549 | 0.0391 | 0.2568 | 0.0030 | 0.0605 | 0.0005 | 1470 | 9 | 1474 | 15 | 1188 | 10 | / | / |
| 24kd02-49 | 0.83 | 0.5475 | 0.0319 | 0.0656 | 0.0010 | 0.0202 | 0.0002 | 443 | 21 | 409 | 6 | 405 | 5 | / | / |
| 24kd02-50 | 0.65 | 1.2884 | 0.0243 | 0.1316 | 0.0016 | 0.0386 | 0.0004 | 841 | 11 | 797 | 9 | 765 | 8 | / | / |
| 24kd02-51 | 0.85 | 0.5221 | 0.0102 | 0.0658 | 0.0008 | 0.0201 | 0.0002 | 427 | 7 | 411 | 5 | 402 | 4 | / | / |
| 24kd02-52 | 0.89 | 0.37987 | 0.03164 | 0.04753 | 0.00097 | 0.01556 | 0.00052 | 2047 | 11 | 2014 | 20 | 1318 | 11 | / | / |
| 24kd02-53 | 0.74 | 8.964 | 1.34728 | 0.41517 | 0.01548 | 0.11592 | 0.01106 | 1643 | 11 | 1650 | 17 | 1438 | 11 | / | / |
| **24kd03 (GPS: 36°51'37.02"N, 98°8'39.65"E)** | | | | | | | | | | | | | | | |
| 24kd03-01 | 0.80 | 0.4994 | 0.0272 | 0.0658 | 0.0009 | 0.0212 | 0.0004 | 411 | 18 | 411 | 5 | 425 | 9 | 15.39 | 864 |
| 24kd03-02 | 1.23 | 0.5015 | 0.0130 | 0.0655 | 0.0008 | 0.0213 | 0.0003 | 413 | 9 | 409 | 5 | 425 | 5 | 13.65 | 850 |
| 24kd03-03 | 0.81 | 0.5198 | 0.0533 | 0.0657 | 0.0011 | 0.0200 | 0.0007 | 425 | 36 | 410 | 7 | 400 | 14 | 14.94 | 860 |
| 24kd03-04 | 0.82 | 0.4882 | 0.0355 | 0.0657 | 0.0010 | 0.0213 | 0.0005 | 404 | 24 | 410 | 6 | 426 | 10 | 14.58 | 857 |
| 24kd03-05 | 0.86 | 0.5311 | 0.0134 | 0.0658 | 0.0007 | 0.0217 | 0.0003 | 433 | 9 | 411 | 4 | 433 | 5 | 10.72 | 823 |
| 24kd03-06 | 0.81 | 0.4935 | 0.0441 | 0.0656 | 0.0010 | 0.0205 | 0.0006 | 407 | 30 | 409 | 6 | 409 | 12 | 16.33 | 871 |
| 24kd03-07 | 0.84 | 0.5057 | 0.0262 | 0.0655 | 0.0009 | 0.0221 | 0.0004 | 416 | 18 | 409 | 5 | 441 | 8 | 14.91 | 860 |
| 24kd03-08 | 0.82 | 0.4995 | 0.0343 | 0.0656 | 0.0009 | 0.0236 | 0.0005 | 411 | 23 | 409 | 5 | 470 | 9 | 14.72 | 859 |
| 24kd03-09 | 0.88 | 0.4972 | 0.0120 | 0.0656 | 0.0008 | 0.0222 | 0.0003 | 410 | 8 | 410 | 5 | 444 | 6 | 10.79 | 824 |
| 24kd03-10 | 0.82 | 0.4985 | 0.0377 | 0.0656 | 0.0010 | 0.0218 | 0.0005 | 411 | 26 | 409 | 6 | 436 | 10 | 14.62 | 858 |
| 24kd03-12 | 0.90 | 0.4967 | 0.0295 | 0.0653 | 0.0009 | 0.0215 | 0.0004 | 409 | 20 | 408 | 6 | 429 | 9 | 15.01 | 861 |
| 24kd03-13 | 0.83 | 0.4949 | 0.0296 | 0.0655 | 0.0010 | 0.0210 | 0.0005 | 408 | 20 | 409 | 6 | 420 | 10 | 14.5 | 857 |
| 24kd03-14 | 0.86 | 0.4993 | 0.0346 | 0.0655 | 0.0009 | 0.0213 | 0.0004 | 411 | 23 | 409 | 6 | 427 | 9 | 15.35 | 863 |
| 24kd03-15 | 0.87 | 0.5070 | 0.0375 | 0.0657 | 0.0009 | 0.0222 | 0.0004 | 416 | 25 | 410 | 6 | 443 | 9 | 16.24 | 870 |
| 24kd03-16 | 0.91 | 0.4988 | 0.0256 | 0.0659 | 0.0009 | 0.0220 | 0.0004 | 411 | 17 | 411 | 5 | 439 | 8 | 14.05 | 853 |
| 24kd03-17 | 0.85 | 0.4967 | 0.0446 | 0.0654 | 0.0010 | 0.0221 | 0.0005 | 409 | 30 | 408 | 6 | 442 | 11 | 15.26 | 863 |
| 24kd03-18 | 0.97 | 0.4992 | 0.0288 | 0.0657 | 0.0009 | 0.0211 | 0.0004 | 411 | 19 | 410 | 6 | 423 | 8 | 17.12 | 876 |
| 24kd03-19 | 0.82 | 0.5012 | 0.0148 | 0.0656 | 0.0008 | 0.0216 | 0.0003 | 413 | 10 | 410 | 5 | 433 | 6 | 10.68 | 823 |
| 24kd03-20 | 1.07 | 0.5474 | 0.0307 | 0.0656 | 0.0009 | 0.0215 | 0.0004 | 443 | 20 | 409 | 5 | 430 | 8 | 19.01 | 889 |
| 24kd03-21 | 0.95 | 0.4986 | 0.0278 | 0.0658 | 0.0008 | 0.0223 | 0.0004 | 411 | 19 | 411 | 5 | 446 | 7 | 13.8 | 851 |
| 24kd03-22 | 0.88 | 0.5062 | 0.0173 | 0.0657 | 0.0008 | 0.0228 | 0.0003 | 416 | 12 | 410 | 5 | 455 | 6 | 13.8 | 851 |
| 24kd03-23 | 0.83 | 0.5013 | 0.0261 | 0.0659 | 0.0009 | 0.0208 | 0.0004 | 413 | 18 | 411 | 6 | 416 | 9 | 14.87 | 860 |
| 24kd03-24 | 0.86 | 0.4930 | 0.0127 | 0.0659 | 0.0008 | 0.0226 | 0.0003 | 407 | 9 | 411 | 5 | 451 | 6 | 10.75 | 823 |
| 24kd03-25 | 0.84 | 0.4922 | 0.0374 | 0.0659 | 0.0011 | 0.0239 | 0.0007 | 406 | 25 | 411 | 7 | 477 | 13 | 16.12 | 869 |
| 24kd03-26 | 0.90 | 0.4986 | 0.0303 | 0.0657 | 0.0009 | 0.0213 | 0.0004 | 411 | 21 | 410 | 6 | 427 | 9 | 14.93 | 860 |
| 24kd03-27 | 0.86 | 0.4924 | 0.0338 | 0.0654 | 0.0011 | 0.0220 | 0.0006 | 407 | 23 | 409 | 7 | 439 | 11 | 17.04 | 876 |
| 24kd03-28 | 0.88 | 0.4999 | 0.0138 | 0.0658 | 0.0008 | 0.0224 | 0.0003 | 412 | 9 | 411 | 5 | 448 | 6 | 11.24 | 828 |
| 24kd03-29 | 0.82 | 0.5013 | 0.0389 | 0.0658 | 0.0011 | 0.0212 | 0.0006 | 413 | 26 | 411 | 7 | 424 | 13 | 18.6 | 886 |
| 24kd03-30 | 0.81 | 0.5059 | 0.0131 | 0.0659 | 0.0008 | 0.0225 | 0.0003 | 416 | 9 | 411 | 5 | 449 | 6 | 10.51 | 821 |
| 24kd03-32 | 0.89 | 0.4983 | 0.0337 | 0.0655 | 0.0012 | 0.0252 | 0.0007 | 411 | 23 | 409 | 7 | 504 | 13 | 16.19 | 870 |
| 24kd03-33 | 0.87 | 0.4735 | 0.0258 | 0.0660 | 0.0010 | 0.0219 | 0.0005 | 394 | 18 | 412 | 6 | 438 | 10 | 14.18 | 854 |
| 24kd03-34 | 1.16 | 0.4963 | 0.0162 | 0.0656 | 0.0008 | 0.0243 | 0.0003 | 409 | 11 | 409 | 5 | 484 | 6 | 15.13 | 862 |
| 24kd03-35 | 0.98 | 0.5068 | 0.0269 | 0.0656 | 0.0009 | 0.0239 | 0.0004 | 416 | 18 | 409 | 5 | 477 | 8 | 16.21 | 870 |
| 24kd03-36 | 0.77 | 0.5126 | 0.0286 | 0.0657 | 0.0009 | 0.0238 | 0.0005 | 420 | 19 | 410 | 6 | 475 | 9 | 13.99 | 853 |
| 24kd03-37 | 1.15 | 0.5052 | 0.0206 | 0.0654 | 0.0008 | 0.0240 | 0.0003 | 415 | 14 | 409 | 5 | 479 | 7 | 14.56 | 857 |
| 24kd03-38 | 0.89 | 0.5173 | 0.0246 | 0.0660 | 0.0009 | 0.0246 | 0.0005 | 423 | 16 | 412 | 6 | 492 | 9 | 15.37 | 864 |
| 24kd03-39 | 0.75 | 0.4985 | 0.0284 | 0.0657 | 0.0009 | 0.0236 | 0.0005 | 411 | 19 | 410 | 6 | 470 | 10 | 14.15 | 854 |
| 24kd03-40 | 0.81 | 0.4946 | 0.0232 | 0.0657 | 0.0009 | 0.0243 | 0.0005 | 408 | 16 | 410 | 6 | 485 | 9 | 15.17 | 862 |
| 24kd03-41 | 0.91 | 0.5108 | 0.0263 | 0.0660 | 0.0010 | 0.0240 | 0.0005 | 419 | 18 | 412 | 6 | 480 | 10 | 14.18 | 854 |
| 24kd03-42 | 0.79 | 0.4950 | 0.0310 | 0.0654 | 0.0010 | 0.0242 | 0.0006 | 408 | 21 | 408 | 6 | 483 | 11 | 14.84 | 859 |
| 24kd03-43 | 0.74 | 0.4916 | 0.0239 | 0.0656 | 0.0009 | 0.0244 | 0.0005 | 406 | 16 | 409 | 6 | 487 | 10 | 14.12 | 854 |
| 24kd03-44 | 0.55 | 0.4949 | 0.0138 | 0.0656 | 0.0008 | 0.0277 | 0.0004 | 408 | 9 | 410 | 5 | 552 | 8 | 9.14 | 806 |
| 24kd03-45 | 0.74 | 0.5027 | 0.0367 | 0.0658 | 0.0010 | 0.0253 | 0.0006 | 413 | 25 | 411 | 6 | 506 | 12 | 16.76 | 874 |
| 24kd03-46 | 0.84 | 0.4886 | 0.0133 | 0.0660 | 0.0008 | 0.0298 | 0.0004 | 404 | 9 | 412 | 5 | 593 | 7 | 11.77 | 833 |
| 24kd03-47 | 0.79 | 0.5089 | 0.0232 | 0.0658 | 0.0009 | 0.0273 | 0.0005 | 418 | 16 | 411 | 5 | 544 | 9 | 12.79 | 842 |
| 24kd03-48 | 1.17 | 0.5043 | 0.0216 | 0.0660 | 0.0009 | 0.0235 | 0.0004 | 415 | 15 | 412 | 5 | 469 | 7 | 15.98 | 868 |
| 24kd03-49 | 0.81 | 0.4963 | 0.0199 | 0.0660 | 0.0009 | 0.0262 | 0.0004 | 409 | 13 | 412 | 5 | 523 | 8 | 10.71 | 823 |
| 24kd03-51 | 0.80 | 0.5575 | 0.0463 | 0.0663 | 0.0011 | 0.0247 | 0.0006 | 450 | 30 | 414 | 7 | 493 | 13 | 14.77 | 859 |
| 24kd03-52 | 0.76 | 0.4968 | 0.0215 | 0.0660 | 0.0009 | 0.0253 | 0.0005 | 410 | 15 | 412 | 5 | 505 | 9 | 14.28 | 855 |
| 24kd03-53 | 0.75 | 0.4995 | 0.0263 | 0.0658 | 0.0010 | 0.0241 | 0.0005 | 411 | 18 | 410 | 6 | 481 | 11 | 15.93 | 868 |
| 24kd03-54 | 0.79 | 0.4900 | 0.0236 | 0.0661 | 0.0009 | 0.0240 | 0.0005 | 405 | 16 | 412 | 6 | 479 | 9 | 15.89 | 867 |
| **24kd04 (GPS: 36°51'28.63"N, 98°8'30.16"E)** | | | | | | | | | | | | | | | |
| 24kd04-02 | 0.94 | 11.20405 | 0.14239 | 0.4894 | 0.00604 | 0.15608 | 0.00133 | 2540 | 12 | 2568 | 26 | 2931 | 23 | / | / |
| 24kd04-04 | 0.96 | 0.50957 | 0.01503 | 0.06435 | 0.00089 | 0.02972 | 0.00037 | 418 | 10 | 402 | 5 | 592 | 7 | / | / |
| 24kd04-06 | 0.31 | 0.60277 | 0.02308 | 0.07575 | 0.00115 | 0.03085 | 0.00083 | 479 | 15 | 471 | 7 | 614 | 16 | / | / |
| **24kd05 (GPS: 36°51'30.52"N, 98°8'29.90"E)** | | | | | | | | | | | | | | | |
| 24kd05-03 | 1.76 | 0.50542 | 0.01134 | 0.065 | 0.00075 | 0.0241 | 0.00035 | 415 | 8 | 406 | 5 | 481 | 7 | 9.76 | 813 |
| 24kd05-04 | 1.01 | 0.48974 | 0.02913 | 0.06492 | 0.00084 | 0.02291 | 0.00045 | 405 | 20 | 405 | 5 | 458 | 9 | 6.9 | 777 |
| 24kd05-05 | 1.67 | 0.48922 | 0.00855 | 0.0649 | 0.00071 | 0.02375 | 0.00028 | 404 | 6 | 405 | 4 | 474 | 6 | 6.54 | 772 |
| 24kd05-06 | 2.02 | 0.51934 | 0.01366 | 0.065 | 0.00077 | 0.02447 | 0.00043 | 425 | 9 | 406 | 5 | 489 | 8 | 11.59 | 831 |
| 24kd05-07 | 1.65 | 0.51159 | 0.01028 | 0.06496 | 0.00072 | 0.02478 | 0.00031 | 420 | 7 | 406 | 4 | 495 | 6 | 4.68 | 740 |
| 24kd05-10 | 1.48 | 0.50477 | 0.00881 | 0.06486 | 0.00071 | 0.0232 | 0.00027 | 415 | 6 | 405 | 4 | 464 | 5 | 8.39 | 797 |
| 24kd05-12 | 1.89 | 0.50877 | 0.00996 | 0.06492 | 0.00072 | 0.0243 | 0.00032 | 418 | 7 | 405 | 4 | 485 | 6 | 6.03 | 764 |
| 24kd05-14 | 1.76 | 0.47217 | 0.00888 | 0.06496 | 0.00071 | 0.02345 | 0.0003 | 393 | 6 | 406 | 4 | 469 | 6 | 14.46 | 856 |
| 24kd05-16 | 0.70 | 0.49275 | 0.02285 | 0.06488 | 0.00088 | 0.02268 | 0.00037 | 407 | 16 | 405 | 5 | 453 | 7 | 6.16 | 766 |
| 24kd05-19 | 1.68 | 0.55146 | 0.01153 | 0.06497 | 0.00073 | 0.02765 | 0.00035 | 446 | 8 | 406 | 4 | 551 | 7 | 10.88 | 825 |
| 24kd05-20 | 1.80 | 0.48757 | 0.00888 | 0.06491 | 0.00071 | 0.02255 | 0.00028 | 403 | 6 | 405 | 4 | 451 | 6 | 8.36 | 797 |
| 24kd05-25 | 1.49 | 0.52813 | 0.01159 | 0.06488 | 0.00072 | 0.02505 | 0.00032 | 431 | 8 | 405 | 4 | 500 | 6 | 9.01 | 804 |
| 24kd05-26 | 1.79 | 0.50375 | 0.00951 | 0.06495 | 0.0007 | 0.02503 | 0.00029 | 414 | 6 | 406 | 4 | 500 | 6 | 5.76 | 759 |
| 24kd05-27 | 1.45 | 0.54909 | 0.01541 | 0.06527 | 0.0008 | 0.02456 | 0.00039 | 444 | 10 | 408 | 5 | 490 | 8 | 12.11 | 836 |
| 24kd05-28 | 1.13 | 0.49983 | 0.01011 | 0.06507 | 0.00072 | 0.02333 | 0.00027 | 412 | 7 | 406 | 4 | 466 | 5 | 8.67 | 800 |
| 24kd05-29 | 1.73 | 0.49673 | 0.00925 | 0.06501 | 0.00071 | 0.0241 | 0.00029 | 409 | 6 | 406 | 4 | 481 | 6 | 6.57 | 772 |
| 24kd05-30 | 1.72 | 0.54778 | 0.01016 | 0.06508 | 0.00071 | 0.02661 | 0.00031 | 444 | 7 | 406 | 4 | 531 | 6 | 9.72 | 812 |
| 24kd05-32 | 1.80 | 0.50156 | 0.00842 | 0.06504 | 0.0007 | 0.02486 | 0.00029 | 413 | 6 | 406 | 4 | 496 | 6 | 6 | 763 |
| 24kd05-33 | 1.70 | 0.48596 | 0.00948 | 0.06506 | 0.00072 | 0.02349 | 0.00029 | 402 | 6 | 406 | 4 | 469 | 6 | 6.77 | 775 |
| 24kd05-35 | 1.95 | 0.48763 | 0.00902 | 0.06503 | 0.00071 | 0.02478 | 0.00031 | 403 | 6 | 406 | 4 | 495 | 6 | 7.18 | 781 |
| 24kd05-36 | 1.91 | 0.51436 | 0.01026 | 0.06478 | 0.00072 | 0.02617 | 0.00034 | 421 | 7 | 405 | 4 | 522 | 7 | 7.03 | 779 |
| 24kd05-37 | 1.84 | 0.4924 | 0.01019 | 0.06514 | 0.00073 | 0.02364 | 0.00031 | 407 | 7 | 407 | 4 | 472 | 6 | 5.44 | 754 |
| 24kd05-38 | 1.78 | 0.489 | 0.00948 | 0.06505 | 0.00071 | 0.02391 | 0.0003 | 404 | 6 | 406 | 4 | 478 | 6 | 8.94 | 804 |
| 24kd05-40 | 1.98 | 0.50238 | 0.00988 | 0.06496 | 0.00072 | 0.02451 | 0.00032 | 413 | 7 | 406 | 4 | 489 | 6 | 7 | 779 |
| 24kd05-41 | 1.11 | 0.52534 | 0.01749 | 0.06518 | 0.00083 | 0.02253 | 0.00036 | 429 | 12 | 407 | 5 | 450 | 7 | 12.14 | 837 |
| 24kd05-42 | 1.72 | 0.50436 | 0.01086 | 0.06496 | 0.00072 | 0.02425 | 0.00031 | 415 | 7 | 406 | 4 | 484 | 6 | 5.27 | 751 |
| 24kd05-43 | 1.09 | 0.50986 | 0.01634 | 0.06516 | 0.00082 | 0.02139 | 0.00034 | 418 | 11 | 407 | 5 | 428 | 7 | 5.83 | 761 |
| 24kd05-44 | 1.61 | 0.49792 | 0.01113 | 0.06499 | 0.00073 | 0.02241 | 0.0003 | 410 | 8 | 406 | 4 | 448 | 6 | 6.09 | 765 |
| 24kd05-45 | 1.28 | 0.50366 | 0.01153 | 0.06483 | 0.00073 | 0.0239 | 0.0003 | 414 | 8 | 405 | 4 | 477 | 6 | 5.47 | 754 |
| 24kd05-47 | 1.96 | 0.49865 | 0.00843 | 0.06499 | 0.00069 | 0.02625 | 0.00029 | 411 | 6 | 406 | 4 | 524 | 6 | 5.54 | 756 |
| 24kd05-48 | 2.27 | 0.51541 | 0.01138 | 0.06489 | 0.00072 | 0.02533 | 0.00034 | 422 | 8 | 405 | 4 | 506 | 7 | 8.05 | 793 |
| 24kd05-50 | 1.78 | 0.49541 | 0.01046 | 0.06498 | 0.00072 | 0.02355 | 0.00031 | 409 | 7 | 406 | 4 | 470 | 6 | 5.8 | 760 |
| 24kd05-51 | 1.80 | 0.4829 | 0.01017 | 0.06498 | 0.00072 | 0.0224 | 0.0003 | 400 | 7 | 406 | 4 | 448 | 6 | 6.02 | 764 |
| 24kd05-52 | 1.77 | 0.51253 | 0.01046 | 0.06493 | 0.00072 | 0.02375 | 0.0003 | 420 | 7 | 406 | 4 | 474 | 6 | 8.23 | 795 |
| 24kd05-54 | 1.37 | 0.52927 | 0.01207 | 0.06505 | 0.00074 | 0.02498 | 0.00031 | 431 | 8 | 406 | 4 | 499 | 6 | 7.28 | 782 |
| **23ks04 (GPS: 36°6'3.52"N, 94°48'28.06"E)** | | | | | | | | | | | | | | | |
| 23ks04-01 | 0.34 | 0.4550 | 0.0126 | 0.0658 | 0.0008 | 0.0227 | 0.0004 | 381 | 9 | 411 | 5 | 454 | 8 | 4.91 | 744 |
| 23ks04-02 | 0.45 | 0.4932 | 0.0120 | 0.0657 | 0.0007 | 0.0194 | 0.0003 | 407 | 8 | 410 | 4 | 389 | 6 | 12.5 | 840 |
| 23ks04-03 | 0.41 | 0.4749 | 0.0093 | 0.0656 | 0.0007 | 0.0201 | 0.0003 | 395 | 6 | 410 | 4 | 402 | 5 | 5.8 | 760 |
| 23ks04-04 | 0.41 | 0.4782 | 0.0184 | 0.0660 | 0.0008 | 0.0213 | 0.0005 | 397 | 13 | 412 | 5 | 425 | 10 | 12.34 | 838 |
| 23ks04-06 | 0.75 | 0.4588 | 0.0144 | 0.0657 | 0.0008 | 0.0186 | 0.0003 | 383 | 10 | 410 | 5 | 373 | 6 | 11.43 | 830 |
| 23ks04-07 | 0.40 | 0.4749 | 0.0095 | 0.0658 | 0.0007 | 0.0209 | 0.0003 | 395 | 7 | 411 | 4 | 418 | 6 | 5.55 | 756 |
| 23ks04-08 | 0.55 | 0.4551 | 0.0163 | 0.0659 | 0.0008 | 0.0204 | 0.0004 | 381 | 11 | 411 | 5 | 407 | 8 | 12.58 | 841 |
| 23ks04-09 | 0.44 | 0.4727 | 0.0096 | 0.0657 | 0.0007 | 0.0213 | 0.0003 | 393 | 7 | 410 | 4 | 427 | 5 | 19.04 | 889 |
| 23ks04-10 | 0.40 | 0.4628 | 0.0108 | 0.0658 | 0.0007 | 0.0194 | 0.0003 | 386 | 7 | 411 | 4 | 388 | 6 | 7.06 | 779 |
| 23ks04-11 | 0.39 | 0.4820 | 0.0111 | 0.0658 | 0.0007 | 0.0217 | 0.0003 | 399 | 8 | 411 | 4 | 433 | 7 | 7.16 | 781 |
| 23ks04-12 | 0.39 | 0.4680 | 0.0076 | 0.0658 | 0.0007 | 0.0183 | 0.0002 | 390 | 5 | 411 | 4 | 367 | 4 | 2.86 | 696 |
| 23ks04-13 | 0.44 | 0.4600 | 0.0095 | 0.0658 | 0.0007 | 0.0214 | 0.0003 | 384 | 7 | 411 | 4 | 428 | 6 | 4.76 | 741 |
| 23ks04-14 | 0.48 | 0.4812 | 0.0127 | 0.0657 | 0.0007 | 0.0187 | 0.0003 | 399 | 9 | 410 | 4 | 374 | 6 | 7.95 | 791 |
| 23ks04-15 | 0.42 | 0.4803 | 0.0109 | 0.0657 | 0.0007 | 0.0193 | 0.0003 | 398 | 7 | 410 | 4 | 385 | 6 | 5.92 | 762 |
| 23ks04-16 | 0.75 | 0.4447 | 0.0112 | 0.0658 | 0.0007 | 0.0185 | 0.0002 | 374 | 8 | 411 | 4 | 370 | 5 | 5.23 | 750 |
| 23ks04-17 | 0.49 | 0.4567 | 0.0098 | 0.0656 | 0.0007 | 0.0201 | 0.0003 | 382 | 7 | 410 | 4 | 402 | 5 | 6.92 | 777 |
| 23ks04-18 | 0.70 | 0.4863 | 0.0181 | 0.0657 | 0.0008 | 0.0195 | 0.0004 | 402 | 12 | 410 | 5 | 391 | 7 | 15.28 | 863 |
| 23ks04-19 | 0.55 | 0.4760 | 0.0114 | 0.0657 | 0.0007 | 0.0175 | 0.0003 | 395 | 8 | 410 | 4 | 350 | 6 | 6.06 | 764 |
| 23ks04-20 | 0.43 | 0.4802 | 0.0103 | 0.0658 | 0.0007 | 0.0188 | 0.0003 | 398 | 7 | 411 | 4 | 377 | 6 | 5.43 | 754 |
| 23ks04-21 | 0.60 | 0.4833 | 0.0126 | 0.0657 | 0.0008 | 0.0185 | 0.0003 | 400 | 9 | 410 | 5 | 371 | 6 | 12.9 | 843 |
| 23ks04-22 | 0.58 | 0.4865 | 0.0182 | 0.0658 | 0.0008 | 0.0191 | 0.0004 | 402 | 12 | 411 | 5 | 382 | 8 | 12.4 | 839 |
| 23ks04-24 | 0.48 | 0.4785 | 0.0116 | 0.0658 | 0.0007 | 0.0183 | 0.0003 | 397 | 8 | 411 | 4 | 366 | 6 | 7.33 | 783 |
| 23ks04-25 | 0.49 | 0.4806 | 0.0128 | 0.0659 | 0.0007 | 0.0184 | 0.0003 | 398 | 9 | 411 | 4 | 369 | 6 | 6.17 | 766 |
| 23ks04-26 | 0.63 | 0.4839 | 0.0128 | 0.0658 | 0.0007 | 0.0175 | 0.0003 | 401 | 9 | 410 | 4 | 351 | 5 | 9.45 | 809 |
| 23ks04-27 | 0.45 | 0.4951 | 0.0130 | 0.0657 | 0.0007 | 0.0179 | 0.0004 | 408 | 9 | 410 | 4 | 359 | 7 | 4.43 | 735 |
| 23ks04-28 | 0.49 | 0.4941 | 0.0139 | 0.0658 | 0.0007 | 0.0174 | 0.0003 | 408 | 9 | 411 | 4 | 348 | 6 | 4.41 | 734 |
| 23ks04-29 | 0.64 | 0.4742 | 0.0151 | 0.0657 | 0.0008 | 0.0155 | 0.0003 | 394 | 10 | 410 | 5 | 311 | 6 | 10.4 | 820 |
| 23ks04-30 | 0.48 | 0.4878 | 0.0113 | 0.0659 | 0.0007 | 0.0183 | 0.0003 | 403 | 8 | 411 | 4 | 367 | 6 | 6.68 | 774 |
| 23ks04-31 | 0.47 | 0.4918 | 0.0145 | 0.0657 | 0.0008 | 0.0186 | 0.0003 | 406 | 10 | 410 | 5 | 372 | 7 | 6.4 | 770 |
| 23ks04-32 | 0.46 | 0.4889 | 0.0174 | 0.0657 | 0.0008 | 0.0186 | 0.0004 | 404 | 12 | 410 | 5 | 372 | 8 | 9.12 | 806 |
| 23ks04-33 | 0.50 | 0.4677 | 0.0132 | 0.0658 | 0.0007 | 0.0194 | 0.0003 | 390 | 9 | 411 | 4 | 388 | 6 | 5.15 | 749 |
| 23ks04-34 | 0.55 | 0.4882 | 0.0101 | 0.0659 | 0.0007 | 0.0171 | 0.0002 | 404 | 7 | 411 | 4 | 343 | 5 | 15.45 | 864 |
| 23ks04-35 | 0.52 | 0.4723 | 0.0166 | 0.0659 | 0.0009 | 0.0191 | 0.0005 | 393 | 11 | 411 | 5 | 382 | 9 | 6.07 | 764 |
| 23ks04-36 | 0.44 | 0.4598 | 0.0115 | 0.0659 | 0.0007 | 0.0175 | 0.0003 | 384 | 8 | 411 | 4 | 350 | 6 | 5.99 | 763 |
| 23ks04-37 | 0.47 | 0.4956 | 0.0129 | 0.0658 | 0.0007 | 0.0178 | 0.0003 | 409 | 9 | 411 | 4 | 357 | 6 | 12.16 | 837 |
| 23ks04-38 | 0.53 | 0.4962 | 0.0153 | 0.0657 | 0.0008 | 0.0191 | 0.0003 | 409 | 10 | 410 | 5 | 382 | 7 | 8.37 | 797 |
| 23ks04-39 | 0.48 | 0.4978 | 0.0121 | 0.0657 | 0.0007 | 0.0202 | 0.0003 | 410 | 8 | 410 | 4 | 405 | 6 | 11.97 | 835 |
| 23ks04-40 | 0.51 | 0.4963 | 0.0145 | 0.0658 | 0.0007 | 0.0199 | 0.0003 | 409 | 10 | 411 | 4 | 399 | 6 | 21.78 | 906 |
| 23ks04-41 | 0.95 | 0.4824 | 0.0161 | 0.0656 | 0.0008 | 0.0173 | 0.0003 | 400 | 11 | 410 | 5 | 347 | 5 | 4.75 | 741 |
| 23ks04-42 | 0.44 | 0.4971 | 0.0082 | 0.0657 | 0.0007 | 0.0189 | 0.0002 | 410 | 6 | 410 | 4 | 378 | 4 | 5.26 | 751 |
| 23ks04-43 | 0.43 | 0.4894 | 0.0105 | 0.0658 | 0.0007 | 0.0194 | 0.0003 | 405 | 7 | 411 | 4 | 389 | 6 | 18.97 | 889 |
| 23ks04-44 | 0.55 | 0.4965 | 0.0328 | 0.0658 | 0.0010 | 0.0200 | 0.0006 | 409 | 22 | 411 | 6 | 400 | 12 | 8.48 | 798 |
| 23ks04-45 | 0.48 | 0.5001 | 0.0113 | 0.0658 | 0.0007 | 0.0191 | 0.0003 | 412 | 8 | 411 | 4 | 383 | 5 | 12.25 | 838 |
| 23ks04-46 | 0.49 | 0.4953 | 0.0224 | 0.0658 | 0.0008 | 0.0181 | 0.0005 | 408 | 15 | 411 | 5 | 362 | 10 | 6.27 | 768 |
| 23ks04-48 | 0.46 | 0.4991 | 0.0114 | 0.0658 | 0.0007 | 0.0192 | 0.0003 | 411 | 8 | 410 | 4 | 385 | 5 | 6.37 | 769 |
| 23ks04-49 | 0.43 | 0.5006 | 0.0131 | 0.0656 | 0.0007 | 0.0187 | 0.0003 | 412 | 9 | 410 | 4 | 373 | 6 | 7.07 | 780 |
| 23ks04-50 | 0.60 | 0.4993 | 0.0138 | 0.0658 | 0.0007 | 0.0174 | 0.0003 | 411 | 9 | 411 | 4 | 349 | 5 | 6.61 | 773 |
| 23ks04-51 | 0.48 | 0.4990 | 0.0115 | 0.0657 | 0.0007 | 0.0194 | 0.0003 | 411 | 8 | 410 | 4 | 388 | 5 | 6.34 | 769 |
| 23ks04-52 | 0.51 | 0.4975 | 0.0103 | 0.0657 | 0.0007 | 0.0186 | 0.0002 | 410 | 7 | 410 | 4 | 373 | 5 | 6.28 | 768 |
| 23ks04-53 | 0.43 | 0.4975 | 0.0109 | 0.0658 | 0.0007 | 0.0212 | 0.0003 | 410 | 7 | 411 | 4 | 424 | 6 | 7.07 | 780 |
| 23ks04-54 | 0.42 | 0.5003 | 0.0141 | 0.0658 | 0.0007 | 0.0196 | 0.0004 | 412 | 10 | 411 | 4 | 393 | 7 | 4.91 | 744 |
| **23ks31 (GPS: 36°6'37.53"N, 94°53'22.22"E)** | | | | | | | | | | | | | | | |
| 23ks31-01 | 0.38 | 0.4859 | 0.0141 | 0.0659 | 0.0007 | 0.0214 | 0.0004 | 402 | 10 | 411 | 4 | 428 | 8 | 9.36 | 808 |
| 23ks31-02 | 0.40 | 0.4856 | 0.0096 | 0.0659 | 0.0007 | 0.0224 | 0.0003 | 402 | 7 | 412 | 4 | 448 | 6 | 5.7 | 758 |
| 23ks31-03 | 0.54 | 0.5223 | 0.0111 | 0.0659 | 0.0007 | 0.0226 | 0.0003 | 427 | 7 | 411 | 4 | 451 | 6 | 8.16 | 794 |
| 23ks31-04 | 0.42 | 0.5222 | 0.0133 | 0.0660 | 0.0007 | 0.0231 | 0.0004 | 427 | 9 | 412 | 4 | 462 | 7 | 6.97 | 778 |
| 23ks31-06 | 0.43 | 0.4955 | 0.0095 | 0.0659 | 0.0007 | 0.0228 | 0.0003 | 409 | 6 | 412 | 4 | 456 | 6 | 7.04 | 779 |
| 23ks31-07 | 0.39 | 0.5103 | 0.0172 | 0.0660 | 0.0008 | 0.0258 | 0.0005 | 419 | 12 | 412 | 5 | 514 | 10 | 11.92 | 835 |
| 23ks31-09 | 0.49 | 0.5565 | 0.0170 | 0.0659 | 0.0007 | 0.0239 | 0.0004 | 449 | 11 | 411 | 4 | 478 | 8 | 12.29 | 838 |
| 23ks31-10 | 0.43 | 0.4903 | 0.0349 | 0.0661 | 0.0013 | 0.0245 | 0.0010 | 405 | 24 | 413 | 8 | 488 | 21 | 11.83 | 834 |
| 23ks31-12 | 0.43 | 0.4907 | 0.0089 | 0.0659 | 0.0007 | 0.0224 | 0.0003 | 405 | 6 | 411 | 4 | 448 | 5 | 4.23 | 730 |
| 23ks31-14 | 0.62 | 0.5040 | 0.0078 | 0.0659 | 0.0007 | 0.0224 | 0.0002 | 414 | 5 | 411 | 4 | 448 | 4 | 6.03 | 764 |
| 23ks31-15 | 0.52 | 0.4917 | 0.0118 | 0.0659 | 0.0007 | 0.0239 | 0.0003 | 406 | 8 | 412 | 4 | 477 | 7 | 14.29 | 855 |
| 23ks31-16 | 0.51 | 0.5298 | 0.0104 | 0.0659 | 0.0007 | 0.0232 | 0.0003 | 432 | 7 | 412 | 4 | 464 | 6 | 11.99 | 835 |
| 23ks31-17 | 0.53 | 0.5174 | 0.0120 | 0.0659 | 0.0007 | 0.0227 | 0.0003 | 423 | 8 | 411 | 4 | 453 | 6 | 9.91 | 814 |
| 23ks31-18 | 0.43 | 0.4989 | 0.0111 | 0.0659 | 0.0007 | 0.0226 | 0.0003 | 411 | 8 | 412 | 4 | 451 | 6 | 7.28 | 782 |
| 23ks31-19 | 0.45 | 0.5224 | 0.0091 | 0.0659 | 0.0007 | 0.0241 | 0.0003 | 427 | 6 | 411 | 4 | 480 | 5 | 5.22 | 750 |
| 23ks31-20 | 0.38 | 0.4780 | 0.0089 | 0.0660 | 0.0007 | 0.0250 | 0.0003 | 397 | 6 | 412 | 4 | 499 | 6 | 6.04 | 764 |
| 23ks31-21 | 0.49 | 0.4772 | 0.0139 | 0.0659 | 0.0007 | 0.0225 | 0.0004 | 396 | 10 | 412 | 4 | 449 | 7 | 8.73 | 801 |
| 23ks31-22 | 0.50 | 0.4946 | 0.0118 | 0.0660 | 0.0007 | 0.0234 | 0.0003 | 408 | 8 | 412 | 4 | 467 | 7 | 5.03 | 746 |
| 23ks31-23 | 0.39 | 0.4972 | 0.0115 | 0.0660 | 0.0007 | 0.0233 | 0.0004 | 410 | 8 | 412 | 4 | 465 | 7 | 6.97 | 778 |
| 23ks31-24 | 0.41 | 0.4904 | 0.0164 | 0.0659 | 0.0008 | 0.0207 | 0.0005 | 405 | 11 | 411 | 5 | 413 | 9 | 9.92 | 815 |
| 23ks31-25 | 0.38 | 0.4949 | 0.0111 | 0.0659 | 0.0007 | 0.0231 | 0.0003 | 408 | 8 | 411 | 4 | 461 | 7 | 7.22 | 782 |
| 23ks31-26 | 0.57 | 0.5024 | 0.0187 | 0.0660 | 0.0008 | 0.0233 | 0.0005 | 413 | 13 | 412 | 5 | 466 | 9 | 12.52 | 840 |
| 23ks31-27 | 0.43 | 0.5093 | 0.0150 | 0.0660 | 0.0008 | 0.0226 | 0.0004 | 418 | 10 | 412 | 5 | 452 | 8 | 9.78 | 813 |
| 23ks31-29 | 0.38 | 0.4953 | 0.0108 | 0.0660 | 0.0007 | 0.0230 | 0.0003 | 409 | 7 | 412 | 4 | 459 | 7 | 3.38 | 710 |
| 23ks31-31 | 0.52 | 0.5269 | 0.0076 | 0.0659 | 0.0006 | 0.0238 | 0.0002 | 430 | 5 | 412 | 4 | 475 | 5 | 10.83 | 824 |
| 23ks31-32 | 0.52 | 0.5048 | 0.0198 | 0.0658 | 0.0009 | 0.0199 | 0.0005 | 415 | 13 | 411 | 5 | 399 | 10 | 3.25 | 707 |
| 23ks31-33 | 0.44 | 0.4838 | 0.0114 | 0.0660 | 0.0007 | 0.0212 | 0.0003 | 401 | 8 | 412 | 4 | 424 | 6 | 7.6 | 787 |
| 23ks31-34 | 0.40 | 0.4987 | 0.0187 | 0.0660 | 0.0008 | 0.0233 | 0.0006 | 411 | 13 | 412 | 5 | 466 | 11 | 5.64 | 757 |
| 23ks31-35 | 0.58 | 0.4993 | 0.0112 | 0.0659 | 0.0007 | 0.0191 | 0.0003 | 411 | 8 | 411 | 4 | 383 | 5 | 8.13 | 794 |
| 23ks31-36 | 0.37 | 0.4875 | 0.0105 | 0.0660 | 0.0007 | 0.0215 | 0.0003 | 403 | 7 | 412 | 4 | 430 | 6 | 4.55 | 737 |
| 23ks31-37 | 0.41 | 0.4894 | 0.0136 | 0.0659 | 0.0007 | 0.0201 | 0.0004 | 404 | 9 | 412 | 4 | 403 | 8 | 5.95 | 762 |
| 23ks31-38 | 0.61 | 0.5093 | 0.0187 | 0.0660 | 0.0008 | 0.0227 | 0.0004 | 418 | 13 | 412 | 5 | 454 | 8 | 25.55 | 926 |
| 23ks31-39 | 0.54 | 0.5023 | 0.0150 | 0.0658 | 0.0008 | 0.0210 | 0.0004 | 413 | 10 | 411 | 5 | 419 | 7 | 7.23 | 782 |
| 23ks31-40 | 0.45 | 0.4986 | 0.0191 | 0.0659 | 0.0009 | 0.0224 | 0.0005 | 411 | 13 | 412 | 5 | 448 | 10 | 9.96 | 815 |
| 23ks31-42 | 0.40 | 0.5005 | 0.0157 | 0.0658 | 0.0008 | 0.0204 | 0.0004 | 412 | 11 | 411 | 5 | 409 | 9 | 9.03 | 805 |
| 23ks31-43 | 0.47 | 0.5253 | 0.0231 | 0.0661 | 0.0009 | 0.0215 | 0.0006 | 429 | 15 | 412 | 6 | 430 | 11 | 4.29 | 732 |
| 23ks31-44 | 0.40 | 0.4894 | 0.0118 | 0.0660 | 0.0007 | 0.0203 | 0.0003 | 404 | 8 | 412 | 4 | 406 | 7 | 10.91 | 825 |
| 23ks31-46 | 0.32 | 0.4955 | 0.0117 | 0.0659 | 0.0007 | 0.0228 | 0.0004 | 409 | 8 | 411 | 4 | 455 | 8 | 6.68 | 774 |
| 23ks31-47 | 0.64 | 0.4798 | 0.0161 | 0.0659 | 0.0008 | 0.0219 | 0.0004 | 398 | 11 | 411 | 5 | 437 | 8 | 6.99 | 778 |
| 23ks31-48 | 0.67 | 0.4863 | 0.0139 | 0.0660 | 0.0007 | 0.0208 | 0.0003 | 402 | 9 | 412 | 4 | 416 | 6 | 6.09 | 765 |
| 23ks31-49 | 0.59 | 0.5093 | 0.0134 | 0.0660 | 0.0007 | 0.0193 | 0.0003 | 418 | 9 | 412 | 4 | 386 | 6 | 12.84 | 843 |
| 23ks31-50 | 0.27 | 0.5180 | 0.0148 | 0.0659 | 0.0008 | 0.0209 | 0.0005 | 424 | 10 | 412 | 5 | 417 | 10 | 9.59 | 811 |
| 23ks31-51 | 0.62 | 0.4895 | 0.0183 | 0.0659 | 0.0008 | 0.0206 | 0.0004 | 405 | 12 | 411 | 5 | 411 | 8 | 14.92 | 860 |
| 23ks31-54 | 0.47 | 0.4845 | 0.0280 | 0.0658 | 0.0011 | 0.0192 | 0.0007 | 401 | 19 | 411 | 6 | 385 | 14 | 10.52 | 821 |

Notes: Ti-in-zircon thermometer eq: log (ppm Ti-in-zircon) = (5.711 ± 0.072) - (4800 ± 86)/T(k) - logα_SiO2_ + logα_TiO2_

Table S2. EDS of representative samples with measured spots in Fig. S4.

| **sample** | **spot** | **Ti** | **Fe** | **O** | **Ti:Fe ratio** |
| --- | --- | --- | --- | --- | --- |
| **kd05-2** | 01 | 0 | 49.37 | 50.63 | 0 |
|  | 02 | 0 | 49.63 | 50.37 | 0 |
|  | 03 | 1.24 | 44.22 | 52.35 | 0.03 |
|  | 04 | 0 | 48.37 | 51.63 | 0 |
| **kd09-1** | 01 | 1.05 | 43.54 | 54.24 | 0.02 |
|  | 02 | 1.15 | 45.39 | 53.32 | 0.03 |
|  | 03 | 0.70 | 45.58 | 53.39 | 0.02 |
|  | 04 | 0.80 | 47.32 | 51.87 | 0.02 |
|  | 05 | 2.48 | 43.52 | 54.00 | 0.06 |
|  | 06 | 1.94 | 43.92 | 53.93 | 0.04 |
|  | 07 | 0.26 | 43.45 | 54.27 | 0.01 |
|  | 08 | 0.28 | 11.42 | 65.16 | 0.02 |
|  | 09 | 0.55 | 21.53 | 61.79 | 0.03 |
|  | 10 | 0.80 | 37.26 | 55.41 | 0.02 |
| **kd15-3** | 01 | 1.31 | 36.80 | 55.67 | 0.04 |
|  | 02 | 0.88 | 44.33 | 53.75 | 0.02 |
|  | 03 | 0.93 | 45.00 | 53.69 | 0.02 |
|  | 04 | 0.81 | 40.92 | 54.92 | 0.02 |
|  | 05 | 1.38 | 40.50 | 54.71 | 0.03 |
|  | 06 | 0.89 | 45.44 | 53.51 | 0.02 |
|  | 07 | 0.72 | 40.30 | 55.88 | 0.02 |
|  | 08 | 0.55 | 35.42 | 57.83 | 0.02 |
| **kd20-8** | 01 | 0.62 | 43.36 | 54.70 | 0.01 |
|  | 02 | 0.58 | 44.19 | 54.48 | 0.01 |
|  | 03 | 1.08 | 46.04 | 51.93 | 0.02 |
|  | 04 | 1.48 | 46.71 | 51.41 | 0.03 |
|  | 05 | 0.61 | 29.72 | 60.29 | 0.02 |
|  | 06 | 0.83 | 42.23 | 56.27 | 0.02 |
|  | 07 | 0.67 | 36.02 | 56.40 | 0.02 |
| **kd23-7** | 01 | 6.44 | 41.39 | 52.17 | 0.16 |
|  | 02 | 4.18 | 44.45 | 51.37 | 0.09 |
|  | 03 | 2.45 | 45.23 | 51.75 | 0.05 |
|  | 04 | 5.43 | 41.60 | 52.97 | 0.13 |
|  | 05 | 3.65 | 44.69 | 51.66 | 0.08 |
|  | 06 | 1.25 | 45.42 | 52.14 | 0.03 |
|  | 07 | 0.46 | 19.58 | 64.23 | 0.02 |
|  | 08 | 1.69 | 44.25 | 53.57 | 0.04 |
|  | 09 | 2.20 | 42.12 | 54.62 | 0.05 |

**Table S3. Characteristic remanent directions for Devonian volcanic rocks in the Qaidam-Kunlun continent.**

| **Sample/**  **site** | **Direction fit** | **Demagnetization steps (°C)** | **N** | **Dg** | **Ig** | **Ds** | **Is** | **κ** | **MAD** |  |
| --- | --- | --- | --- | --- | --- | --- | --- | --- | --- | --- |
| **Site kd01 (GPS: 36°51'53.73"N, 98°8'55.26"E)** | | | | | | | | | |  |
| kd1-1 | PCA | 350-600 | 9 | 358 | -34.7 | 13.9 | -22.2 | 158.6 | 4.1 |  |
| kd1-5 | PCA | 400-600 | 6 | 350.7 | -43.5 | 14.4 | -32.7 | 40.9 | 10.6 |  |
| kd1-6 | PCA | 400-600 | 6 | 343.7 | -43.9 | 9.9 | -36.2 | 95.2 | 6.9 |  |
| kd1-7 | PCA | 400-630 | 7 | 344.7 | -36.5 | 5.1 | -29.8 | 48 | 8.8 |  |
| site mean |  |  | 4 | 349.4 | -39.8 | 10.9 | -30.3 | 132.9 | 8.0 | |
| **Site kd02 (GPS: 36°51'53.65"N, 98°8'55.50"E)** | | | | | | | | | |  |
| kd2-1 | PCA | 350-630 | 10 | 336.2 | -39.8 | 1.3 | -36.5 | 81 | 5.4 |  |
| kd2-2 | PCA | 350-630 | 9 | 339.8 | -44.7 | 7.9 | -38.6 | 74.6 | 6 |  |
| kd2-3 | PCA | 350-630 | 10 | 348.1 | -35.9 | 7.3 | -27.8 | 72.8 | 5.7 |  |
| kd2-4 | PCA | 350-630 | 10 | 5 | -40.4 | 22.3 | -24.2 | 39.3 | 7.8 |  |
| site mean |  |  | 4 | 347.3 | -40.7 | 10.0 | -32.0 | 61.8 | 11.8 |  |
| **Site kd03 (GPS: 36°51'54.40"N, 98°8'56.29"E)** | | | | | | | | | |  |
| kd3-1 | PCA | 350-630 | 11 | 344.7 | -43.2 | 10 | -35.1 | 17.6 | 11.2 |  |
| kd3-2 | PCA | 350-630 | 11 | 338.7 | -39.8 | 3.1 | -35.4 | 55.2 | 6.2 |  |
| kd3-3 | PCA | 350-630 | 10 | 329.4 | -33.5 | 351.2 | -35 | 121.5 | 4.4 |  |
| kd3-4 | PCA | 350-630 | 10 | 331.5 | -37.4 | 355.8 | -37 | 90.7 | 5.1 |  |
| kd3-5 | PCA | 400-630 | 7 | 339.2 | -39.8 | 3.5 | -35 | 29.5 | 11.3 |  |
| kd3-6 | PCA | 400-600 | 6 | 350.2 | -43.8 | 14.2 | -33.1 | 213 | 4.6 |  |
| site-mean |  |  | 6 | 338.6 | -39.8 | 3.0 | -35.4 | 129.9 | 5.9 |  |
| **Site kd04 (GPS: 36°51'58.53"N, 98°8'48.69"E)** | | | | | | | | | |  |
| kd4-1 | PCA | 400-650 | 11 | 320 | -53 | 3.7 | -53.7 | 57 | 6.1 |  |
| kd4-3 | PCA | 400-630 | 9 | 304.8 | -50.2 | 349.3 | -59.6 | 60 | 6.7 |  |
| kd4-4 | PCA | 400-630 | 9 | 303.4 | -46.8 | 343.2 | -58.2 | 116 | 4.8 |  |
| kd4-5 | PCA | 400-630 | 7 | 281.8 | -56.9 | 347.8 | -74.7 | 87.2 | 6.5 |  |
| site mean |  |  | 4 | 303.1 | -52.4 | 351.9 | -61.8 | 63.5 | 11.6 |  |
| **Site kd05 (GPS: 36°51'56.47"N, 98°8'48.97"E)** | | | | | | | | | |  |
| kd5-1 | PCA | 400-650 | 7 | 316.4 | -50.3 | 357.9 | -53.8 | 57.9 | 8 |  |
| kd5-3 | PCA | 400-650 | 7 | 315.4 | -53.7 | 2 | -56.3 | 65.7 | 7.5 |  |
| kd5-4 | PCA | 400-650 | 10 | 308.9 | -31.4 | 331.6 | -43.9 | 84 | 5.3 |  |
| kd5-6 | PCA | 450-630 | 8 | 298.1 | -47.9 | 340.1 | -61.6 | 20.3 | 12.6 |  |
| site mean |  |  | 4 | 309.4 | -46.1 | 347.2 | -54.6 | 50.4 | 13.1 |  |
| **Site kd06 (GPS: 36°51'53.65"N, 98°8'55.50"E)** | | | | | | | | | |  |
| kd6-2 | PCA | 500-580 | 4 | 303.5 | -32.6 | 327.4 | -47.6 | 65.9 | 11.4 |  |
| kd6-4 | PCA | 400-650 | 10 | 322.9 | -36.9 | 348.6 | -41 | 48.6 | 7 |  |
| kd6-5 | PCA | 400-650 | 10 | 312 | -36.9 | 339.4 | -46.6 | 63.7 | 6.1 |  |
| kd6-6 | PCA | 400-650 | 7 | 317.2 | -34.3 | 341.6 | -41.9 | 22.2 | 13.1 |  |
| site mean |  |  | 4 | 313.8 | -35.4 | 339.6 | -44.5 | 131.3 | 8.0 |  |
| **Site kd07 (36°51'56.54"N, 98°8'48.71"E)** | | | | | | | | | |  |
| kd7-3 | PCA | 400-650 | 10 | 322.8 | -8.6 | 329.9 | -17.7 | 31.1 | 8.8 |  |
| kd7-5 | PCA | 400-660 | 9 | 327.5 | -38 | 353.2 | -39.5 | 33 | 9.1 |  |
| kd7-7 | PCA | 560-650 | 5 | 326.9 | -11.1 | 335 | -17.8 | 35.6 | 13 |  |
| site mean |  |  | 3 | 325.6 | -19.2 | 338.4 | -25.3 | 24.4 | 25.5 |  |
| **Site kd08 (36° 51′48.18″ N, 98°8′45.56″E)** | | | | | | | | | |  |
| kd8-1 | PCA | 300-665 | 13 | 55.7 | -52.3 | 61.7 | -22.2 | 9.6 | 14.1 |  |
| kd8-2 | PCA | 350-680 | 13 | 350.7 | -45.6 | 15.9 | -34.4 | 16.6 | 10.5 |  |
| kd8-5 | PCA | 500-690 | 11 | 118.8 | 4.8 | 125.3 | 25.4 | 12.7 | 13.3 |  |
| kd8-6 | PCA | 530-660 | 7 | 92.1 | 44.1 | 119.5 | 71.1 | 298.3 | 3.5 |  |
| kd8-7 | PCA | 560-690 | 9 | 221.1 | 31 | 226 | 3.8 | 23.7 | 10.8 |  |
| **Site kd09 (GPS: 36°51'39.51"N, 98°8'41.82"E)** | | | | | | | | | |  |
| kd9-1 | PCA | 400-680 | 12 | 121.5 | 30 | 152.7 | 3 8.2 | 25.8 | 8.7 |  |
| kd9-2 | PCA | 450-665 | 10 | 107.6 | 36.2 | 150.8 | 51.3 | 21 | 10.8 |  |
| kd9-3 | PCA | 500-630 | 7 | 116.6 | 36.2 | 156.5 | 45.1 | 753.2 | 2.2 |  |
| kd9-4 | GCA | 0-680 | 16 | 4.8 | 33.8 | 325.2 | 49 | 14.6 | 10 |  |
| kd9-5 | GCA | 0-600 | 15 | 196.1 | 47.5 | 210.3 | 10.1 | 26.3 | 7.6 |  |
| kd9-6 | PCA | 500-680 | 7 | 89.6 | 26.6 | 121.4 | 57.7 | 51.4 | 8.5 |  |
| site mean |  |  | 6 | 106.0 | 28.3 | 139.6 | 47.9 | 24.7 | 14.3 |  |
| **Site kd10 (GPS: 36°51'39.82"N, 98°8'41.62"E)** | | | | | | | | | |  |
| kd10-1 | PCA | 450-630 | 9 | 110.2 | 7.1 | 123.7 | 30.1 | 44.5 | 7.8 |  |
| kd10-2 | PCA | 500-670 | 12 | 113.3 | -6.3 | 117.2 | 17.7 | 13 | 12.5 |  |
| kd10-3 | GCA | 0-665 | 17 | 228.2 | 50.9 | 231.4 | 7.2 | 7.3 | 14.2 |  |
| kd10-5 | PCA | 350-600 | 10 | 99 | 1.8 | 109.5 | 32.6 | 40.3 | 7.7 |  |
| kd10-6 | GCA | 0-620 | 15 | 183.4 | 50.9 | 204.9 | 17.4 | 7.6 | 14.9 |  |
| kd10-7 | GCA | 0-580 | 13 | 53.2 | 83.6 | 237.7 | 52.3 | 20 | 9.5 |  |
| site mean |  |  | 6 | 108.7 | 1.2 | 118.2 | 26.4 | 36.4 | 12.1 |  |
| **Site kd11 (GPS: 36°51'39.81"N, 98°8'41.68"E)** | | | | | | | | | |  |
| kd11-1 | GCA | 0-680 | 17 | 213.4 | 41.2 | 39.5 | 0.3 | 9.2 | 12.4 |  |
| kd11-2 | GCA | 0-680 | 18 | 208.1 | 34.8 | 33.5 | 5.1 | 60 | 4.5 |  |
| kd11-3 | GCA | 0-630 | 15 | 196.1 | 35.6 | 24.9 | 0.5 | 10.2 | 12.6 |  |
| kd11-4 | GCA | 0-600 | 15 | 254.1 | 52.5 | 247.5 | 9.6 | 20.7 | 8.6 |  |
| kd11-5 | GCA | 0-650 | 17 | 226.1 | 49.5 | 229.9 | 5.9 | 8.1 | 13.4 |  |
| kd11-6 | GCA | 0-620 | 15 | 297.4 | 62.9 | 263.9 | 28.9 | 13.3 | 10.9 |  |
| kd11-7 | GCA | 0-650 | 16 | 322.2 | 68 | 266.3 | 40.2 | 7.1 | 14.9 |  |
| kd11-8 | PCA | 400-620 | 9 | 109.4 | 35.4 | 151 | 49.7 | 19.1 | 12.1 |  |
| site mean |  |  | 8 | 100.7 | 26.2 | 132.5 | 50.2 | 56.4 | 8.3 |  |
| **Site kd12(GPS: 36°51'40.08"N, 98°8'41.13"E)** | | | | | | | | | |  |
| kd12-2 | PCA | 350-690 | 16 | 121.5 | 12.8 | 137.5 | 26.8 | 86 | 4 |  |
| kd12-3 | PCA | 350-680 | 13 | 127.2 | 15.3 | 143.7 | 24.7 | 390.3 | 2.1 |  |
| kd12-4 | PCA | 350-680 | 13 | 125.4 | 12.6 | 140.3 | 24 | 205.2 | 2.9 |  |
| kd12-5 | PCA | 350-680 | 13 | 126.3 | 13 | 141.3 | 23.6 | 671.5 | 1.6 |  |
| site mean |  |  | 4 | 125.1 | 13.4 | 140.7 | 24.8 | 885.8 | 3.1 |  |
| **Site kd13 (GPS: 36°51'40.04"N, 98°8'55.50"E)** | | | | | | | | | |  |
| kd13-2 | PCA | 450-680 | 11 | 141.7 | 23.1 | 160 | 20 | 763.8 | 2 |  |
| kd13-4 | PCA | 350-680 | 13 | 126.5 | 17.2 | 144.7 | 26.4 | 390.3 | 2 |  |
| kd13-5 | PCA | 350-690 | 14 | 125.3 | 10 | 138.2 | 22.2 | 320.9 | 1.9 |  |
| kd13-6 | PCA | 350-680 | 13 | 119.8 | 24.9 | 146.6 | 36.3 | 334.3 | 2.4 |  |
| site mean |  |  | 4 | 128.3 | 19.0 | 147.5 | 26.4 | 53.9 | 12.6 |  |
| **Site kd14 (GPS: 36°51'39.97"N, 98° 8'40.73"E)** | | | | | | | | | |  |
| kd14-1 | PCA | 500-680 | 10 | 116.3 | 28.6 | 147.7 | 41 | 320.9 | 2.7 |  |
| kd14-2 | PCA | 500-680 | 11 | 127 | 22 | 149.1 | 29.3 | 334.3 | 2.5 |  |
| kd14-3 | PCA | 350-680 | 13 | 128.8 | 22.5 | 150.8 | 28.4 | 763.8 | 1.5 |  |
| kd14-4 | PCA | 350-680 | 13 | 128.2 | 19.7 | 148.1 | 26.9 | 390.3 | 2.1 |  |
| site mean |  |  | 4 | 125.2 | 23.3 | 149.0 | 31.4 | 151.8 | 7.5 |  |
| **Site kd15 (GPS: 36°51'37.16"N, 98°8'39.92"E)** | | | | | | | | | |  |
| kd15-3 | PCA | 500-680 | 10 | 124.9 | 25.3 | 150.6 | 32.9 | 345.9 | 2.6 |  |
| kd15-4 | PCA | 500-680 | 10 | 113.9 | 45.8 | 168.1 | 51.3 | 40.3 | 7.7 |  |
| kd15-5 | PCA | 500-700 | 11 | 98.4 | 35.8 | 143.7 | 57.5 | 41.2 | 7.2 |  |
| kd15-6 | PCA | 500-700 | 11 | 102 | 43.9 | 159.7 | 58.5 | 30.5 | 8.4 |  |
| kd15-7 | PCA | 500-700 | 12 | 133.1 | 35.7 | 165.6 | 33.7 | 63.2 | 5.5 |  |
| site mean |  |  | 5 | 115.0 | 38.1 | 157.9 | 47.2 | 32.1 | 13.7 |  |
| **Site kd16 (GPS: 36°51'37.07"N, 98°8'40.08"E)** | | | | | | | | | |  |
| kd16-1 | PCA | 500-700 | 12 | 113.7 | 31.7 | 149.3 | 44.6 | 138.5 | 3.7 |  |
| kd16-4 | PCA | 350-680 | 14 | 119.1 | 23.7 | 145 | 35.9 | 275 | 2.4 |  |
| kd16-5 | PCA | 350-680 | 13 | 122.3 | 29.3 | 152.7 | 37.2 | 299.1 | 2.4 |  |
| kd16-6 | PCA | 350-680 | 14 | 106.5 | 35.2 | 148.8 | 51.5 | 104.8 | 3.9 |  |
| kd16-7 | PCA | 350-680 | 12 | 120.6 | 25.3 | 147.5 | 35.9 | 196.9 | 3.1 |  |
| kd16-8 | PCA | 400-710 | 14 | 123.2 | 26 | 150 | 34.6 | 104.8 | 3.9 |  |
| kd16-9 | PCA | 400-710 | 14 | 114.3 | 25.8 | 143.4 | 40.6 | 90.5 | 4.2 |  |
| site mean |  |  | 7 | 117.3 | 28.2 | 148.1 | 40.1 | 152.0 | 4.9 |  |
| **Site kd17 (GPS: 36°51'53.65"N, 98°8'39.74"E)** | | | | | | | | | |  |
| kd17-1 | PCA | 500-700 | 12 | 295.5 | 71.3 | 256.3 | 34.4 | 46.9 | 6.4 |  |
| kd17-2 | PCA | 500-700 | 12 | 265 | 75.5 | 245 | 32.9 | 16.3 | 11.1 |  |
| kd17-3 | PCA | 400-710 | 14 | 240.5 | 75 | 238.1 | 31 | 35.1 | 6.8 |  |
| kd17-4 | PCA | 400-710 | 14 | 277 | 80.9 | 244.5 | 38.7 | 16.8 | 10 |  |
| kd17-6 | PCA | 500-710 | 13 | 275.4 | 74.4 | 248.5 | 33.1 | 33.2 | 7.3 |  |
| site mean |  |  | 5 | 271.6 | 76.2 | 246.4 | 34.2 | 169.1 | 5.9 |  |
| **Site kd18 (GPS: 36°51'37.02"N, 98°8'39.65"E)** | | | | | | | | | |  |
| kd18-1 | PCA | 500-710 | 13 | 288.7 | 78.4 | 248.6 | 38.1 | 430.2 | 2 |  |
| kd18-2 | PCA | 500-710 | 13 | 264 | 74 | 245.4 | 31.4 | 876.5 | 1.4 |  |
| kd18-3 | PCA | 500-710 | 13 | 254.6 | 77.4 | 241.6 | 33.9 | 355.7 | 2.2 |  |
| kd18-4 | PCA | 400-690 | 12 | 267.4 | 71.3 | 247.7 | 29.3 | 581.8 | 1.8 |  |
| kd18-5 | PCA | 400-690 | 12 | 264.1 | 71.1 | 246.7 | 28.7 | 389.9 | 2.2 |  |
| kd18-6 | PCA | 400-690 | 12 | 263.7 | 68.5 | 247.6 | 26.2 | 389.9 | 2.2 |  |
| site mean |  |  | 6 | 266.2 | 73.6 | 246.3 | 31.3 | 296.0 | 3.9 |  |
| **Site kd19 (GPS: 36°51'28.43"N, 98°8'30.42"E)** | | | | | | | | | |  |
| kd19-1 | PCA | 300-690 | 16 | 146.1 | -56.5 | 146.1 | -56.5 | 81.9 | 4.1 |  |
| kd19-2 | PCA | 350-690 | 16 | 3.6 | 55.6 | 3.6 | 55.6 | 309.2 | 2.1 |  |
| kd19-3 | PCA | 300-690 | 16 | 1.4 | -12.2 | 1.4 | -12.2 | 944.3 | 1.2 |  |
| kd19-4 | PCA | 350-690 | 15 | 161.2 | -33.1 | 161.2 | -33.1 | 14.5 | 10.4 |  |
| kd19-5 | PCA | 400-660 | 7 | 56.8 | 25.6 | 56.8 | 25.6 | 89.9 | 6.4 |  |
| kd19-6 | PCA | 400-680 | 10 | 6.2 | 56.4 | 6.2 | 56.4 | 1618.9 | 1.2 |  |
| kd19-7 | PCA | 300-680 | 10 | 343.7 | 48.2 | 343.7 | 48.2 | 1379.8 | 1.3 |  |
| kd19-8 | PCA | 400-680 | 9 | 216.6 | -71.1 | 216.6 | -71.1 | 818.2 | 1.8 |  |
| **Site kd20 (GPS: 36°51'28.63"N, 98°8'30.16"E)** | | | | | | | | | |  |
| kd20-1 | PCA | 300-690 | 15 | 140.9 | 55.7 | 173.7 | 49.6 | 506.3 | 1.7 |  |
| kd20-2 | PCA | 400-690 | 13 | 133.9 | 59.4 | 173.5 | 54.9 | 763.8 | 1.5 |  |
| kd20-3 | PCA | 400-690 | 13 | 144.2 | 59.6 | 179.9 | 51.4 | 594.9 | 1.7 |  |
| kd20-5 | PCA | 400-680 | 9 | 143.2 | 60.1 | 179.9 | 52.1 | 917.1 | 1.7 |  |
| kd20-7 | PCA | 400-680 | 9 | 140.6 | 55.7 | 173.5 | 49.7 | 1351.5 | 1.4 |  |
| site mean |  |  | 5 | 139.9 | 57.0 | 174.4 | 50.9 | 393.1 | 3.4 |  |
| **Site kd21 (GPS: 36°51'29.71"N, 98°8'30.20"E)** | | | | | | | | | |  |
| kd21-1 | PCA | 400-700 | 14 | 123.3 | 48.1 | 153.2 | 50.8 | 438.2 | 1.9 |  |
| kd21-2 | PCA | 300-700 | 13 | 132.8 | 52.8 | 165.3 | 50.5 | 1192.4 | 1.2 |  |
| kd21-3 | PCA | 400-700 | 13 | 128.6 | 52 | 161.3 | 51.5 | 1192.4 | 1.2 |  |
| kd21-4 | PCA | 300-700 | 14 | 126.6 | 52.6 | 160.5 | 52.8 | 1947.2 | 0.9 |  |
| kd21-5 | PCA | 400-680 | 9 | 126.9 | 53.9 | 162.1 | 53.7 | 1351.5 | 1.4 |  |
| kd21-6 | PCA | 400-680 | 9 | 125.3 | 50.8 | 157.6 | 52 | 1567.1 | 1.3 |  |
| kd21-7 | PCA | 400-680 | 9 | 134.7 | 54.4 | 168.2 | 51 | 460.8 | 2.4 |  |
| site mean |  |  | 7 | 128.2 | 52.1 | 161.2 | 51.8 | 614.6 | 2.4 |  |
| **Site kd22 (GPS: 36°51'29.67"N, 98°8'30.21"E)** | | | | | | | | | |  |
| kd22-1 | PCA | 350-690 | 13 | 125.4 | 51.9 | 158.8 | 52.8 | 1016.3 | 1.3 |  |
| kd22-2 | PCA | 0-690 | 18 | 126.7 | 51 | 158.8 | 51.6 | 609.7 | 1.4 |  |
| kd22-3 | PCA | 400-690 | 12 | 122.2 | 55.7 | 160.9 | 56.8 | 522.3 | 1.9 |  |
| kd22-4 | PCA | 300-690 | 12 | 141.7 | 39.6 | 161.4 | 36.2 | 652.1 | 1.7 |  |
| kd22-5 | PCA | 400-680 | 9 | 127 | 53.6 | 161.9 | 53.4 | 501.6 | 2.3 |  |
| kd22-6 | PCA | 400-680 | 9 | 121.3 | 52.6 | 156.4 | 55 | 315.9 | 2.9 |  |
| kd22-7 | PCA | 400-680 | 8 | 121.5 | 52.9 | 156.8 | 55.1 | 533.2 | 2.4 |  |
| site mean |  |  | 7 | 127.1 | 51.3 | 159.4 | 51.6 | 130.4 | 5.3 |  |
| **Site kd23 (GPS: 36°51'29.72"N, 98°8'30.24"E)** | | | | | | | | | |  |
| kd23-1 | PCA | 450-680 | 10 | 130.9 | 53.1 | 164.1 | 51.5 | 52.9 | 6.7 |  |
| kd23-2 | PCA | 530-680 | 7 | 128.3 | 55.9 | 165.5 | 54.5 | 41.3 | 9.5 |  |
| kd23-3 | PCA | 530-670 | 9 | 116.7 | 56.2 | 157.6 | 59.4 | 17.1 | 12.8 |  |
| kd23-4 | PCA | 500-670 | 9 | 127.4 | 46.8 | 155.3 | 48 | 65.7 | 6.4 |  |
| kd23-5 | PCA | 500-670 | 7 | 134.7 | 42.4 | 157.6 | 41.4 | 62.4 | 7.7 |  |
| kd23-6 | PCA | 500-680 | 8 | 138.9 | 55.4 | 172.1 | 50.1 | 27.3 | 10.8 |  |
| kd23-7 | PCA | 500-680 | 8 | 113 | 54.3 | 152 | 59.6 | 52.7 | 7.7 |  |
| site mean |  |  | 7 | 127.5 | 52.3 | 160.8 | 52.2 | 110.4 | 5.8 |  |
| **Site kd24 (GPS: 36°51'30.52"N, 98°8'29.90"E)** | | | | | | | | | |  |
| kd24-1 | PCA | 0-690 | 19 | 143 | 59.9 | 179.6 | 52 | 312.7 | 1.9 |  |
| kd24-2 | PCA | 400-690 | 12 | 138.5 | 58.9 | 175.7 | 52.8 | 960.8 | 1.4 |  |
| kd24-3 | PCA | 350-690 | 13 | 172 | 59.8 | 196.6 | 43.2 | 763.8 | 1.5 |  |
| kd24-6 | PCA | 400-680 | 9 | 131.3 | 62.6 | 176.4 | 57.9 | 601.5 | 2.1 |  |
| kd24-7 | PCA | 400-700 | 10 | 160.9 | 60.6 | 190.6 | 46.8 | 529.7 | 2.1 |  |
| kd24-8 | PCA | 400-700 | 10 | 148.9 | 64.2 | 187.7 | 53.2 | 911.5 | 1.6 |  |
| site mean |  |  | 6 | 149.2 | 61.7 | 185.1 | 51.3 | 116.7 | 6.2 |  |
| **Site kd25 (GPS: 36°51'30.48"N, 98°8'29.82"E)** | | | | | | | | | |  |
| kd25.2 | PCA | 350-690 | 13 | 152.5 | 66.5 | 192.2 | 53.8 | 275.7 | 2.5 |  |
| kd25.3 | PCA | 400-680 | 9 | 145.4 | 64.1 | 185.8 | 54.2 | 548.2 | 2.2 |  |
| kd25.4 | PCA | 450-710 | 9 | 109.7 | 56.7 | 153.1 | 62.6 | 230.1 | 3.4 |  |
| kd25.5 | PCA | 400-690 | 14 | 139.9 | 65.6 | 185.1 | 56.9 | 547 | 1.7 |  |
| kd25.6 | PCA | 500-700 | 9 | 142.7 | 67.4 | 188.9 | 57.2 | 917.1 | 1.7 |  |
| kd25.7 | PCA | 400-680 | 8 | 148.8 | 64.5 | 187.9 | 53.4 | 533.2 | 2.4 |  |
| kd25.8 | PCA | 400-680 | 9 | 129 | 61.5 | 173.3 | 58.1 | 116 | 4.8 |  |
| site mean |  |  | 7 | 137.0 | 64.5 | 182.0 | 57.2 | 109.2 | 5.8 |  |
| **Site sd01 (GPS: 36°2'50.78"N, 94°47'57.10"E)** | | | | | | | | | |  |
| sd1-3 | PCA | 250-550 | 9 | 325.5 | 34.6 | 328.6 | -25.1 | 34.4 | 8.9 |  |
| sd1-5 | GCA | 0-660 | 18 | 218.1 | 16.1 | 264.5 | 50.7 | 7.3 | 13.7 |  |
| sd1-6 | GCA | 0-675 | 22 | 207.1 | 18.9 | 264.5 | 61.6 | 13.1 | 8.9 |  |
| sd1-7 | GCA | 350-640 | 9 | 198.9 | 24.8 | 276.4 | 70 | 15.9 | 13.3 |  |
| site mean |  |  | 4 | 318.8 | 39.0 | 326.6 | -18.4 | 105.6 | 12.2 |  |
| **Site sd02 (GPS: 36°2'51.80"N, 94°47'57.68"E)** | | | | | | | | | |  |
| sd2-1 | GCA | 0-660 | 20 | 179.9 | 34.5 | 349 | 72.3 | 6.6 | 13.8 |  |
| sd2-2 | GCA | 0-680 | 19 | 177.4 | 39.2 | 356.2 | 67.8 | 6.3 | 14.6 |  |
| sd2-3 | PCA | 200-650 | 16 | 4.1 | 50.6 | 1.9 | -22.1 | 51.3 | 5.2 |  |
| sd2-4 | PCA | 200-700 | 22 | 8.6 | 47.1 | 5.7 | -25 | 10.8 | 9.9 |  |
| sd2-5 | PCA | 250-700 | 21 | 23.4 | 57.9 | 11 | -12 | 55.6 | 4.3 |  |
| sd2-7 | GCA | 0-560 | 14 | 198.2 | 28.2 | 300.6 | 67.5 | 16.8 | 10 |  |
| sd2-8 | GCA | 0-560 | 14 | 123.8 | 21.4 | 73.1 | 39.8 | 14.8 | 10.7 |  |
| site mean |  |  | 7 | 8.9 | 53.6 | 4.5 | -18.7 | 114.5 | 6.1 |  |
| **Site sd03 (GPS: 36°3'45.14"N, 94°44'53.30"E)** | | | | | | | | | |  |
| sd3-1 | PCA | 550-690 | 11 | 323.5 | 6.1 | 319.3 | -48.2 | 15.7 | 11.9 |  |
| sd3-2 | GCA | 350-680 | 13 | 219.2 | 25.7 | 252.6 | 32.4 | 8.4 | 15.2 |  |
| sd3-4 | PCA | 350-640 | 13 | 325.8 | 34 | 326.5 | -20.7 | 16.3 | 10.6 |  |
| sd3-5 | PCA | 350-680 | 14 | 299.4 | 31.7 | 303.4 | -16.6 | 17.8 | 9.7 |  |
| sd3-7 | PCA | 300-680 | 16 | 306.8 | 37.8 | 311.7 | -13.5 | 37.5 | 6.1 |  |
| sd3-8 | PCA | 350-680 | 15 | 303.8 | 29.1 | 305.8 | -20.6 | 33.5 | 6.7 |  |
| site mean |  |  | 6 | 313.8 | 27.6 | 314.2 | -25.1 | 25.8 | 13.7 |  |
| **Site sd04 (GPS: 36°6'3.45"N, 94°48'28.23"E)** | | | | | | | | | |  |
| sd4-1 | PCA | 0-700 | 25 | 2.5 | -1.8 | 359.7 | -23.8 | 33.2 | 5.1 |  |
| sd4-2 | PCA | 300-700 | 20 | 3 | -5.6 | 0 | -27.7 | 136.7 | 2.8 |  |
| sd4-3 | PCA | 350-700 | 19 | 3.1 | -5.8 | 359.4 | -27.8 | 181 | 2.5 |  |
| sd4-4 | PCA | 250-700 | 21 | 3 | -5.4 | 359.4 | -27.4 | 106.1 | 3.1 |  |
| site mean |  |  | 4 | 2.9 | -4.7 | 359.6 | -26.7 | 1742.8 | 2.2 |  |
| **Site sd05 (GPS: 36°6'3.48"N, 94°48'28.13"E)** | | | | | | | | | |  |
| sd5-1 | PCA | 480-700 | 16 | 198.9 | 26.2 | 194.2 | 50.5 | 237 | 2.4 |  |
| sd5-2 | PCA | 480-700 | 16 | 189.9 | 11.9 | 185.7 | 35.1 | 531.8 | 1.6 |  |
| sd5-4 | PCA | 400-680 | 10 | 194 | 12.8 | 190.4 | 36.6 | 154.3 | 3.9 |  |
| sd5-5 | PCA | 400-655 | 6 | 187.3 | 14 | 182.1 | 36.7 | 573.2 | 2.8 |  |
| sd5-6 | PCA | 400-700 | 9 | 184.6 | 36.4 | 169.7 | 57.7 | 27.5 | 10 |  |
| site mean |  |  | 5 | 191.0 | 20.3 | 185.1 | 43.8 | 46.8 | 11.3 |  |
| **Site sd06 (GPS: 36°6'3.52"N, 94°48'28.06"E)** | | | | | | | | | |  |
| sd6-2 | PCA | 400-700 | 18 | 170.4 | 31.5 | 154.6 | 48.7 | 93.1 | 3.6 |  |
| sd6-3 | PCA | 400-700 | 18 | 182.3 | 4 | 179 | 25.9 | 177.6 | 2.6 |  |
| sd6-4 | PCA | 400-700 | 18 | 189.3 | 13.3 | 184.7 | 36.3 | 110.6 | 3.3 |  |
| sd6-6 | PCA | 400-700 | 10 | 175.8 | 33.4 | 159.9 | 52.2 | 43.6 | 7.4 |  |
| sd6-7 | PCA | 400-710 | 19 | 178.1 | 6.2 | 173.9 | 27.1 | 126 | 3 |  |
| site mean |  |  | 5 | 179.5 | 17.8 | 171.9 | 38.5 | 27.7 | 14.8 |  |
| **Site sd07 (GPS: 36°6'3.10"N, 94°48'28.83"E)** | | | | | | | | | |  |
| sd7-3 | PCA | 400-700 | 18 | 176.7 | 5.1 | 172.7 | 25.6 | 177.6 | 2.6 |  |
| sd7-4 | PCA | 400-700 | 13 | 178.2 | 3.6 | 174.7 | 24.6 | 476.5 | 1.9 |  |
| sd7-5 | PCA | 400-700 | 12 | 184.2 | 4 | 181 | 26.3 | 581.8 | 1.8 |  |
| sd7-6 | PCA | 400-700 | 12 | 183.8 | -0.6 | 181.6 | 21.7 | 736 | 1.6 |  |
| site mean |  |  | 4 | 180.7 | 3.0 | 177.5 | 24.6 | 318.5 | 5.2 |  |
| **Site sd08 (GPS: 36°6'24.75"N, 94°53'1.34"E)** | | | | | | | | | |  |
| sd8-1 | PCA | 400-675 | 10 | 342.5 | 17 | 326.2 | 50.3 | 25.3 | 9.8 |  |
| sd8-4 | PCA | 250-680 | 17 | 177.9 | -55.6 | 58 | -80.8 | 40.1 | 5.7 |  |
| sd8-6 | PCA | 400-600 | 10 | 21.9 | 31.3 | 40 | 69.3 | 14.6 | 13.1 |  |
| sd8-8 | PCA | 450-680 | 12 | 150.9 | -26.7 | 123.3 | -52.2 | 12.1 | 13 |  |
| site mean |  |  | 4 | 352.1 | 34.1 | 323.6 | 69.4 | 10.7 | 29.4 |  |
| **Site sd09 (GPS: 36°6'24.79"N, 94°53'1.69"E)** | | | | | | | | | |  |
| sd9-1 | PCA | 300-650 | 13 | 24 | 23.6 | 37.7 | 61.5 | 26.5 | 8.2 |  |
| sd9-2 | PCA | 0-700 | 25 | 51.7 | 32 | 85.6 | 54.4 | 11.6 | 8.9 |  |
| sd9-4 | PCA | 0-620 | 18 | 22.7 | 46 | 79.5 | 80.6 | 117.6 | 3.2 |  |
| sd9-5 | PCA | 0-700 | 25 | 7.3 | 55 | 207.4 | 84.7 | 327.9 | 1.6 |  |
| sd9-6 | PCA | 0-620 | 18 | 2.6 | 53.2 | 242.5 | 84.4 | 331.6 | 1.9 |  |
| sd9-7 | PCA | 0-620 | 18 | 8.1 | 54 | 205.7 | 85.9 | 271.7 | 2.1 |  |
| sd9-8 | PCA | 0-620 | 18 | 9.9 | 56.7 | 190.5 | 83.3 | 299.4 | 2 |  |
| sd9-9 | PCA | 0-620 | 18 | 1.1 | 58.8 | 216.8 | 79.8 | 467.2 | 1.6 |  |
| site mean |  |  | 8 | 18.3 | 48.7 | 89.7 | 84.4 | 21.9 | 12.1 |  |
| **Site sd12 (GPS: 36°6'37.53"N, 94°53'22.22"E)** | | | | | | | | | |  |
| sd12-1 | PCA | 400-580 | 8 | 64.1 | 63.9 | 146.8 | 58.6 | 19.7 | 12.8 |  |
| sd12-2 | PCA | 300-550 | 6 | 72.7 | 56.5 | 134.8 | 53.2 | 58.9 | 8.8 |  |
| sd12-3 | PCA | 350-580 | 4 | 317.5 | 72.7 | 216.4 | 58 | 45.9 | 13.7 |  |
| sd12-4 | PCA | 250-550 | 7 | 48.7 | 72.1 | 165.8 | 62 | 36.7 | 10.1 |  |
| sd12-5 | PCA | 300-620 | 13 | 32.3 | 80.5 | 183.1 | 58.6 | 18.9 | 9.8 |  |
| sd12-6 | PCA | 300-620 | 13 | 341.9 | 74.7 | 205.8 | 62.7 | 24.2 | 8.6 |  |
| site mean |  |  | 6 | 39.4 | 74.9 | 174.0 | 62.3 | 23.7 | 14.0 |  |
| **Site sd13 (GPS: 36°6'37.08"N, 94°53'21.35"E)** | | | | | | | | | |  |
| sd13-1 | PCA | 510-550 | 3 | 145.3 | 67.2 | 171.3 | 32 | 77.5 | 14.1 |  |
| sd13-2 | PCA | 0-530 | 9 | 76.6 | 79.4 | 173.7 | 53.1 | 42.4 | 8 |  |
| sd13-3 | PCA | 300-560 | 8 | 264.5 | 82.9 | 200.2 | 47.6 | 26.3 | 11 |  |
| sd13-4 | PCA | 200-560 | 11 | 156.2 | 59.8 | 172.3 | 23.1 | 22.2 | 9.9 |  |
| sd13-5 | PCA | 550-620 | 6 | 273.9 | 84.2 | 198.7 | 49.1 | 45.8 | 10 |  |
| sd13-6 | PCA | 510-600 | 5 | 153.1 | 72.3 | 177.1 | 35 | 32.6 | 13.6 |  |
| site mean |  |  | 6 | 150.4 | 82.5 | 183.4 | 44.0 | 35.3 | 13.1 |  |

Notes: GCA, great-circle fit; PCA, vector line fit; Demagnetization Steps, temperature step range over which the ChRM is defined; N, number of consecutive demagnetization steps used to define the ChRM; For the PCA and GCA results, D and I denote the declination and inclination of the ChRM direction and of the pole that is perpendicular to the plane of great circle, respectively. The letters _g_ and _s_ indicate geographic and tilt-corrected coordinate systems, respectively; κ, the best estimate of the precision parameter; MAD, maximum angular deviation of the ChRM fit.

Table S4. Whole-rock geochemistry data of the Devonian volcanics of Qaidam-Kunlun.

| Sample | kd01-2 | kd02-6 | kd03-4 | kd09-6 | kd10-1 | kd21-4 | kd22-5 | kd24-2 | kd24-6 | sd04-3 | sd06-4 |
| --- | --- | --- | --- | --- | --- | --- | --- | --- | --- | --- | --- |
| SiO_2_ | 50.44 | 50.42 | 50.44 | 50.42 | 50.39 | 52.35 | 52.34 | 52.27 | 53.35 | 67.48 | 67.72 |
| TiO_2_ | 1.06 | 1.06 | 1.06 | 1.05 | 1.05 | 1.26 | 1.27 | 1.26 | 1.26 | 0.43 | 0.43 |
| Al_2_O_3_ | 12.72 | 12.72 | 12.72 | 12.72 | 12.75 | 13.84 | 13.85 | 13.84 | 14.02 | 15.02 | 15.10 |
| Fe_2_O_3_T | 8.19 | 8.20 | 8.21 | 8.21 | 8.19 | 7.93 | 7.95 | 7.95 | 8.31 | 3.38 | 3.30 |
| MnO | 0.13 | 0.13 | 0.13 | 0.13 | 0.13 | 0.11 | 0.11 | 0.11 | 0.10 | 0.04 | 0.04 |
| MgO | 10.14 | 10.15 | 10.18 | 10.15 | 10.15 | 6.54 | 6.54 | 6.56 | 5.44 | 2.09 | 2.04 |
| CaO | 8.92 | 8.97 | 8.93 | 8.91 | 8.93 | 9.25 | 9.21 | 9.24 | 8.44 | 3.50 | 3.40 |
| Na_2_O | 2.16 | 2.16 | 2.15 | 2.17 | 2.16 | 2.62 | 2.63 | 2.62 | 3.95 | 5.44 | 5.34 |
| K_2_O | 2.56 | 2.57 | 2.57 | 2.57 | 2.57 | 3.01 | 3.01 | 3.01 | 1.91 | 0.96 | 0.94 |
| LOI | 3.02 | 2.97 | 2.95 | 2.99 | 3.01 | 2.11 | 2.12 | 2.17 | 2.31 | 1.49 | 1.52 |
| Y | 22.2 | 22.5 | 22.0 | 23.2 | 23.9 | 25.6 | 26.3 | 26.3 | 27.7 | 11.6 | 10.7 |
| La | 57.2 | 56.9 | 56.6 | 56.4 | 56.4 | 90.7 | 86.3 | 88.8 | 92.8 | 12.3 | 11.8 |
| Ce | 121 | 120 | 119 | 119 | 119 | 201 | 186 | 193 | 201 | 27.7 | 26.3 |
| Pr | 15.6 | 15.5 | 15.3 | 15.4 | 15.4 | 26.1 | 24.6 | 25.8 | 26.7 | 3.36 | 3.13 |
| Nd | 61.4 | 61.0 | 60.8 | 61.1 | 60.5 | 101 | 96.4 | 100 | 105 | 14.1 | 13.0 |
| Sm | 9.91 | 9.95 | 9.90 | 9.85 | 9.68 | 15.0 | 14.4 | 15.0 | 15.6 | 2.84 | 2.62 |
| Eu | 2.48 | 2.45 | 2.46 | 2.46 | 2.44 | 3.76 | 3.57 | 3.73 | 3.69 | 0.78 | 0.75 |
| Gd | 8.03 | 8.05 | 8.01 | 8.05 | 7.88 | 11.8 | 11.4 | 11.8 | 12.3 | 2.58 | 2.41 |
| Tb | 1.07 | 1.07 | 1.06 | 1.07 | 1.06 | 1.43 | 1.40 | 1.43 | 1.48 | 0.41 | 0.38 |
| Dy | 5.02 | 5.09 | 5.03 | 5.06 | 5.00 | 6.22 | 6.09 | 6.15 | 6.38 | 2.32 | 2.07 |
| Ho | 0.84 | 0.86 | 0.84 | 0.83 | 0.84 | 0.97 | 1.00 | 1.01 | 1.04 | 0.42 | 0.38 |
| Er | 2.08 | 2.09 | 2.07 | 2.10 | 2.11 | 3.27 | 2.46 | 2.47 | 2.65 | 1.07 | 0.95 |
| Tm | 0.32 | 0.32 | 0.31 | 0.32 | 0.33 | 0.35 | 0.36 | 0.35 | 0.37 | 0.18 | 0.16 |
| Yb | 1.89 | 1.89 | 1.85 | 1.89 | 1.92 | 2.13 | 2.14 | 2.14 | 2.27 | 1.09 | 0.99 |
| Lu | 0.28 | 0.28 | 0.27 | 0.27 | 0.28 | 0.30 | 0.31 | 0.30 | 0.33 | 0.17 | 0.15 |
| Li | 27.7 | 27.9 | 28.1 | 27.9 | 28.8 | 24.9 | 23.9 | 26.2 | 17.9 | 10.6 | 12.4 |
| Be | 2.75 | 2.77 | 2.78 | 2.91 | 2.96 | 2.45 | 2.77 | 2.46 | 2.66 | 1.09 | 0.99 |
| Sc | 28.3 | 31.7 | 32.8 | 27.9 | 27.9 | 33.6 | 29.7 | 32.4 | 30.6 | 7.08 | 6.67 |
| V | 153 | 152 | 149 | 156 | 156 | 199 | 192 | 198 | 200 | 54.1 | 52.3 |
| Cr | 541 | 611 | 573 | 534 | 493 | 250 | 201 | 243 | 203 | 25.6 | 37.6 |
| Co | 41.6 | 41.4 | 40.5 | 43.0 | 42.2 | 34.6 | 33.7 | 35.3 | 35.9 | 9.02 | 8.59 |
| Ni | 223 | 220 | 215 | 227 | 227 | 78.6 | 75.8 | 77.8 | 78.8 | 12.5 | 11.4 |
| Cu | 47.6 | 48.1 | 47.0 | 50.1 | 48.8 | 28.8 | 27.7 | 28.0 | 27.8 | 4.30 | 2.72 |
| Zn | 75.3 | 76.0 | 74.4 | 79.4 | 81.8 | 80.1 | 75.9 | 77.9 | 76.3 | 54.9 | 52.8 |
| Ga | 15.3 | 15.6 | 15.3 | 16.2 | 16.4 | 18.4 | 17.9 | 18.6 | 18.7 | 16.1 | 16.3 |
| Rb | 99.6 | 103 | 102 | 107 | 111 | 107 | 101 | 101 | 65.1 | 48.2 | 43.6 |
| Sr | 665 | 683 | 657 | 697 | 694 | 1483 | 1437 | 1485 | 1619 | 223 | 223 |
| Zr | 226 | 244 | 235 | 248 | 256 | 256 | 255 | 252 | 263 | 153 | 146 |
| Nb | 13.3 | 13.3 | 13.2 | 13.7 | 13.8 | 11.0 | 10.4 | 10.6 | 11.3 | 3.17 | 2.86 |
| Mo | 0.67 | 0.74 | 1.27 | 0.63 | 0.62 | 0.38 | 0.32 | 0.36 | 0.44 | 0.16 | 0.16 |
| Cd | 4.15 | 2.40 | 3.81 | 2.04 | 1.93 | 0.12 | 0.12 | 0.17 | 0.075 | 0.059 | 0.056 |
| In | 0.064 | 0.064 | 0.064 | 0.065 | 0.065 | 0.085 | 0.074 | 0.074 | 0.077 | 0.034 | 0.031 |
| Cs | 4.65 | 4.68 | 4.63 | 4.83 | 5.12 | 8.89 | 8.70 | 9.14 | 2.29 | 2.75 | 2.70 |
| Ba | 1089 | 1082 | 1081 | 1131 | 1143 | 1784 | 1751 | 1822 | 1272 | 137 | 133 |
| Hf | 6.99 | 7.38 | 7.35 | 7.32 | 7.39 | 8.21 | 8.20 | 8.07 | 8.50 | 4.59 | 4.47 |
| Ta | 0.97 | 0.95 | 0.97 | 0.97 | 1.11 | 0.80 | 0.79 | 0.76 | 0.83 | 0.53 | 0.24 |
| W | 1.41 | 1.48 | 1.54 | 1.62 | 3.07 | 3.93 | 3.71 | 3.98 | 5.68 | 1.91 | 0.79 |
| Tl | 0.77 | 0.78 | 0.78 | 0.75 | 0.78 | 0.83 | 0.68 | 0.75 | 0.41 | 0.23 | 0.21 |
| Pb | 20.6 | 20.7 | 20.6 | 20.7 | 21.0 | 36.1 | 34.3 | 35.8 | 44.3 | 7.61 | 6.91 |
| Bi | 0.12 | 0.12 | 0.11 | 0.13 | 0.14 | 0.25 | 0.23 | 0.21 | 0.18 | 0.17 | 0.044 |
| Th | 24.2 | 24.4 | 24.2 | 24.5 | 24.1 | 21.9 | 21.0 | 21.7 | 23.0 | 3.29 | 2.87 |
| U | 5.24 | 5.31 | 5.26 | 5.24 | 5.30 | 5.33 | 5.12 | 5.34 | 6.78 | 0.94 | 0.82 |
| Eu/Eu* | 1.53 | 1.52 | 1.53 | 1.53 | 1.54 | 1.53 | 1.52 | 1.53 | 1.49 | 1.60 | 1.64 |
| Mg# | 71.17 | 71.17 | 71.21 | 71.16 | 71.21 | 62.17 | 62.13 | 62.20 | 56.62 | 55.24 | 55.31 |
| La/Nb | 4.30 | 4.28 | 4.29 | 4.12 | 4.09 | 8.25 | 8.30 | 8.38 | 8.21 | 3.88 | 4.13 |

Eu/Eu^*^ = (Eu/0.168)/SQRT((Sm/0.444) × (Gd/0.596)); Mg# = 100 × Molar Mg2+/(Mg^2+^+TFe^2+^); TFeO* = 0.8998 × TFe_2_O_3_

**Table S5.** **Compilation of Silurian-Triassic paleomagnetic results from the Qaidam-Kunlun and Qiangtang terranes, together with APWPs of India, North China, and Tarim and their corresponding paleolatitudes.**

| **ID** | **Lithology** | **Area/Formation** | **Age** | **Min_age** | **Max_age** | **Slat** | **Slon** | **N/n** | **Plat** | **Plon** | **A_95_** | **PaleolatR (°N)** | **Test** | **Reference** |
| --- | --- | --- | --- | --- | --- | --- | --- | --- | --- | --- | --- | --- | --- | --- |
| **Qaidam-Kunlun terrane** | | | | | | | | | | | | | | |
| WL | volcanic rocks | Wulan-Dangangou/Maoniushan Fm. | 411 | 404.8 | 417.2 | 36.1 | 94.9 | 30/159 | 41.6 | 163.4 | 6.8 | -25.6 [-19.3, -33.2] | F, R, C | this study |
| HT | limestone | Delingha/Huaitoutala Fm. | 338.5 | 330.3 | 346.7 | 37.3 | 96.4 | 15/108 | 3.2 | 327.5 | 4.8 | 29.4 [21.8, 39.0] | F | Cao *et al*. [8] |
| KK | limestone | Delingha/Keluke Fm. | 311.1 | 298.9 | 323.2 | 37.4 | 96.1 | 8/61 | 15.1 | 312.2 | 5.4 | 27.8 [22.0, 34.7] | / | Cao *et al*. [8] |
| DG | tuff | Dagangou/ | 258 | 251 | 265 | 36.1 | 94.9 | 39/212 | 81.3 | 273.7 | 3.1 | 27.4 [24.3, 30.5] | F, R, B | Song *et al*. [9] |
| TJ | sandstone | Tianjun/Nuoyinhe Fm. | 255.7 | 251.9 | 259.5 | 37.5 | 99.1 | 9/56 | 64.0 | 342.4 | 5.9 | 24.2 [18.5, 30.9] | R | Xu *et al*. [10] |
| NY | sandstone | Tianjun/Nuoyinhe Fm. | 255.7 | 251.9 | 259.5 | 37.5 | 99.1 | /199 | 77.6 | 332.8 | 2.1 | 28.9 [27.2, 30.6] | F | Wang *et al*. [11] |
| QD | lava | Qimantagh-Dulan/ | 239.5 | 236 | 243 | 36.4-  36.7 | 93.5-  98.2 | 28/164 | 57.6 | 178.2 | 4 | 33.2 [30.3, 36.4] | F | Wang *et al*. [12] |
| **Qiangtang terrane** | | | | | | | | | | | | | | |
| KX | lava | Tuotuohe/Kaixinling | 296.9 | 295 | 298.8 | 34.1 | 92.4 | 14/134 | 21.7 | 232.9 | 8.9 | -19.9 [-11.5, -27.2] | F | Song *et al*. [13] |
| RM | lava | Rongma/Lugu Fm. | 265 | 260 | 270 | 33.2 | 86.9 | 9/73 | 34.1 | 274.7 | 5.8 | -19.8 [-14.3, -25.8] | C | Wei *et al*. [14] |
| TT | lava | Tuotuohe/Nayixiong Fm. | 259 | -- | -- | 33.9 | 91.9 | 28/ | 13.6 | 2.4 | 5.6 | -6.0 [-0.4, -11.6] | F, R | Ma *et al*. [15] |
| ZD | lava | Zaduo/Gadikao Fm. | 251 | 248 | 254 | 32.5 | 95.2 | 29/257 | 59.7 | 228.2 | 3.3 | 13.2 [9.6, 17.1] | F, R | Guan *et al*. [16] |
| YS | lava | Yanshiping/ | 241 | 240 | 242 | 33.5 | 92 | 20/219 | 62.2 | 196.4 | 5.6 | 26.5 [22.5, 33.9] | F | Song *et al*. [17] |
| GM | limestone+lava | Gumu/Zuoqingco Fm. | 236 | 235 | 237 | 33.1 | 85.3 | 21/144 | 32.3 | 170.4 | 4.8 | 29.1 [23.8, 33.9] | F | Wei *et al*. [18] |
| BT | lava | Duocai/Batang Fm. | 224.5 | 222 | 227 | 33.8 | 95.2 | 11/ | 57.6 | 176.4 | 7.8 | 34.2 [27.3, 42.4] | F, R | Yu *et al*. [19] |
| XK | lava | Xiaochaka/ | 222 | 222 | 222 | 33 | 87.8-  88.4 | 25/ | 24.4 | 284.5 | 4.3 | 28.8 [24.8, 32.9] | F, R | Wei *et al*. [20] |
| JP | lava | Tuotuohe/Jiapila Fm. | 208.5 | 203.6 | 213.3 | 34.1 | 92.4 | 29/240 | 64.0 | 174.7 | 6.6 | 36.3 [30.1, 41.9] | F, R | Song *et al*. [21] |
| **Indian Gondwana** | | | | | | | | | | | | | | |
|  |  |  |  |  |  | **Ma** | **Plat** | **Plon** | **A_95_** | **PaleolatR (°N)** | | **Reference** | |  |
|  |  |  |  |  |  | 210 | 19.7 | 305 | 1.3 | -26.6 | | Vaes *et al*. [22] | |  |
|  |  |  |  |  |  | 220 | 19 | 300.8 | 1.1 | -29.0 | | Vaes *et al*. [22] | |  |
|  |  |  |  |  |  | 230 | 17.4 | 298.3 | 1.6 | -31.4 | | Vaes *et al*. [22] | |  |
|  |  |  |  |  |  | 240 | 8.4 | 293.4 | 2.9 | -41.8 | | Vaes *et al*. [22] | |  |
|  |  |  |  |  |  | 250 | 3.7 | 292.5 | 2.1 | -46.4 | | Vaes *et al*. [22] | |  |
|  |  |  |  |  |  | 260 | 3 | 292.4 | 1.6 | -47.2 | | Vaes *et al*. [22] | |  |
|  |  |  |  |  |  | 270 | 4.1 | 289.5 | 1.6 | -47.2 | | Vaes *et al*. [22] | |  |
|  |  |  |  |  |  | 280 | 2.4 | 285.4 | 1.3 | -50.0 | | Vaes *et al*. [22] | |  |
|  |  |  |  |  |  | 290 | 0.2 | 284.3 | 2 | -52.4 | | Vaes *et al*. [22] | |  |
|  |  |  |  |  |  | 300 | 0.4 | 278.7 | 2.5 | -53.2 | | Vaes *et al*. [22] | |  |
|  |  |  |  |  |  | 310 | 1.8 | 271.4 | 1.9 | -51.9 | | Vaes *et al*. [22] | |  |
|  |  |  |  |  |  | 320 | 6.6 | 269.4 | 2.4 | -47.0 | | Vaes *et al*. [22] | |  |
|  |  |  |  |  |  | 330 | -3.1 | 269 | - | -56.6 | | van Hinsbergen *et al*. [23] | |  |
|  |  |  |  |  |  | 340 | -8.6 | 229.9 | - | -40.8 | | van Hinsbergen *et al*. [23] | |  |
|  |  |  |  |  |  | 350 | -8.6 | 229.9 | - | -40.8 | | van Hinsbergen *et al*. [23] | |  |
|  |  |  |  |  |  | 360 | 11.5 | 240.6 | 11.2 | -32.5 | | van Hinsbergen *et al*. [23] | |  |
|  |  |  |  |  |  | 370 | 8.2 | 239.6 | 16.9 | -34.7 | | van Hinsbergen *et al*. [23] | |  |
|  |  |  |  |  |  | 380 | 13.1 | 236.8 | 36.7 | -29.1 | | van Hinsbergen *et al*. [23] | |  |
|  |  |  |  |  |  | 390 | 26.5 | 230.9 | 18 | -28 | | van Hinsbergen *et al*. [23] | |  |
|  |  |  |  |  |  | 400 | 17.7 | 245.2 | 18 | -48.3 | | van Hinsbergen *et al*. [23] | |  |
| **Eurasia** | | | | | | | | | | | | | | |
|  |  |  |  |  |  | 210 | 56.8 | 113.9 | 1.3 | 65.7 | | Vaes *et al*. [22] | |  |
|  |  |  |  |  |  | 220 | 54.7 | 122.1 | 1.1 | 63.7 | | Vaes *et al*. [22] | |  |
|  |  |  |  |  |  | 230 | 53.7 | 127.5 | 1.6 | 61.4 | | Vaes *et al*. [22] | |  |
|  |  |  |  |  |  | 240 | 52.9 | 144.8 | 2.9 | 51.6 | | Vaes *et al*. [22] | |  |
|  |  |  |  |  |  | 250 | 53 | 152.7 | 2.1 | 46.8 | | Vaes *et al*. [22] | |  |
|  |  |  |  |  |  | 260 | 53 | 154 | 1.6 | 46.1 | | Vaes *et al*. [22] | |  |
|  |  |  |  |  |  | 270 | 50 | 153 | 1.6 | 45.6 | | Vaes *et al*. [22] | |  |
|  |  |  |  |  |  | 280 | 46.2 | 156.4 | 1.3 | 43.8 | | Vaes *et al*. [22] | |  |
|  |  |  |  |  |  | 290 | 45.3 | 159.6 | 2 | 41.5 | | Vaes *et al*. [22] | |  |
|  |  |  |  |  |  | 300 | 39.7 | 159.8 | 2.5 | 39.8 | | Vaes *et al*. [22] | |  |
|  |  |  |  |  |  | 310 | 32.4 | 158.5 | 1.9 | 38.3 | | Vaes *et al*. [22] | |  |
|  |  |  |  |  |  | 320 | 30.1 | 153 | 2.4 | 41.6 | | Vaes *et al*. [22] | |  |
| **North China** | | | | | | | | | | | | | | |
|  |  |  |  |  |  | 210 | 61.9 | 7.1 | – | 32.3 | | Huang *et al*. [24] | |  |
|  |  |  |  |  |  | 220 | 61.9 | 7.1 | – | 32.3 | | Huang *et al*. [24] | |  |
|  |  |  |  |  |  | 240 | 58.7 | 358.4 | 3.7 | 27.1 | | Huang *et al*. [24] | |  |
|  |  |  |  |  |  | 250 | 58.7 | 358.4 | 3.7 | 27.1 | | Huang *et al*. [24] | |  |
|  |  |  |  |  |  | 260 | 54.3 | 354.2 | 4.8 | 23.0 | | Huang *et al*. [24] | |  |
|  |  |  |  |  |  | 270 | 48.7 | 358 | 4.2 | 22.2 | | Huang *et al*. [24] | |  |
|  |  |  |  |  |  | 300 | 33.3 | 10.2 | 16.7 | 22.7 | | Huang *et al*. [24] | |  |
|  |  |  |  |  |  | 310 | 33.3 | 10.2 | 16.7 | 22.7 | | Huang *et al*. [24] | |  |
|  |  |  |  |  |  | 330 | 10.5 | 14 | 6.2 | 13.5 | | Huang *et al*. [24] | |  |
|  |  |  |  |  |  | 340 | 10.5 | 14 | 6.2 | 13.5 | | Huang *et al*. [24] | |  |
|  |  |  |  |  |  | 370 | 56 | 336 | 9.2 | 15.7 | | Huang *et al*. [24] | |  |
|  |  |  |  |  |  | 380 | 56 | 336 | 9.2 | 15.7 | | Huang *et al*. [24] | |  |
|  |  |  |  |  |  | 430 | 60.1 | 339 | 11.2 | 19.6 | | Huang *et al*. [24] | |  |
|  |  |  |  |  |  | 440 | 60.1 | 339 | 11.2 | 19.6 | | Huang *et al*. [24] | |  |
| **Tarim** | | | | | | | | | | | | | | |
|  |  |  |  |  |  | 210 | 166.8 | 52.1 | 7.0 | 38.3 | | Huang *et al*. [24] | |  |
|  |  |  |  |  |  | 220 | 163.7 | 55.6 | - | 40.6 | | Huang *et al*. [24] | |  |
|  |  |  |  |  |  | 230 | 160 | 59 | 13 | 42.9 | | Huang *et al*. [24] | |  |
|  |  |  |  |  |  | 240 | 171.8 | 52.7 | - | 35.4 | | Huang *et al*. [24] | |  |
|  |  |  |  |  |  | 260 | 180.5 | 55.6 | 3.1 | 31.4 | | Huang *et al*. [24] | |  |
|  |  |  |  |  |  | 270 | 182.7 | 58.5 | 3.5 | 31.2 | | Huang *et al*. [24] | |  |
|  |  |  |  |  |  | 280 | 176.1 | 56 | 10.4 | 33.9 | | Huang *et al*. [24] | |  |
|  |  |  |  |  |  | 290 | 175.6 | 57.6 | 7.6 | 34.6 | | Huang *et al*. [24] | |  |
|  |  |  |  |  |  | 300 | 173.6 | 62.4 | - | 36.5 | | Huang *et al*. [24] | |  |
|  |  |  |  |  |  | 320 | 177 | 60.6 | - | 34.6 | | Huang *et al*. [24] | |  |
|  |  |  |  |  |  | 370 | 156.5 | 13.1 | 6 | 30.5 | | Huang *et al*. [24] | |  |
|  |  |  |  |  |  | 400 | 152.7 | 9.8 | 4.5 | 31.6 | | Huang *et al*. [24] | |  |
|  |  |  |  |  |  | 410 | 158.9 | 13.2 | - | 28.6 | | Huang *et al*. [24] | |  |
|  |  |  |  |  |  | 420 | 161.8 | 14.3 | - | 26.9 | | Huang *et al*. [24] | |  |
|  |  |  |  |  |  | 430 | 165.5 | 15.7 | - | 24.7 | | Huang *et al*. [24] | |  |
|  |  |  |  |  |  | 450 | 185.3 | −33.5 | 3.5 | -19.3 | | Huang *et al*. [24] | |  |

Note. ID, paleopoles abbreviation; Fm, Formation; Slat/Slon, latitude/longitude of the sampling area; N/n, number of sites/samples used to calculate the Fisherian mean; Plat/Plon, latitude/longitude of the paleopole; A_95_, radius of the circle of 95% confidence; PaleolatR, the paleolatitudes of the reference point (36.1°N, 94.9°E) calculated via website tools (https://www.paleomagnetism.org); F/R/C/B means a positive fold/reversal/conglomerate/baked contact test.

Table S6. Qaidam-Kunlun Devonian volcanic rocks FORC smoothing parameters

| **Sample** | **Optimum SF** |
| --- | --- |
| kd15-4 | 5 |
| kd16-10 | 8 |
| kd17-4 | 7 |
| kd21-2 | 8 |
| kd24-7 | 10 |

Measurement parameters: averaging time-0.5 msec; Hb1-0.1 mT; Hb2-0.1 mT; Hc2-0.1 mT; H-sat-1 T; number of FORCs-100

**REFERECES**

1. Maxbauer DP, Feinberg JM, Fox DL. MAX UnMix: A web application for unmixing magnetic coercivity distributions. *Comput Geosci* 2016; **95**:140-145.

2. Harrison RJ, Feinberg JM. FORCinel: An improved algorithm for calculating first-order reversal curve distributions using locally weighted regression smoothing. *Geochem Geophys Geosyst* 2008; **9**: Q05016.

3. Egli R. VARIFORC: An optimized protocol for calculating non-regular first-order reversal curve (FORC) diagrams. *Glob Planet Change* 2013; **110**:302-320.

4. Heslop D, Roberts AP. Estimation of significance levels and confidence intervals for first-order reversal curve distributions. *Geochem Geophys Geosyst* 2012; **13:** Q12z40.

5. Roberts AP, Hu P, Harrison RJ *et al*. Domain State Diagnosis in Rock Magnetism: Evaluation of Potential Alternatives to the Day Diagram. *J Geophys Res Solid Earth* 2019; **124**:5286-5314.

6. Watson GS, Enkin RJ. The fold test in paleomagnetism as a parameter estimation problem. *Geophys Res Lett* 1993; **20**: 2135-2137.

7. Enkin RJ. The direction-correction tilt test: an all-purpose tilt/fold test for paleomagnetic studies. *Earth Planet Sci Lett* 2003; **212**: 151-166.

8. Cao Y, Sun Z, Li H *et al*. New Late Cretaceous paleomagnetic data from volcanic rocks and red beds from the Lhasa terrane and its implications for the paleolatitude of the southern margin of Asia prior to the collision with India. *Gondwana Res* 2017; **41**: 337-351.

9. Song P, Ding L, Zhang L *et al*. Defining the main Paleo-Tethys suture in Tibet: First Permian paleomagnetic insights from the Eastern Kunlun Range. *Earth Planet Sci Lett* 2026; **681**: 119925.

10. Xu W, Sun ZM, Pei JL *et al*. New Late Permian paleomagnetic results from Qaidam block and tectonic implications. *Acta Petrol Sin (Eng)* 2011; **27**: 3479-3486.

11. Wang T, Zhou YN, van Hinsbergen DJJ *et al*. Paleomagnetic Evidence for a Late Permian Qaidam-North China Connection, and the Cryptic Final Mesozoic Intra-Asian Suture. *J Geophys Res Solid Earth* 2025; **130**: e2025JB031123.

12. Wang B, Huang BC, Yang ZY *et al*. Palaeomagnetic results from Early Mesozoic strata in the Qaidam Basin and their implications for the formation of the Northern China Domain. *Geophys J Int* 2024; **236**: 1621-1635.

13. Song P, Ding L, Li Z *et al*. An early bird from Gondwana: Paleomagnetism of Lower Permian lavas from northern Qiangtang (Tibet) and the geography of the Paleo-Tethys. *Earth Planet Sci Lett* 2017; **475**: 119-133.

14. Wei B, Cheng X, Domeier M *et al*. Placing another piece of the Tethyan puzzle: The first Paleozoic paleomagnetic data from the South Qiangtang block and its paleogeographic implications. *Tectonics* 2022; **41**: e2022TC007355.

15. Ma Y, Wang Q, Wang J *et al*. Paleomagnetic Constraints on the Origin and Drift History of the North Qiangtang Terrane in the Late Paleozoic. *Geophys Res Lett* 2019; **46**: 689-697.

16. Guan C, Yan M, Zhang W *et al*. Paleomagnetic and chronologic data bearing on the Permian/Triassic boundary position of Qamdo in the Eastern Qiantang Terrane: Implications for the closure of the Paleo-Tethys. *Geophys Res Lett* 2021; **48**: e2020GL092059.

17. Song P, Ding L, Lippert PC *et al*. Paleomagnetism of Middle Triassic Lavas From Northern Qiangtang (Tibet): Constraints on the Closure of the Paleo-Tethys Ocean. *J Geophys Res Solid Earth* 2020; **125**: e2019JB017804.

18. Wei BT, Cheng X, Domeier M *et al*. A Cimmerian keystone: Middle-late Triassic paleomagnetic and calcite geochronologic constraints on the South Qiangtang Block. *Earth Planet Sci Lett* 2025; **664**: 119442.

19. Yu L, Yan M, Domeier M *et al*. New Paleomagnetic and Chronological Constraints on the Late Triassic Position of the Eastern Qiangtang Terrane: Implications for the Closure of the Paleo-Jinshajiang Ocean. *Geophys Res Lett* 2022; **49**: e2021GL096902.

20. Wei B, Cheng X, Domeier M *et al*. Paleomagnetism of Late Triassic Volcanic Rocks From the South Qiangtang Block, Tibet: Constraints on Longmuco-Shuanghu Ocean Closure in the Paleo-Tethys Realm. *Geophys Res Lett* 2023; **50**: e2023GL104759.

21. Song P, Ding L, Li Z *et al*. Late Triassic paleolatitude of the Qiangtang block: Implications for the closure of the Paleo-Tethys Ocean. *Earth Planet Sci Lett* 2015; **424**: 69-83.

22. Vaes B, van Hinsbergen DJJ, van de Lagemaat SHA *et al*. A global apparent polar wander path for the last 320 Ma calculated from site-level paleomagnetic data. *Earth Sci Rev* 2023; **245**: 104547.

23. van Hinsbergen DJJ, Lippert PC, Dupont-Nivet G *et al*. Greater India Basin hypothesis and a two-stage Cenozoic collision between India and Asia. *Proc Natl Acad Sci U S A* 2012; **109**: 7659-7664.

24. Huang B, Yan Y, Piper JDA *et al*. Paleomagnetic constraints on the paleogeography of the East Asian blocks during Late Paleozoic and Early Mesozoic times. *Earth Sci Rev* 2018; **186**: 8-36.
